# A systematic review and network meta-analysis of existing pharmacologic therapies in patients with idiopathic sudden sensorineural hearing loss

**DOI:** 10.1371/journal.pone.0221713

**Published:** 2019-09-09

**Authors:** Nadera Ahmadzai, Shaun Kilty, Wei Cheng, Leila Esmaeilisaraji, Dianna Wolfe, James P Bonaparte, David Schramm, Elizabeth Fitzpatrick, Vincent Lin, Becky Skidmore, David Moher, Brian Hutton

**Affiliations:** 1 Ottawa Hospital Research Institute, Ottawa, Canada; 2 Department of ENT, the Ottawa Hospital, Ottawa, Canada; 3 Dr. S. Kilty Medicine Prof. Corp, Ottawa, Canada; 4 Faculty of Health Sciences, University of Ottawa, Ottawa, Canada; 5 Children’s Hospital of Eastern Ontario Research Institute, Ottawa, Canada; 6 Department of Otolaryngology – Head & Neck Surgery, Sunnybrook Health Sciences Centre, Sunnybrook Research Institute, Toronto, Canada; 7 Faculty of Medicine, University of Toronto, Toronto, Canada; 8 University of Ottawa School of Epidemiology, Public Health and Preventive Medicine, Ottawa, Canada; University of Mississippi Medical Center, UNITED STATES

## Abstract

**Background:**

Hearing loss is one of the leading causes of disability worldwide. Patients with hearing loss experience impaired quality of life, as well as emotional and financial consequences that affect both themselves and their families. Idiopathic sudden sensorineural hearing loss (ISSNHL) is a common but difficult to treat condition that has a sudden onset of ≤ 72 hour associated with various etiologies, with the majority of cases being idiopathic. There exists a wide range of therapeutic options, however, the uncertainty surrounding their comparative efficacy and safety makes selection of treatment difficult. This systematic review and network meta-analysis (NMA) assessed the relative effects of competing treatments for management of ISSNHL.

**Methods:**

A protocol for this review was registered with PROSPERO (CRD42017073756). A detailed search of MEDLINE, Embase and the Cochrane Library from inception to February 8th, 2018 was carried out by an experienced information specialist. Grey literature was also searched. Screening full-text records, and risk of bias assessment were carried out independently by two reviewers, and disagreements were resolved through consensus or third party adjudication, while data was collected by one reviewer and verified by a second reviewer. Bayesian network meta-analyses (NMA) were performed to inform comparisons between interventions for a priori specified outcomes that included pure tone average (PTA) improvement and hearing recovery.

**Results:**

The search identified a total of 1,138 citations, of which 613 remained for review after removal of duplicates. Of these, 23 publications describing 19 unique studies (total sample size of 1,527) met our a priori eligibility criteria, that were assessed to be at unclear or high risk of bias on several domains. We identified data on several interventions for ISSNHL therapy and were able to construct treatment networks consisting of six intervention groups that included placebo; intratympanic (IT) steroid; IT plus systemic steroid; per oral (PO) steroid; intravenous (IV) steroid; and IV plus PO steroid for our NMAs. IT plus systemic steroids demonstrated the largest difference in PTA improvement compared to placebo (25.85 dB, 95% CrI 7.18–40.58), followed by IV plus PO steroids (22.06 dB, 95% CrI 1.24–39.17), IT steroids (18.24 dB, 95% CrI 3.00–29.81). We observed that the difference of PTA improvement between each intervention and placebo diminished over time, attributed to spontaneous recovery. The binary outcomes of hearing recovery demonstrated similar relative ordering of interventions but were less sensitive than PTA improvement to capture the significant differences between interventions and placebo.

**Conclusion:**

Unclear to high risk of bias trials rated IT plus systemic steroid treatment as the best among the six interventions compared, and all active treatments were better than placebo in improving PTA. However, it should be noted that certain comparisons were based on indirect evidence only or few studies of small sample size, and analyses were unable to control for steroid type and dosage. Given these limitations, further data originating from methodologically sound and rigorous trials with adequate reporting are needed to confirm our findings.

## Introduction

Hearing loss is one of the leading causes of disability worldwide[1]. This is also true in Canada, where one of every four Canadian adults has some form of hearing loss, and more than one million are reported to have hearing-related disability[2]. Canadians with hearing loss endure a severely impaired quality of life despite the availability of many interventions[3]. Idiopathic sudden sensorineural hearing loss (ISSNHL) is an acute disruptive disorder often occurring in the prime of mid-life. Although many treatment options have been introduced for ISSNHL during the past 80 years[4], the burden of disease remains significant. The emotional and financial toll on patients, their families, and society is large and often underestimated[5]. In cases of pre-existing hearing loss on the opposite side, the problem can be even more pronounced. ISSNHL is an otologic emergency in which early diagnosis and rapid treatment are advised[6]. However, the variety of etiologies of hearing loss and the uncertainty surrounding the efficacy of available interventions makes the selection of appropriate treatments challenging.

SSNHL, while a somewhat common form hearing loss, is difficult to treat and has major impacts on mental health and quality of life. The annual incidence of SSNHL in the United States is about 27 cases per 100,000 population [7]. SSNHL occurs rapidly over 72 hours or less (≤3 days)[8,9] and is often associated with other debilitating symptoms—including tinnitus, intractable vertigo, and hyperacusis—that result in extreme patient anxiety[10,11]. Spontaneous recovery is reported in 32% to 65% of cases, though only a small number of patients will restore hearing to functional levels[12–14]. Patients who fail to recover will endure the social isolation associated with the inability to understand speech, the inability to localize sound, and pervasive tinnitus leading to increased risks of anxiety disorder and depression[15–17]. There are several etiologies for SSNHL, with the majority of cases being idiopathic[10,18]. Various therapeutic options are available for ISSNHL including corticosteroids, anti-inflammatory agents, antiviral agents, diuretics, vasodilators, rheologic agents, hyperbaric oxygen and tri-iodobenzoic acid derivatives[8,18–22]. For instance, clinicians may offer corticosteroids as initial therapy to ISSNHL patients or hyperbaric oxygen therapy within 3 months of diagnosis of ISSNHL, as per the American Academy of Otolaryngology-Head and Neck Surgery’s clinical practice guideline (CPG)[8]. Based on the Otolaryngology Association of Madrid’s consensus statement, there exists no difference between antiviral agents and placebo, but oral corticosteroid (if early diagnosis <30 days), IV corticosteroid (if hearing loss>70db, single ear or with associated intense vertigo), and intratympanic corticosteroids (if no complete response to systemic steroids after 7 days) are recommended[19]. However, the optimal treatment for idiopathic SSNHL remains controversial due to the potential for spontaneous recovery in many patients[20]. Patients with no or inappropriate treatment and who do not recover spontaneously will experience lifelong social isolation and work-related difficulties associated with chronic single-sided deafness and tinnitus[17]. These difficulties could be exponential for individuals with pre-existing hearing loss in the contralateral ear. There is a need to establish the comparative benefits and harms of previously studied treatments, as well as to prioritize the available treatments and to identify considerations for future research. In scenarios wherein there is a need to compare more than two treatment strategies simultaneously and there is the potential that both relevant direct and indirect data exist, network meta-analysis (NMA) represents a continually evolving evidence synthesis methodology of great value and relevance[23–25]. We conducted a systematic review incorporating NMAs to assess the relative effects of competing treatments for management of ISSNHL in terms of hearing recovery and other key patient outcomes.

## Methods

Our published review protocol for this systematic review provides detailed information on the methodological approach to evidence searching and synthesis, eligibility criteria, study screening and selection, data extraction and quality assessment[26]. We incurred no deviations from the a priori review protocol, with the exception of a minor modification of our modeling approach for: 1) continuous endpoints at baseline and at final follow-up with corresponding standard deviations (but without average changes and corresponding standard deviations per group) in certain studies; and 2) follow-up time due to variations in endpoints assessment time across studies. Further details on these deviations are provided in the sections that follow below.

This review adheres to methods recommended by the Cochrane Collaboration for conduct of systematic reviews of interventions[27], and conforms to reporting standards of the Preferred Reporting Items for Systematic Reviews and Meta-Analyses for Network Meta-Analyses (PRISMA-NMA) [28]. This review was registered with the International Prospective Register of Systematic Reviews (PROSPERO)[29], registration number CRD42017073756. A summary of the study methodology is described next.

### Literature search strategy

An experienced information specialist developed the search strategies for the review in consultation with the research team. Using the OVID platform, we searched Ovid MEDLINE^®^, including Epub Ahead of Print and In-Process & Other Non-Indexed Citations, and Embase Classic+Embase. We also searched the Cochrane Library on Wiley. All searches were performed up to Feb 8^th^, 2018. Searches utilized a combination of controlled vocabulary (e.g., “Hearing Loss, Sensorineural”, “Hearing Loss, Unilateral”, “Hearing Loss, Sudden”) and keywords (e.g., sudden hearing loss, sudden deafness, SSNHL), and a randomized controlled trial (RCT) filter was applied in MEDLINE and Embase. Syntax was adjusted according to the needs of each database, and animal-only and opinion pieces were removed where possible. The search was peer reviewed prior to execution by a second information specialist using the PRESS Criteria[30], and appropriate recommendations were incorporated. We performed a separate search for systematic reviews to compare the listings of included studies from existing reviews against those retrieved from our core RCT searches. A targeted grey literature search of ClinicalTrials.gov and the International Clinical Trials Registry Platform (ICTRP) search portal was also undertaken to identify in-progress and completed trials. The full search strategy is presented in S1 Text.

### Identification of articles

Detailed eligibility criteria described in terms of the PICOS (Population-Intervention-Comparators-Outcomes-Study design) framework were reported in our published protocol[26] and are also provided in Table A in S2 Text. Briefly, to be included, studies had to be randomized controlled trials (RCTs) comparing available pharmacological interventions to placebo or other active arms for treating patients with ISSNHL defined as a 30 decibel (dB) hearing loss in three consecutive frequencies in one ear whose onset occurs in ≤3 days. We excluded other study designs, as well as RCTs that did not use the stated definition for diagnosis of ISSNHL.

### Screening and data collection process

We performed screening in two levels via two reviewers (NA and LE) working independently against the a priori eligibility criteria using the online systematic review software program Distiller Systematic Review (DSR); Evidence Partners Inc, Ottawa, Canada). Screening at Level 1 was based on review of titles and abstracts using the liberal accelerated method[31], where only one reviewer is required to include citations for further assessment at full-text screening and two reviewers are needed to exclude a citation. The overall Kappa for inter-reviewer agreement was 0.87. Level 2 screening encompassed review of full text articles by two reviewers (NA and LE) independently and in duplicate and the overall Kappa was 0.74. We commenced screening at both levels with a calibration exercise to ensure consistent application of eligibility criteria. Disagreements among reviewers were resolved through consensus or third-party adjudication. The included listings of identified review articles were inspected to confirm no relevant studies were missed. The process of study selection has been summarized using a flow diagram.

A standardized form implemented in Microsoft Excel (Microsoft Corporation, Seattle, Washington, USA) was used for data extraction, recording key items that included publication traits (e.g. author list, year and journal of publication), population characteristics (including eligibility criteria, diagnostic criteria and key patient demographics including initial hearing loss level, time delay from onset to treatment, baseline tinnitus, vertigo, and speech recognition score), details of the study intervention and comparators provided to patients, outcome information (including raw outcome data required for meta-analyses as well as author conclusions), and study design information (including methods for treatment assignment, blinding, duration of follow-up and other factors). Data extraction was performed by one reviewer (LE) and verification was carried out by a second senior reviewer (NA).

### Risk of bias assessment

We used the Cochrane Risk of Bias Tool for RCTs to evaluate the risk of bias of each included RCT[27]. The assessments were carried out by two reviewers (NA and LE) independently, and disagreements were resolved via consensus or third-party adjudication. The domains assessed include selection bias (sequence generation, and allocation sequence concealment), performance bias (blinding of participants and personnel), detection bias (blinding of outcome assessment), attrition bias (incomplete outcome data), reporting bias (selective reporting), and other biases (other source of bias). We also assessed the baseline imbalances between groups, considering severity of hearing loss and comorbidities that may predispose to hearing loss. Outcome specific risk of bias across all reported outcomes in a trial was assessed. If the risk of bias did not differ across the reported outcomes in a study, an overall rating was assigned. Such studies were assessed to be at an overall: 1) high risk of bias, if at least one of the domains was rated as high risk of bias; 2) unclear risk of bias, if none of the domains were rated as high risk and at least one of the domains was judged at unclear risk of bias; or 3) low risk of bias, if all domains were rated to be at low risk of bias. Findings from these assessments have been narratively summarized, with details provided in the appendices to the review.

### Statistical analyses

Network diagrams were generated to explore the evidence base available for each outcome of interest. Treatment nodes for each network were sized to proportionately reflect the numbers of patients randomized to each intervention included in the network, while the thickness of the edges joining the treatment nodes was sized to proportionately reflect the number of studies informing each treatment comparison. The validity of the transitivity assumption was assessed using established methods that included a detailed review of the patient and study characteristics amongst members of the review team[32].

We considered both PTA improvement and the degree of hearing recovery as continuous and the categorical/dichotomous outcomes, respectively. To maximize clinical relevance of the analyses while dealing with the presence of heterogeneity in the definition of the categorical outcome of hearing recovery amongst the set of included studies, we consulted with our team’s clinical experts and categorized hearing recovery in two separate dichotomous outcome measures (total recovery and responders’ recovery) for the purposes of data analyses. ‘Total recovery’ compared patients with an improvement of either (1) hearing threshold ≤ 25 dB in affected ear or (2) return to within 10 dB hearing loss (HL) of either unaffected ear or the affected ear before hearing loss (in one instance return to within 5–10% Word Recognition Score (WRS) of unaffected ear was also considered[33]), to the patients without such an improvement. ‘Responder recovery’ referred to patients with any improvement versus those with (1) <15 dB HL gain or (2) <10 dB PTA improvement or (3) <10% increase in WRS.

Comparisons of the effects of interventions for the continuous outcome of PTA improvement as well as the dichotomous outcome of hearing recovery were estimated using NMA. Both fixed effect (FE) and random effects (RE) models with a vague prior distribution were planned for treatment effects (specifically, Normal (0, 10,000) for the mean difference in PTA improvement and Normal (0, 100) for the log odds ratio of binary total recovery and responder’s recovery). For the between-study standard deviation parameter in random effects models, a vague prior distribution was used (specifically, Uniform (0, 20) for PTA improvement and Uniform (0, 5) for the binary outcomes).

For the dichotomous outcomes of hearing recovery, we used FE and RE models with a binomial likelihood and a logit link (as outlined in a well-established model[34]). For analyses of the continuous endpoint of PTA improvement (measured in dB), some of the included studies reported mean improvement from baseline and corresponding standard errors while others reported PTA at baseline and at final follow-up with corresponding standard deviations (but without average changes and corresponding standard deviations per group). For studies that reported findings using the latter approach, we calculated the mean changes from baseline and imputed the standard errors of the mean changes (detailed in S3 Text). Based on the mean PTA improvement and corresponding standard errors (whether reported in the study reports or imputed as described above), we modified established FE and RE models with Normal likelihood and identity link[34] to allow for the model to incorporate both formats of data.

The modelled PTA improvement for each intervention, as well as the difference of PTA improvement compared to placebo, were estimated along with 95% credible intervals (CrI). We also estimated several secondary measures of effect described previously[35], including surface under the cumulative ranking curve (SUCRA) values, mean treatment rankings (with 2.5% and 97.5% quantiles), and probability best values (i.e., p(best)) of interventions. SUCRA and p(best) values range between 0 and 1, with values nearer 1 indicative of preferred treatments. Smaller values of the mean rank also suggest preferred treatments. Rankograms were prepared to show the distribution of probabilities of each intervention being ranked at different ranking positions. Lastly, league tables were prepared to summarize effect sizes and the probabilities that one intervention is better than another for all pairwise comparisons related to each outcome analyzed using NMA.

Selection between models was based upon deviance information criteria (DIC), with a threshold of five points lower than the DIC of any other models suggesting optimal fit. Model convergence was assessed using established methods including Gelman-Rubin diagnostics and the Potential Scale Reduction Factor (Rhat). All NMAs were performed using OpenBUGS software[36] version 3.2.3 and the R2OpenBUGS package[37] version 3.2–3.2 in R. The OpenBUGS code for PTA improvement (based upon the TSD-2 random effects model for continuous outcomes, with modifications) is provided in S3 Text.

NMA models using the time of outcome assessment as a covariate were also performed as sensitivity analyses, assuming per oral (PO), intravenous (IV) and IV+PO steroids shared a common slope while intratympanic (IT) and IT + systemic steroids shared another common slope over time, respectively.

In cases where we did not identify sufficient data regarding an a priori outcome specified in our review protocol to conduct NMA, we have presented a narrative summary of findings.

## Results

### Extent of available literature

**Fig 1:**
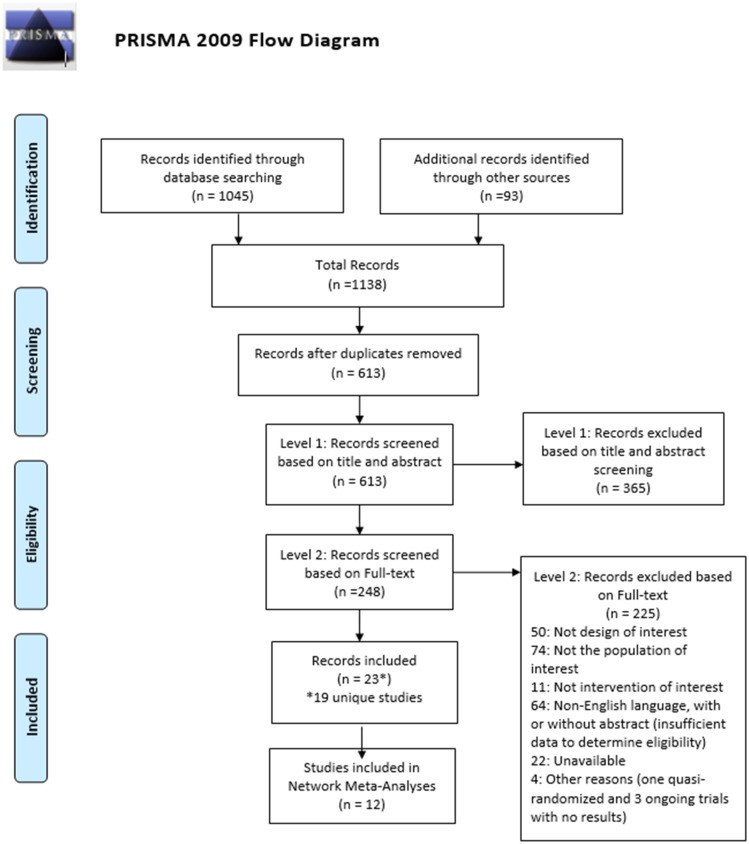
Process of study selection. The flow diagram shown presents details of the process of study selection toward identification of the evidence base for this systematic review.

We identified a total of 1,138 citations (database searches yield 1,045, grey literature 38, and reference checking 55) published from inception to February 8th, 2018. After removal of duplicates, we screened 613 bibliographic records at level 1 based on title and abstract. Of these, 365 were excluded and 248 records passed to level 2 for full text screening. A total of 225 records did not meet the eligibility criteria, and 23 records [4,12–14,33,38–55] describing 19 unique studies were included in the review. Of these, 12 unique studies were included in NMAs[4,12,14,33,38–45] (Fig 1). The remaining seven studies were not included in the NMAs for several reasons: two studies enrolled patients who had a considerably longer delay between the onset of ISSNHL and treatment[46,47] (i.e. years ranged from 7.6 to 8.2[47]; mean days ranged from 26.6 (SD 37.8) to 29.8 days (SD 40.9)[46]), which our clinical experts felt resulted in notable between-study differences in treatment approach that represented considerably clinical heterogeneity between study populations. In the remaining studies, time between the onset and treatment ranged from 2.69 days[33]to <1 month[43]. One study could not connect to a network given that no comparators were aligned with those from other studies[48], and one did not report outcome data[49]. Details on exclusion of additional studies are provided in the “Finding from NMAs” section of this report. Across all studies, the median publication year was 2012 (range 1998[13] to 2017[33,44]), while median sample size was 67 (range 30[46] to 278[48]). In terms of study funding, one study was funded by industry[49], six via non-industry sources[13,40,47,50,51,55], five had no funding[12,38,41,42,45], six did not report any funding[33,39,43,44,46,48], and one did not provide a clear statement on funding[14]. The studies were conducted in various countries and regions including Korea[12,40,49,50], Italy[14,38,51], Turkey[33,41], Iran[42], Taiwan[39], Sweden[55], Israel[48], Netherland[13], China[47], Thailand[46], India[45], Russia[43], and Greece[44].

### Study populations

A total of 16 studies (19 records)[12,14,33,39–53,55] were conducted in adult populations, while a mixture of adults and children were enrolled in three studies (four records)[13,38,46,54] (Table 1). All patients were diagnosed with ISSNHL, and those with known causes were excluded. The mean delay between onset of ISSNHL and time of treatment or study enrolment ranged from 3±1.9 days[4,52,55] to 10.1±8.1 days[49]. As noted earlier, we excluded two studies from NMAs in which the delay was longer (mean 7.6 to 8.2 years[47], and mean 26.6 to 29.8 years[46]). Baseline hearing measured by mean PTA in dB ranged from 37.1 (SD 16.67)[52] to 83.6 (SD 28.00)[13]. Eight studies reported baseline vertigo[4,13,14,33,42,44,45,50] and nine studies presented baseline tinnitus[4,13,14,33,41–43,45,50]; the proportion of patients with baseline vertigo ranged from 20.3%[48] to 91.4%[44], while the proportion of patients with baseline tinnitus ranged from 24%[56] to 91.4%[44]. Severity of hearing loss was measured by PTA in dB and was mainly categorized into four groups of mild (>20 to 40 dB, one study <50 dB), moderate (41 to 70 dB), severe (71–90 dB), and profound (>90 dB). Six studies[33,39,40,45,47,54] reported severity of hearing loss at baseline described as being either mild (proportion of patients ranged from 6%[40] to 66%[33]), moderate (proportion of patients ranged from 15.9%[40] to 29.6%[47]), severe (proportion of patients ranged from 30.3%[39] to 54.7%[45]) and profound (proportion of patients ranged from 25.92%[47] to 34.8%[40])]. A baseline hearing level measured between 0.25 and 8KHz frequencies was reported in five studies[38,40,42,43,51] (Table B in S2 Text). Table 1 and Table B in S2 Text provide detailed information on additional baseline demographic characteristics of patients, and Table C in S2 Text presents additional study characteristics of the included studies (including study eligibility criteria).

**Table 1:** Baseline demographic characteristics of the patients in the included studies based on treatment groups (1; 2; 3).

Author (Publication Year)	Treatment Groups	Sample	Age in years (M±SD)	Sex (F)	Affected site (Rt: Lt)	Initial PTA (M±SD)	Onset to treatment delay in days; (M±SD)	Tinnitus (n)	Vertigo (n)
**Hultcrantz (2014)**[4,52,55]	**Group 1**: Systemic Steroid (Oral Prednisolone: single dose capsules 60 mg daily for 3 days, thereafter reduced by 10 mg per day, for a total of at least 10 days)	47	56.8±12.7	23	22: 25	66.2±21.2	3±1.9	30	11
	**Group 2**: Placebo	46	53.8±13.5	17	24: 22	63.5±16.9	3.2±2.3	38	14
**Koo (2015)**[49]	**Group 1**: [IV EGb761 (Ginkgo biloba extract: 175 mg/ 50 mL in normal saline (500 mL) from day 1–5)+ methylprednisolone [orally for 14 days (48 mg for the first 7 days, and then tapered to 40 mg for the next 2 days, 16 mg for the next 2 days, and 8 mg for the final 3 days)]+ Oral EGb761(160 mg/day, twice daily from day 15–28)]	24	48.93±10.59	NR	NR	61.34±21.53 & 55.42± 16.09	3.52±1.96	NR	NR
	**Group 2**: (Placebo+ methylprednisolone**+ Oral EGb761**)	24	46.53±12.54	NR	NR	63.12±24.28 & 54.59±16.45	3.6±1.73	NR	NR
**Lim (2012)**[12,53]	**Group 1**: Intratympanic steroid (Dexamethasone: 5 mg/mL, 0.3–0.4 mL twice weekly for 2 weeks)	20	53.3±15.3	9	10: 10	58.9±31.2	10.1±8.1	NR	NR
	**Group 2**: Oral steroid (Prednisolone: 60 mg/d for 5 days, 40 mg/d for 2 days, 20 mg/d for 2 days, and 10 mg/d for 1 day, total 10 days)	20	51.3±14.5	10	8: 12	57.8±28.5	5.4±3.1	NR	NR
	**Group 3**: Intratympanic steroid** (Dexamethasone) +systemic steroid** (oral prednisolone)	20	47.8±14.2	10	9: 11	56.8±28.3	9.6±7.5	NR	NR
**Dispenza (2011)**[14]	**Group 1**: Intratympanic steroid (Dexamethasone: 4 mg/ml weekly for 4 weeks)	25	47±NR	Total: 18	Total: 20;26	65±NR	9.4±NR	19	NR
	**Group 2**: Oral steroid (Prednisolone: 60 mg of oral tapered over 14 days)	21	54±NR			51±NR	3.8±NR	17	NR
**Park (2011)**[50]	**Group 1**: Simultaneous IT-DEXA [Intratympanic dexamethasone (0.3 to 0.4 ml, 5 mg/mL, 3 times for two weeks) plus systemic steroid (IV dexamethasone 10 mg/d for 5 days and 7.5 mg/d for 2 days followed by oral prednisolone for 3 days in tapered doses after the patients were discharged from the hospital)]	44	45.36±12.36	25	NR	73.12±17.01	3.52±3.07	29	NR
	**Group 2**: Subsequent IT-DEXA [systemic steroid* (IV dexa followed by oral prednisolone**) followed by Intratympanic dexamethasone 7 days after beginning of systemic steroid for a total of 6 injects over 2 weeks]	44	48.05±10.83	11	NR	72.27±20.91	3±2.53	27	NR
**Filipo (2013)**[38]	**Group 1**: IT Prednisolone (0.3 ml of 62.5 mg/mL daily for 3 days) +/- oral prednisolone [(oral prednisolone (62.5 mg per day for 4 days, followed by 37.5 mg for 2 days, and 25 mg for the last 2 days) given if not recovered after 7 days of treatment]	25	§49.9±12.6	11	NR	53.7±9.25	7 (IT treatment)	NR	NR
	**Group 2**: Placebo +/- oral prednisolone** (oral prednisolone given if not recovered after 7 days of treatment)	25	50.8±14.7	9	NR	52.3±10	17 (oral treatment)	NR	NR
**Gordin (2002)**[48]	**Group 1**: Carbogen inhalation (95% O2, and 5% CO2 inhalation for a half-hour every 2 hours, daily for maximum 1 week) + intravenous MgSO4(4 g in 1000 ml of saline daily,14 drops/min, daily for maximum 1 week)	73	46.2±NR	Total: 81	NR	56.8±NR	4.4±NR	NR	14
	**Group 2**: Carbogen inhalation**	60	47.7± NR		NR	54.2±NR	4.7±NR	NR	13
**Yang (2010)**[39]	**Group 1**: Zinc (Zinga 78 mg, 10 mg elemental zinc; 2 tablets twice a day, 1 hour before breakfast and 1 hour before lunch for 2 months) +[IV Steroid (Dexamethasone 5 mg/6 hours for 2 days, followed by 5 mg/8 hours for 2 days, and then 5 mg/12 hours for 1 day) + Radiopaque contrast (diatrizoate sodium Hypaque 76, 10 ml/day for 5 days)+ Plasma expander (Dextran 40, 500 ml/day for 5 days)]	33	48.8±15.5	18	NR	78.98±22.93	4.8±NR	NR	NR
	**Group 2**: [IV Steroid (Dexamethasone**) + Radiopaque contrast** (diatrizoate sodium)+ Plasma expander** (Dextran 40)]	33	54.4±14.6	19	NR	76.06±25.96	5.2±NR	NR	NR
**Hong (2009)**[40]	**Group 1**: Intratympanic steroid (dexamethasone: 0.3 to 0.4 cc, 5 mg/mL once a day over the course of eight days)	32	56.9±NR	NR	14: 18	77.5±27.6	3.4±NR	NR	0
	**Group 2**: Oral steroid (prednisolone: 60 mg/d for 4 days, followed by 40 mg/d for 2 days, and 20 mg/d for 2 days) + other medications such as peripheral vasodilator, ginkgo biloba extract	31	56.2±NR	NR	13: 18	79.9±23.5	3.9±NR	NR	0
**Gundogan (2013)**[41]	**Group 1**: Combination therapy [intratympanic steroid (methylprednisolone: 0.4 mL of 62.5 mg/mL once every 3 days for 2 weeks) + oral steroid (methylprednisolone: 1 mg/kg and 10 mg taper every 3 days for 14 days)	37	52.32±12.94	NR	NR	80.7±22.81	4.7±4	9	NR
	**Group 2**: Oral steroid** (methylprednisolone)	36	51.6±16.77	NR	NR	76.3±27.18	5.14±3.52	9	NR
**Bianchin (2010)**[51]	**Group 1**: HELP apheresis (single selective apheresis session treated 3 L of plasma in 2 hours using a machine that monitors and controls fibrinogen/LDL apheresis)+ standard treatment [glycerol infusion (500 mL of glycerol, once a day for 10 days) + intramuscular dexamethasone (8 mg once a day for 10 days)]	72	52.8±NR	NR	37: 35	NR	12±NR	NR	NR
	**Group 2**: Standard treatment** (glycerol infusion + intramuscular dexamethasone)	60	60.4±NR	NR	30: 30	NR	13±NR	NR	NR
**Eftekharian (2015)**[42]	**Group 1**: Intravenous steroid (methylprednisolone: 500-mg daily for 3 consecutive days) + oral steroid (prednisolone: 1 mg/kg (maximum 60 mg) for 11 days), total 14 days treatment	29	42.2±12.6	NR	NR	76.07±25.6	6.7±2.2	21	18
	**Group 2**: Oral steroid** (prednisolone)	31	40.1±11.9	NR	NR	66.85±26.54	7.3±2.3	24	20
**Westerlaken (2003)**[13,54]	**Group 1**: IV acyclovir (10 mg/kg 3 times daily for 7 days) + IV prednisolone (1 mg/kg on day 1, to be diminished in equal steps to 0 mg over the course of 7 days)	37	§45.9±15.9	14	NR	62.9±21.6	*4.4±3.9	32	12
	**Group 2**: Placebo + IV Prednisolone**	33	44.7±17.6	10	NR	83.6±28.0	*4.2 ±3.4	24	12
**Hunchaisri (2015)**[46]	**Group 1**: Chelated zinc (75 mg, equivalent to 15 mg elemental zinc, one tablet three times after meal for one month) + standard treatment [oral prednisolone (60 mg/day in adults and 1 mg/kg/day in children, for seven days) + betahistine (12 mg)+ vitamin B1-6-12(three times a day for one month)]	16	§57.5±10.2	7	9: 7	68.1±25	26.6 ± 37.8	NR	NR
	**Group 2**: Standard treatment**(oral prednisolone + betahistine+ vitamin B1-6-12)	14	64.6±11.3	10	7: 7	56.1±19.7	29.8 ±40.9	NR	NR
**Wang (2012)**[47]	**Group 1**: Oral [Ginaton (extract of Ginkgo biloa leaves: tablets 80 mg/time and 3 times per day for 14 days) + prednisone (1 mg/kg (60 mg/day maximum) daily for 5 days followed by a 5-day taper (50, 40, 30, 20, and to 10 mg) for a total of 10 days of treatment) + mecobalamin (tablets 500μg/time and 3 times/day for 14 days)]	26	59.5(range 37–74)	10	16: 10	72 (range 36–107.1)	*8.2 years (range 0.5–19)	NR	NR
	**Group 2**: IV [Ginaton (105 mg every day for 14 days) + IV Dexamethasone (10 mg/day for 5 days followed by a dose of 5 mg/day for 5 day) + IV mecobalamin (500μg/ day for 14 days)]	28	57 (range 30–79)	12	15: 13	71.2 (range 35.8–110)	* 7.6 years (range 0.25–17)	NR	NR
**Kosyakov (2012)**[43]	**Group 1**: IT steroid (Dexamethasone: 4mg/cc every day for 10 days, 4 mg every other day over 20 days and then 4 mg 2 times a week over 5 months)	24†	49(IQR 35–52)	10	NR	41±12.87	<1 month	NR	4
	**Group 2**: IV steroid (Dexamethasone: 0.1 mg/kg in 200 ml of isotonic solution daily for 10 days) + [IV (Pentoxifylline+Cocarboxylase+Potassium and magnesium aspartate) +IM vitamin B-complex] for 10 days	24†	50(IQR 30–53)	9	NR	37.1±16.67	<1 month	NR	5
	**Group 3**: IV steroid (Dexamethasone: 0.1 mg/kg in 200 ml of isotonic solution, daily over 10 days)	25	40(IQR 32–53)	12	NR	39.1±16.97	<1 month	NR	6
**Swachia (2016)**[45]	**Group 1**: Oral steroid (prednisone: 1 mg/kg for the first 10 days. The drug was tapered to 0.5 mg/kg for the next 2 days and then 0.25 mg/kg for 2 days)	26	44.3(range 18–65) across arms	+	++	60.95±21.98	*<14 days	NR	8
	**Group 2**: IT steroid [methylprednisolone: one milliliter of the drug solution containing 40 mg of the drug (40 mg/Ml) twice a week for 2 weeks in a row)]	23		+	++	66.12±24.16	*<14 days	NR	5
**Tsounis (2017)**[44]	**Group 1**: IV steroid (prednisolone: 1 mg/kg daily for 7 days followed by 0.5 mg/kg daily for another 3 days)*** +PO steroid (methylprednisolone: 32 mg daily for 4 days followed by 16 mg daily for another 3 days)	35	50.1±17.3	15	18:17	NR	3.1±3.0	32	ˤ16
	**Group 2**: IT steroid (IT methylprednisolone: 0.4–0.6 ml of 62.5 mg/ml on the day of presentation, 3, 5 and 10 days after presentation, a total of 4 times)	34	53.2±12.0	16	20: 14	NR	4.6±3.0	31	ˤ13
	**Group 3**: Combination steroids (IV**+IT****+ Oral steroids**: methylprednisolone)	33	51.7±15.8	15	17: 16	NR	4.0±3.9	31	ˤ 8
**Ermutlu (2017)**[33]	**Group 1**: Oral steroid [prednisolone: 1 mg/kg (maximum 80 mg) and tapering 10 mg every 3 days]	16	41.06± NR	7	NR	NR	2.69±NR	13	6 across both arms
	**Group 2**: IT steroid (dexamethasone: 0.5–0.7 cc dexamethasone, 8 mg/2 ml three times every other day)	19	49.68± NR	5	NR	NR	3.74±NR	18	

*onset to study enrollment

** identical dosage/regimen that was administered for the comparator arm in the study

^§^studies that listed both adults and <18 years old as the population of interest. However, no information was provided on the actual number of included subjects that were <18 years old. The data were not reported separately for children population and there were no additional comments on the treatment effects in children in these studies. Westerlaken (2003)^50^ reported an age distribution ranging from 11–71 years, with the majority in their 40s and 50s; Hunchaisri (2015)[46] only stated inclusion of children when specifying the administered dose of the intervention; Filipo 2013^40^ reported age between 15–85 years as one of the inclusion criteria, but no further information was provided indicating whether any of the participants included were <18 years old.

*** After completing the IV course, patients were discharged and continued their treatment with oral steroid.

**** IT steroid was administered in combination with IV prednisolone on the day of presentation, 3, 5 and 10 days after presentation (total of 4 times). After completing this course, patients were discharged and continued their treatment with oral steroid.

^†^25 ears

^‡^male to female ratio was 1.6:1 across both arms

^‡‡^Left ear involvement was seen in 52.4% and right ear in 31.0% of cases. A total of seven patients (16.7%), four patients in group I and three patients in group II, had bilateral ear involvement.

^ˤ^ dizziness

Abbreviations: cc = cubic centimeter; DEXA = dexamethasone; F = female; g = gram; IT = intratympanic; IM = intramuscular; IV = intravenous; kg = kilogram; L = left; LDL = low-density lipoprotein; M = mean; ml = milliliter; n = number; NR = not reported; RT = right; SD = standard deviation

### Intervention and comparators

Of the 19 included studies, six compared IT steroid to systemic steroid (five via oral route[14,33,38,40,45], and one via IV route[43]); four assessed combination therapy (IT steroid plus oral steroid) against combination therapy (IT followed by systemic steroid[50], IV+ IT+ oral steroids[44]), IT steroid[44,53] or systemic steroid(oral)[41]; three evaluated systemic steroid (intravenous route) plus an additional intervention [PO steroid[42], ginkgo biloba extract+vitamin B12[47], zinc[39] or IV (Pentoxifylline+Cocarboxylase+Potassium and magnesium aspartate)+IM vitamin B-complex[43] to systemic steroid with or without additional interventions through PO or IV routes; two compared systemic steroid (PO steroid plus additional medication (zinc[46]; Acyclovir[13,54]) to PO steroid; one assessed carbogen plus MgSO4 versus carbogen[48]; and one evaluated apheresis plus standard treatment against standard treatment[44] (Table 1).

**Fig 2:**
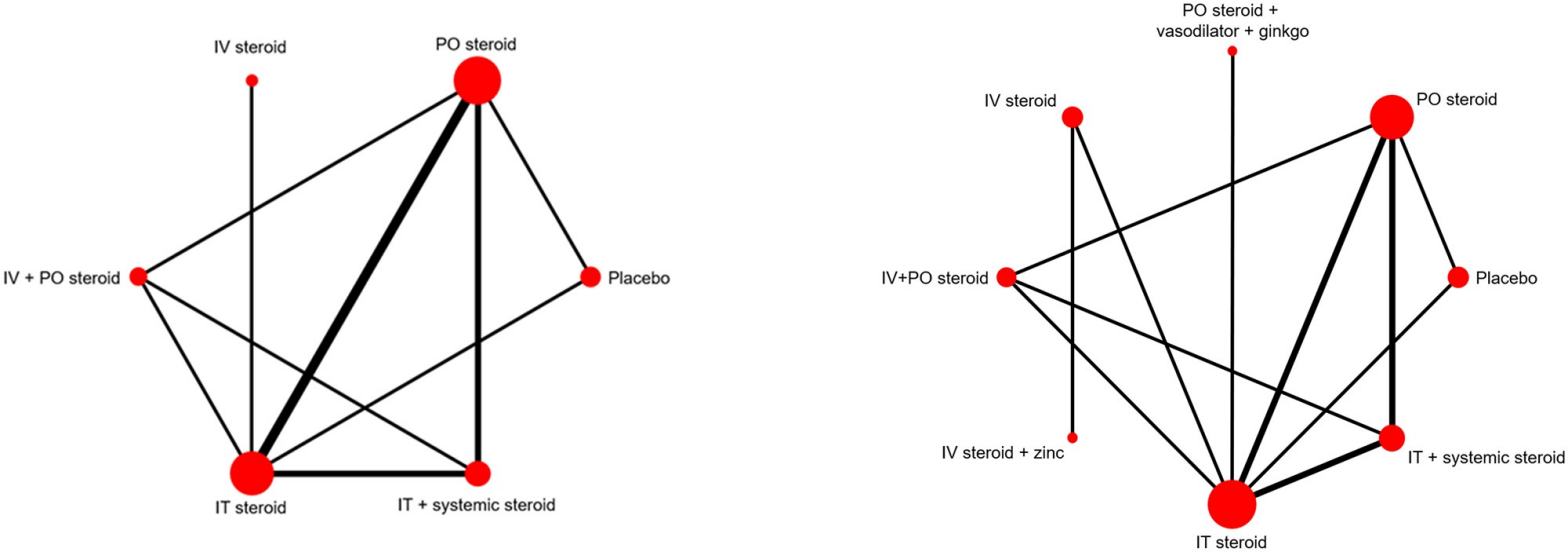
Network diagrams of PTA improvement without (left) and with (right) complementary medicine interventions. The size of treatment nodes was weighted by the number of patients, while the width of the edges each representing a pairwise comparison was weighted by the number of studies.

In consultation with clinical expertise from our research team, we classified the interventions into six treatment nodes to formulate a treatment network to compare treatment effects for PTA improvement; these groups consisted of placebo, IT steroid, IT plus systemic steroid, PO/IV steroid, PO steroid plus ginkgo biloba extract, and IV steroid plus zinc. Further details on the type of steroid, and number of arms of data involved in each treatment node are displayed in Fig 2 and **Table B** in S3 Text.

### Outcomes reported

We obtained data for six outcomes that included PTA improvement (measured as a continuous outcome)[12–14,38–46,50,51,54,55], hearing recovery (an ordinal categorical outcome where the criteria/categories differed between studies)[4,12–14,33,38–45,47,48,50,51,54,55], speech discrimination score (SDS) [13,38,41,45,42,50], vertigo[13,33,41,54], tinnitus[13,49], and harms[38,41,46,49,50]; based upon the availability of data and connectedness of treatment networks, we were able to conduct NMAs for two of these outcomes (PTA improvement and hearing recovery).

Outcome data were reported at various time points across studies ranging from 24 hours[51] to 12 months[13,38], and at multiple time points by certain studies. Based on input from our team’s clinical experts, the optimum window to measure the PTA improvement or total recovery was considered to be 60–90 days. We used available data measured closest to 60–90 days in our NMAs if data were reported at multiple time points. We also obtained data from single studies on hearing measures reported as speech reception threshold (SRT), air conduction threshold (ACT), and bone conduction threshold (BCT) for which meta-analyses were not feasible.

### Findings from risk of bias assessment

Twelve of the 19 included studies were assessed to have unclear risk of bias[4,14,39,40,41,44,46–48,52–54] and seven of the 19 were judged to be at high risk of bias[13,33,38,43,45,50,55] for all of the reported outcomes. Outcome-specific rating across all reported outcomes in a trial did not differ due to the subjective nature of the outcomes of interest. As such, the overall risk of bias rating of a trial pertains to reported outcomes. Table 2 displays the risk of bias status of the individual studies and Table D in S2 Text presents details of the assessments of risk of bias for each study. The most common reasons for overall assessments of unclear risk of bias were associated with the domains of selection bias and selective reporting bias; there was a lack of clarity as to whether the sequence generation and allocation concealment were carried out, and if all of the pre-specified outcomes were reported. Of the 19 included studies, only one[49] referenced a published protocol to cross check a priori outcomes against the reported ones. There was insufficient information regarding methods for sequence generation in nine trials[14,33,38–40,45,46,48,51], methods for allocation concealment in 13 studies[14,33,38–41,43,45–48,50,51], and for selective reporting in 16 studies[4,12–14,33,38–41,43–46,48,50,51]. Of the seven studies at high risk of bias, 3 were rated high risk for attrition[33,48,49], two for lack of blinding of outcome assessors[38, 45] and inappropriate random sequence generation[12,13], and one for lack of blinding of patients and personnel[43], lack of allocation concealment[12] and selective outcome reporting[49].

**Table 2:** Findings from risk of bias evaluation.

Study Author and Year	Adequate Sequence Generation	Allocation Concealment	Potential bias from lack of blinding of patients and personnel	Potential bias from lack of blinding of outcome assessors	Incomplete outcome data	Selective Reporting	Other bias
Hultcrantz 2014[4,52,55]	+	+	+	+	+	?	+
Koo 2015[49]	+	+	+	-	-	-	?
Lim 2012[12,53]	-	-	+	+	+	?	+
Dispenza 2011[14]	?	?	+	?	?	?	?
Park 2011[50]	+	?	+	+	+	?	+
Filipo 2013[38]	?	?	+	?	+	?	?
Gordin 2002[48]	?	?	?	?	-	?	?
Yang 2010[39]	?	?	-	+	+	?	?
Hong 2009[40]	?	?	+	+	?	?	+
Gundogan 2013[41]	+	?	+	-	?	?	+
Bianchin 2010[51]	?	?	+	+	+	?	+
Eftekharian 2015[42]	+	+	+	?	?	+	+
Westerlaken 2003[13,54]	-	+	+	+	?	?	?
Hunchaisri 2015[46]	?	?	?	?	?	?	?
Swachia 2016[45]	?	?	+	?	?	?	+
Kosyakov 2017[43]	?	?	?	?	?	+	?
Ermutlu 2017[33]	?	?	+	?	-	?	?
Tsounis 2017[44]	+	+	+	+	?	?	?
Wang, 2013[47]	+	?	+	+	+	?	?

Green cells containing ‘+’ symbols denote judgements of low risk of bias.

Yellow cells containing ‘?’ symbols denote judgements of unclear risk of bias.

Red cells containing ‘-’ symbols denote judgements of high risk of bias.

Full details for all assessments are provided in the review supplement.

### Findings from NMA

Network diagrams summarizing the evidence bases available for NMAs of PTA improvement and hearing recovery (both total recovery and responders’ recovery) are presented in Fig 2. Briefly, evaluations of model fit based on inspection of posterior total residual deviance and deviance information criteria consistently suggested for all three outcomes that RE models were better fits to the data (numeric details provided in **Table A** in S3 Text); findings from these models are thus the focus of results described next. Model fit measures suggested no presence of inconsistency.

### PTA improvement

A total of 14 studies (15 publications[12,14,38–45,50–52,54,55]) reported data, and 11 studies were included in the NMAs of PTA improvement that compared eight interventions[12,38–45,52,55]. Three studies were excluded from NMAs due to either insufficient data (reported no SDs for the pre- and post-treatment mean PTA[51]), presence of unbalanced baseline hearing loss between arms[54], or having compared the combined use of IT, IV and oral steroids in both arms.[50] Two studies assessed steroids with and without complementary medicine[39,40]; NMA findings including this intervention are provided in **Figures A-C** in S4 Text.

**Fig 3:**
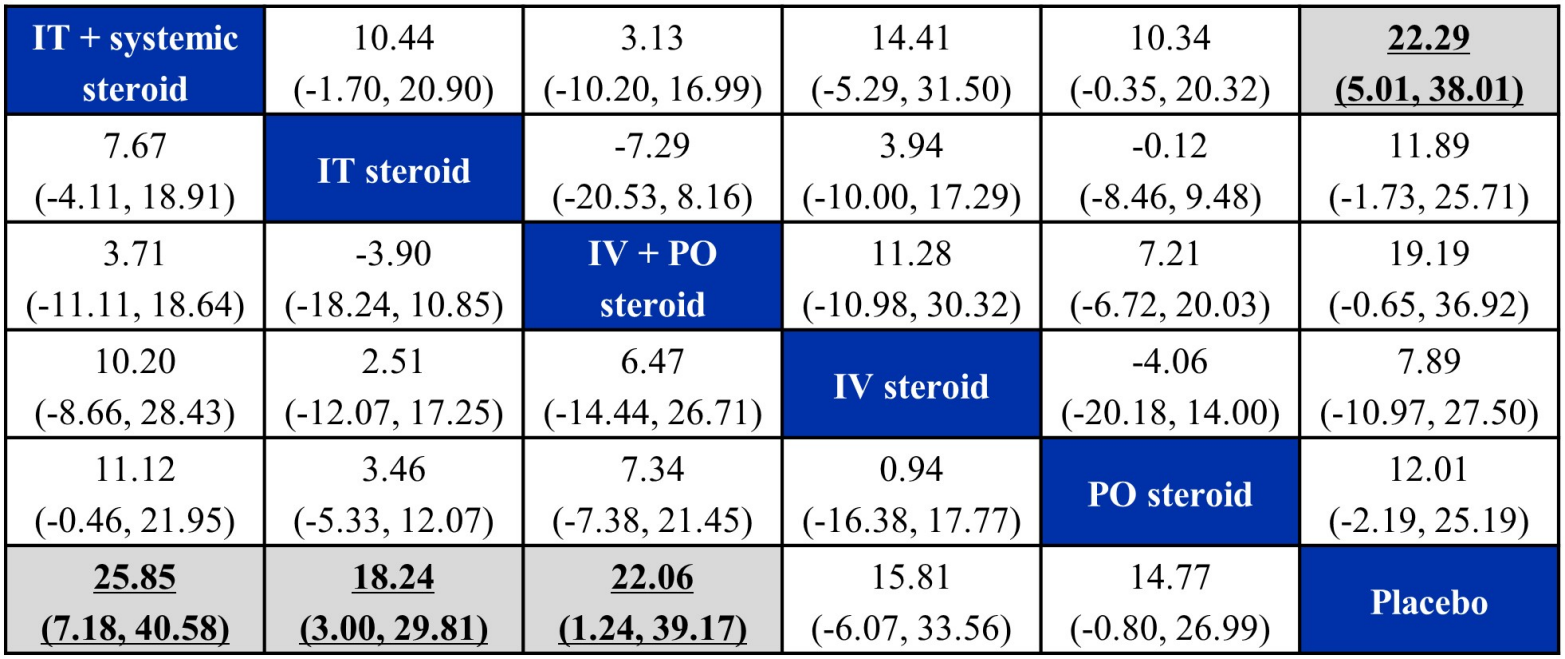
League table of pairwise difference estimates in PTA improvement. The league table of posterior median pairwise differences in PTA improvement from the unadjusted (lower triangle) and the time-adjusted models (estimated at the follow-up time of 60 days, upper triangle), with credible intervals (2.5% and 97.5% quantiles). A complete summary of estimates for efficacy from the RE consistency model assuming vague priors is displayed. Statistically significant differences in hearing recovery estimates between regimens are shown in bold, underlined font with shaded background. For each comparison, the lower/right-most treatment is the reference treatment. For example, the largest difference in PTA improvement compared to placebo was associated with IT plus systemic steroids, estimated as 22.29 dB (95% CrI 5.01–38.01) based on the time-adjusted model (at the follow-up time of 60 days).

Fig 3 presents a league table summary of mean differences estimated from NMA, while Table 3 presents a summary of secondary measures (including SUCRA, p(best) and treatment ranks). Based on summary estimates and corresponding 95% CrIs, IT plus systemic steroids, IT steroids, and IV plus PO steroids were each found to be better than placebo. The largest difference in PTA improvement compared to placebo was associated with IT plus systemic steroids: 25.85 dB (95% CrI 7.18–40.58; SUCRA 0.896, mean rank 1.52). In order of descending SUCRA value, the next best active interventions versus placebo were IV plus PO steroids (difference of 22.06 dB (95% CrI 1.24–39.17); SUCRA 0.723, mean rank 2.39), IT steroid (difference of 18.24 dB (95% CrI 3.00–29.81); SUCRA 0.568, mean rank 3.16), IV steroid (difference of 15.81 dB (95% CrI -6.07–33.56); SUCRA 0.434, mean rank 3.83), and PO steroid (difference of 14.77 dB (95% CrI -0.80–26.99); SUCRA 0.353, mean rank 4.23). All pairwise comparisons among active interventions were associated with credible intervals that failed to rule out the possibility of no difference (Fig 3).

**Table 3:** Mean SUCRA value, mean probability to be the best, and mean rank for each treatment based on PTA improvement, with the treatments in descending order of mean SUCRA. These secondary measures of effect from both unadjusted and time-adjusted (estimated at the follow-up time of 60 days) network meta-analyses are displayed. Larger values of the mean SUCRA or the smaller values of the mean rank suggest better treatments. SUCRA: the Surface Under the Cumulative RAnking curve (SUCRA) value represents the surface underneath the cumulative ranking curve, which is the posterior probabilities for each drug to be among the n-best options.

PTA improvement	Mean SUCRA	Mean Pr(best)	Mean Rank *
**NMA (unadjusted)**			
**IT + systemic steroid**	0.896	0.631	1.52 (1 to 4)
**IV + PO steroid**	0.723	0.255	2.39 (1 to 5)
**IT steroid**	0.568	0.028	3.16 (1 to 5)
**IV steroid**	0.434	0.079	3.83 (1 to 6)
**PO steroid**	0.353	0.006	4.23 (2 to 5)
**Placebo**	0.026	0.002	5.87 (4 to 6)
**Time-adjusted, at 60 days**			
**IT + systemic steroid**	0.914	0.658	1.43 (1 to 3)
**IV + PO steroid**	0.778	0.283	2.11 (1 to 5)
**IT steroid**	0.483	0.011	3.59 (2 to 5)
**PO steroid**	0.471	0.008	3.64 (2 to 5)
**IV steroid**	0.298	0.038	4.51 (1 to 6)
**Placebo**	0.055	0.003	5.72 (4 to 6)

* Mean rank with 2.5% and 97.5% quantiles in the parentheses.

After adjusting for time of outcome assessment, we observed that the difference of PTA improvement between each intervention and placebo diminished over time (**Figure A in** S3 Text), revealing a trend of spontaneous recovery. At the follow-up time of 60 days, the advantage of IT steroids and IV plus PO steroids over placebo was no longer present (Fig 3, **upper triangle and Figure A in** S3 Text). Additional results of the time-adjusted vs unadjusted NMA for PTA improvement are provided in **Figures A-D** in S3 Text.

### Findings, hearing recovery: Responder’s recovery and total recovery

A total of 15 studies (18 publications) reported data that related to 11 different interventions [4,12–14,33,38–45,50–52,54,55]. Of these, 11 studies were included in two NMAs of hearing recovery (11 for responder’s recovery and 10 for total recovery) that compared 7 interventions[4,12,14,33,38,40–45]. Four studies (five publications) were excluded for various reasons; two were due to the presence of an unclear outcome definition[51] (the criteria for patients with improvement versus with no change were unclear or there was inconsistency with our outcome definition for responders' recovery or total recovery[39]), one due to comparison between different routes of administering the same intervention (i.e., combined use of IT, IV and oral steroids in both arms)[50], and one (two publications) due to unbalanced baseline hearing loss levels between arms[13,54] (the study failed to control for severity of initial hearing loss despite double-blind randomization). One study compared IT steroid with PO steroid plus peripheral vasodilator and ginkgo biloba extract[40]. NMA findings from additional analyses including this intervention are provided in **Figures D-F** in S4 Text.

Pairwise odds ratios (OR) of the six treatment nodes and their corresponding credible intervals demonstrated that all treatments except for IV steroids were significantly better than placebo for responders’ recovery (**Figure E in** S3 Text). After adjustment for time of outcome assessment, the ORs summarizing comparisons of the treatments with placebo diminished over time; however, the advantage of all treatments, except for IV steroids, remained at 60 days (**Figure E in** S3 Text). The largest OR compared to placebo was associated with IT plus systemic steroid (16.10 (95% CrI 2.79–118.10; SUCRA 0.905, mean rank 1.47) and IV plus PO steroids (10.84 (95% CrI 1.65–89.48 versus placebo; SUCRA 0.670, mean rank 2.65).

Pairwise odds ratios (OR) of the six treatment nodes with corresponding credible intervals demonstrated that none of the treatments were different when compared with both placebo for total recovery (**Figure F in** S3 Text). After adjustment for time of outcome assessment, we observed all treatments except for IV steroids were associated with an advantage over placebo at the follow-up time of 30 days. However, the advantage diminished over time, and only IT plus systemic steroids retained an important advantage at 60 days (**Figure F in** S3 Text). The largest ORs compared to placebo were associated with IT plus systemic steroid (4.79, 95% CrI 1.01–23.45; SUCRA 0.836, mean rank 1.82) and IV plus PO steroids (4.89, 95% CrI 0.77–32.03 versus placebo; SUCRA 0.814, mean rank 1.93).

**Fig 4:**
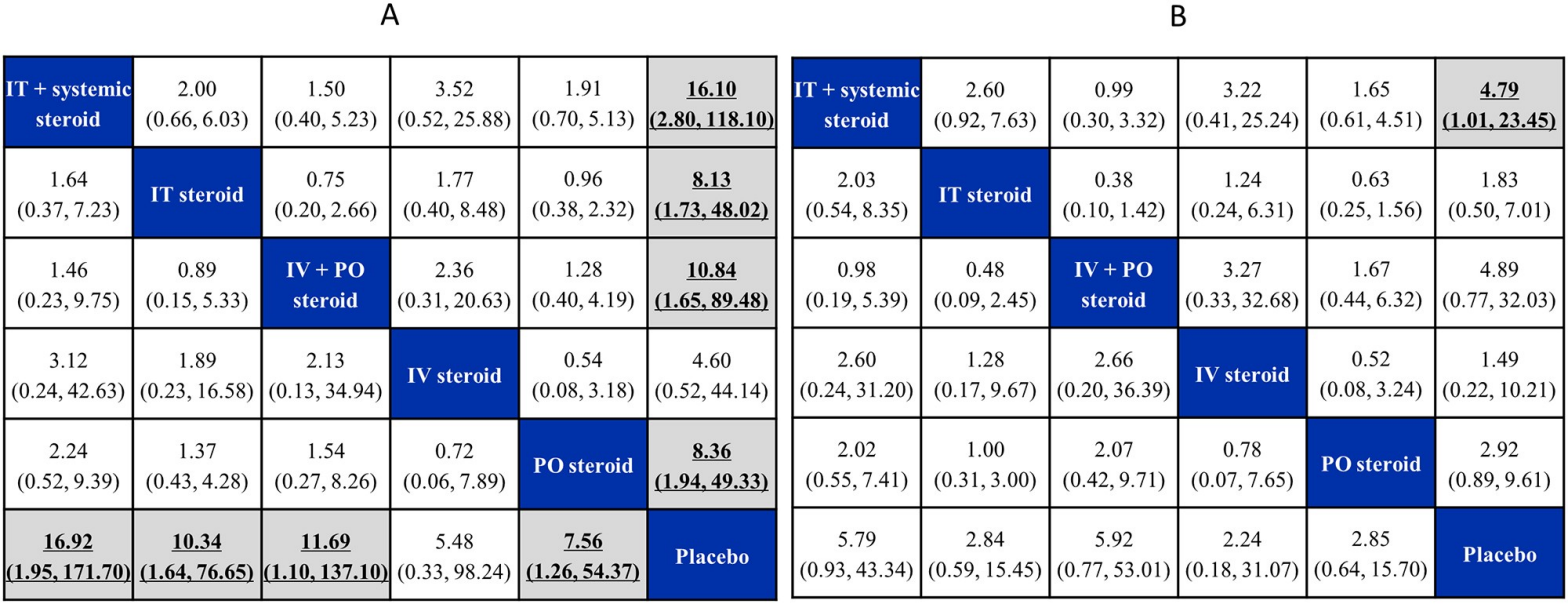
League tables of odds ratio estimates for responders’ recovery or total recovery. League tables of posterior median odds ratio in responders’ recovery / total recovery from the unadjusted (lower triangle) and the time-adjusted models (upper triangle), with credible intervals (2.5% and 97.5% quantiles). A complete summary of estimates for efficacy from the RE consistency model assuming vague priors is displayed. Statistically significant odds ratio estimates between regimens are shown in bold, underlined font. For each comparison, the lower/right-most treatment is the reference treatment. **Panel A**: responders’ recovery, **Panel B**: total recovery. For example, the largest OR compared to placebo was associated with IT plus systemic steroid, estimated as 16.10 (95% CrI 2.79–118.10) for responders’ recovery and 4.79 (95% CrI 1.01–23.45) for total recovery, based on the time-adjusted model (at the follow-up time of 60 days).

Details regarding the pairwise comparisons expressed as ORs with 95% CrIs are reported in Fig 4 (panels A and B), and the SUCRA value and mean rank for each treatment node are detailed in Tables 4 and 5 for responders’ recovery and total recovery. Additional results of the time-adjusted versus unadjusted NMA for both outcomes are provided in **Figures E-H** in S3 Text.

**Table 4:** Mean SUCRA value, mean probability to be the best, and mean rank for each treatment based on responders’ recovery, with the treatments in descending order of mean SUCRA. These secondary measures of effect from both unadjusted and time-adjusted (estimated at the follow-up time of 60 days) network meta-analyses are displayed. Larger values of the mean SUCRA or the smaller values of the mean rank suggest better treatments. SUCRA: the Surface Under the Cumulative RAnking curve (SUCRA) value represents the surface underneath the cumulative ranking curve, which is the posterior probabilities for each drug to be among the n-best options.

Responders’ Recovery	Mean SUCRA	Mean Pr(best)	Mean Rank *
**NMA (unadjusted)**			
**IT + systemic steroid**	0.835	0.528	1.82 (1 to 5)
**IV + PO steroid**	0.665	0.244	2.67 (1 to 5)
**IT steroid**	0.627	0.095	2.86 (1 to 5)
**PO steroid**	0.452	0.027	3.74 (1 to 5)
**IV steroid**	0.389	0.105	4.05 (1 to 6)
**Placebo**	0.031	0.002	5.85 (5 to 6)
**Time-adjusted, at 60 days**			
**IT + systemic steroid**	0.905	0.674	1.47 (1 to 4)
**IV + PO steroid**	0.670	0.204	2.65 (1 to 5)
**IT steroid**	0.553	0.041	3.24 (1 to 5)
**PO steroid**	0.528	0.029	3.36 (1 to 5)
**IV steroid**	0.338	0.052	4.31 (1 to 5)
**Placebo**	0.006	0.000	5.97 (5 to 6)

* Mean rank with 2.5% and 97.5% quantiles in the parentheses.

**Table 5:** Mean SUCRA value, mean probability to be the best, and mean rank for each treatment based on total recovery, with the treatments in descending order of mean SUCRA.

Total Recovery	Mean SUCRA	Mean Pr(best)	Mean Rank *
**NMA (unadjusted)**			
**IT + systemic steroid**	0.806	0.393	1.97 (1 to 5)
**IV + PO steroid**	0.793	0.435	2.04 (1 to 5)
**IT steroid**	0.461	0.023	3.69 (2 to 5)
**PO steroid**	0.460	0.025	3.70 (1 to 6)
**IV steroid**	0.389	0.118	4.06 (1 to 6)
**Placebo**	0.091	0.007	5.55 (3 to 6)
**Time-adjusted, at 60 days**			
**IT + systemic steroid**	0.836	0.406	1.82 (1 to 4)
**IV + PO steroid**	0.814	0.460	1.93 (1 to 5)
**PO steroid**	0.590	0.052	3.05 (1 to 5)
**IT steroid**	0.339	0.004	4.31 (3 to 6)
**IV steroid**	0.305	0.072	4.48 (1 to 6)
**Placebo**	0.116	0.007	5.42 (3 to 6)

* Mean rank with 2.5% and 97.5% quantiles in the parentheses.

### Other measures of effectiveness reviewed

For many of the a priori outcomes specified in our review protocol, the presence of insufficient and/or heterogeneous data precluded the performance of NMAs. A narrative summary of relevant findings for these outcomes was prepared and is presented next.

Regarding post-treatment Speech Discrimination Score (SDS) percentage, five studies (six publications) reported post treatment SDS percentage[13,38,41,42,45,50] at various time points. Two studies demonstrated statistically significant post-treatment SDS percentage differences between the intervention groups[41,49] at one month while one study did not[46]. In one study, the SDS percentage at day 28 post-treatment was statistically significant in favour of the intervention arm that received IV ginkgo biloba extract (EGb761) plus oral steroid plus oral ginkgo biloba extract (EGb761) compared to the control group that received placebo plus oral steroid plus oral EGb761 [Mean (SD) SDS percentage 87.48(28.65) versus 69.17(40.89), respectively; p = 0.050][49]. In the second study, SDS percentage at 4 weeks post-treatment was doubled in the intervention group receiving a combination of IT and oral steroid compared with oral steroid alone [Mean (SD) SDS percentage 41.08(21.98) versus 20.06(22.69), respectively; p<0.01][41]. In the third study, no statistically significant post-treatment difference was observed between patients receiving chelated zinc + standard treatment (oral prednisolone + betahistine+ vitamin B1-6-12) compared to standard treatment alone [Mean (SD) SDS percentage 57.0 (31.8) versus 74.9 (33.1), respectively; p = 0.144][46]. There were no pre-treatment SDS percentage differences between the intervention arms in the study (p = 0.614)[46]. Post-treatment SDS percentage improved in one study[13] at 3 months, but it did not in the other[42]. In one study the improvement reached 68% (between groups comparison was not reported)[13]. In the second study, there was no statistically significant difference between the treatment arms, IV steroid (methylprednisolone) + oral steroid (prednisolone) versus oral steroid (prednisolone), at 3 months post-treatment [Mean (SD) was 58.58(42.44) in IV steroid arm versus 63.06(41.14) in oral steroid arm, p = 0.680][42].

Regarding the occurrence of vertigo, four studies (five publications) reported short duration transient vertigo incidence[13,33,38,41,54]. In one study, 3/37 (8%) patients treated with IT steroids experienced vertigo versus none in patients who received oral steroid; the cases resolved within 2 hours[41]. In the second study, 6/50 (12%) patients experienced vertigo, however data were not separately reported for the intervention arms of IT steroids and IT placebo[38]. In the third study, 12.5% of patients treated with IV acyclovir + IV prednisolone exhibited vertigo versus 10.7% of patients in Placebo + IV prednisolone group[13]. In the fourth study, 21% (4/19) of patients in the IT dexamethasone group and none in the per oral prednisolone arm demonstrated vertigo.[33] Baseline vertigo results are presented in Table 1. One study reported that the proportion of patients with baseline vertigo decreased from 32.4% to 12.5% in the intervention arm receiving IV steroid plus IV Acyclovir, and from 36.4% to 10.7% in the comparator group that received IV steroid plus placebo at 12 months follow up[54].

Regarding tinnitus, two studies reported data on tinnitus improvement[13,49]. In the first study, baseline tinnitus measured by the tinnitus handicap inventory (THI; a self-administered questionnaire that scores from 0 to 100, with 100 referring to a catastrophic condition[57]) in four of 24 patients (16.6%) who received IV EGb761 (ginkgo biloba extract) + methylprednisolone + Oral EGb761 was improved (subjects were determined to be ‘improved’ if total scores had decreased), but in 19 it remained unchanged or aggravated (subjects were determined to be aggravated if total scores were increased) on day 28 post treatment[49]. Similarly, tinnitus was improved in 3/24 patients (12.5%) that received placebo + methylprednisolone + Oral EGb761, but remained unchanged or aggravated in 21/24 (87.5%) on day 28 post treatment[49]. The post-treatment THI did not differ between the two arms on day 28 [Mean (SD) was 20.33(27.39) in IV EGb761 group compared to 22.50(27.26) in placebo arm; p = 0.661][49]. In the second study, 86.5% of patients receiving IV acyclovir + IV prednisolone and 72.2% of patients in Placebo + IV prednisolone group had tinnitus. After 12 months, tinnitus decreased to 46.9% of patients in acyclovir group and 55.2% of patients in the placebo group[13].

Single studies reported data on hearing measures presented as SRT[49], BCT[49] and ACT that were reported at various timepoints[49]. Briefly, there was no statistically significant difference between the compared groups with regards to baseline and 28th day post-treatment SRT, BCT, and ACT. Detailed findings pertaining to these data are summarized in S2 Text.

### Measures of patient harm

Harms data were reported in very few of the included trials, and thus again no NMAs were feasible to compare data from all interventions. Data are narratively summarized next.

Regarding withdrawals due to harm, two studies reported relevant data. One study reported that one of 24 patients (4.1%) receiving treatment with IV EGb761 (ginkgo biloba extract)+ methylprednisolone+ Oral EGb761 withdrew from the study due to skin eruption[49]. Another study reported 3/30 patients (10%) receiving treatment with chelated zinc and/or standard treatment (oral prednisolone + betahistine+ vitamin B1-6-12) were excluded from the study due to intolerable nausea, vomiting, and vertigo[46].

Regarding tympanic membrane perforation after intratympanic injection of drug or placebo, two studies reported no cases of residual or persistent tympanic perforation (0/50 patients treated with either IT prednisolone +/- oral prednisolone, or placebo +/- oral prednisolone[38], and 0/37 patients treated with combination therapy [IT steroid (methylprednisolone) + oral steroid (methylprednisolone)])[42]. One study did not provide data per intervention group and it reported that small perforations were observed in 2/88 (2.3%) patients being treated with either simultaneous IT+ systemic steroid, or subsequent IT+ systemic steroid, and were closed either spontaneously or with the use of a paper patch[50].

Regarding the occurrence of other common side effects, ten studies (eleven publications) reported data on at least one adverse event such as nausea, vomiting, and headache[13,38,41,44–46,48–50,54,55]. Six studies stated that no side effects were observed[14,39,40,42,43,51], and three studies provided no description regarding the occurrence of common side effects[12,33,47].

### Subgroup analyses

Due to a lack of data, we were unable to carry out analyses for the a priori subgroups of interest outlined in our published protocol that included gender, age, prevalence of dizziness, patients’ prior history of steroid use, number of days since onset of hearing loss (or time to treatment), and severity of initial hearing loss.

### NMAs with complementary medicine interventions

Two studies assessed steroids with and without complementary medicine interventions (IV steroid with and without zinc[39], PO steroid with and without vasodilator and ginkgo[40]). Findings from NMAs with these studies and interventions included are summarized in **Figures A-F** in S4 Text. Based on the results obtained, IV steroid plus zinc may offer comparable benefits to those attained with IT + systemic steroids (Figures A-F in S4 Text). However, this finding should be interpreted cautiously given that the estimate was based on a single study[39] that connected IV steroids + zinc to the evidence network (through the grouping of IV steroids). A more recent study[46] found that PO steroid plus zinc had no significant effect on PTA or speech discrimination scores compared with PO steroid alone, though its long delay from onset to treatment (26.6 ± 37.8 and 29.8 ± 40.9 days in two arms) made it less clear whether the lack of difference could be associated with spontaneous recovery. The treatment effect of PO steroid plus vasodilator and ginkgo was similar to PO steroid alone in all three outcomes (Figures A-F in S4 Text).

## Discussion

To our knowledge, this is the first systematic review to compare pharmacological interventions for management of ISSNHL patients using network meta-analysis. Based upon the data identified from the set of included RCTs, NMAs were performed to assess the efficacy of six different interventions as initial therapy for ISSNHL. Outcomes of interest were hearing recovery as represented by improvement in PTA, total recovery and responder’s recovery. The results from our NMAs for PTA improvement suggest that IT + systemic steroids might be the best treatment among the interventions compared. While IT + systemic steroids demonstrated 95% credible intervals that did not rule out the possibility of a null difference when compared with other interventions, this therapy was associated with probabilities of 91.6%, 71.1%, 88.6%, and 97.1% to achieve greater PTA improvement compared to IT steroid alone, IV plus PO steroids, IV steroid alone, and PO steroid alone, based on the NMA model without time-adjustment, and 96.1%, 69.9% 94.5%, and 97.2% at 60 days if timing of assessment was accounted for (see Figure D in S3 Text). We observed that the difference of PTA improvement between each intervention and placebo (as well as the OR of responders’ recovery and total recovery) diminished over time, attributed to spontaneous recovery.

Park el al. (2011)^39^ compared IT steroid (as a salvage treatment) after 7 days of systemic steroids with simultaneous IT and systemic steroids in conjunction, which demonstrated no significant difference in hearing gain or earlier recovery rate. Among the 44 patients in the former group, 15 showed complete or partial recovery within 7 days of systemic treatment and did not receive IT dexamethasone treatment. Park and colleagues recommended the use of IT dexamethasone after 7 days of systemic steroid treatment to reduce the risk associated with unnecessary intratympanic injections. We present the NMA-based forest plots of its performance compared with other treatments in term of PTA improvement, responders’ recovery and total recovery in **Figures A-C** in S5 Text. Future trials which investigate systemic steroid(s) followed by IT steroid as a salvage therapy compared with simultaneous IT and systemic steroids or other interventions are needed.

Three prior systematic reviews assessed PTA improvement (decibels) using meta-analysis[58–60]. Han et al (2016) presented a meta-analysis of 12 trials comparing combined IT and systemic steroids versus systemic steroids, and reported a PTA change in dB favoring combination therapy (MD: 13.00, 95% CI: 9.24–16.77)[58]. Qiang et al (2016) conducted a systematic review that included six trials comparing IT steroid versus systemic steroids, and the authors reported a statistically significant difference favoring IT steroid for PTA change (MD: 3.42, 95% CI: 0.17–6.67)[59]. Lai et al (2017)[60] included six trials comparing IT steroids with systemic steroids. No significant difference in PTA improvement was observed between IT and systemic steroids (MD: 0.24, 95% CI: -2.43 to 2.91 for 5 studies with PTA assessed at 1–3 months; MD 4.69, 95% CI: -5.84 to 15.22 for 3 studies with PTA assessed at 6 months).

Three previous reviews assessed dichotomous outcomes of hearing recovery using meta-analysis. Sabbagh et al (2016)[61] reported findings from a systematic review of eight trials in which total recovery was defined as a return to within 10-dB hearing loss of the unaffected ear; the review compared IT dexamethasone to alternative treatment or placebo (five studies used oral steroid or combination, and two used IT placebo, and in one study, the authors used their institution’s standard treatment modality as control, consisting of a vasodilator, benzodiazepine, and vitamin B complex). The review failed to detect a statistically significant odds ratio between alternative treatment / placebo and IT dexamethasone (OR: 0.39, 95% CrI: 0.11–1.27)[61]. In two other reviews[58,60], the primary studies used various definitions of hearing improvement / recovery as defined by the authors of the included studies. Han et al. (2016) compared the proportion of patients with hearing improvement between combination of IT and systemic steroids versus systemic steroids alone based upon data from 13 trials[58]. Han et al. reported a statistically significant difference supporting combination therapy (OR: 2.50, 95% CI: 1.95–3.21)[58]. These significant findings might be attributed to pooling over more studies of incoherent definitions of binary outcome with a wide range of thresholds. Lai et al (2017)[60] showed similar hearing recovery rates between IT and systemic steroids (OR: 0.92, 95% CI: 0.59–1.43 for five studies with PTA assessed at 1–3 months; OR: 1.56, 95% CI: 0.52–4.68 for three studies with PTA assessed at 6 months).

In our review, hearing measured by PTA improvement and by the two binary outcomes responders’ recovery and total recovery demonstrated similar relative orderings of therapies, but slightly different properties of the outcomes were noticed: 1) PTA improvement was more sensitive to capturing differences between interventions and placebo than the binary outcomes of hearing recovery, which were less informative due to dichotomization; and 2) responders’ recovery was more sensitive to identification of differences between interventions and placebo than total recovery.

While a strength of this study is its novelty as the first NMA evaluating interventions for this condition, there are also several limitations that should be noted, several of which pertain to limitations of the included studies. We encountered numerous reporting limitations in several studies, including limited reporting of study design information relevant for risk of bias appraisals; many studies failed to provide sufficient information on how patient randomization was implemented, how sequence generation was performed, or if and how treatment allocation was concealed. We encourage authors to reference their trial protocols for transparency purposes. Poor reporting of study design features and methodology may affect internal validity of trials and their associated findings, thus representing a form of waste of resources (patients, funding and time). Second, there was a lack of clarity about reported effect estimates in several primary studies. For example, it was often unclear if the reported outcome for PTA improvement referred to the difference of PTA change between interventions, or only the difference in post-treatment values between interventions. To address this difficulty, we consulted our clinical experts and contacted the authors of the original trials to request clarity. We strongly encourage authors to carefully define their outcomes along with associated effect estimates. A third limitation of our review is the large between-study standard deviations that were estimated from our network meta-analyses, which may be a consequence of the diverse time of outcome assessment across studies. Analyses presented in S3 Text (see Figures A, E, and F) signified the huge impact of spontaneous recovery on the outcomes, however, adjusting for time of outcome assessment is only an expedient and ad-hoc solution in NMAs. We suggest future trials in this area consider (1) restricting the time of outcome assessment to between 30–90 days after initiation of treatment, (2) restricting the time from onset to treatment in order to reduce clinical heterogeneity between studies, and (3) reporting the mean difference of PTA change between study arms with confidence intervals, which will facilitate comparisons across studies. Fourth, due to a paucity of data, our analyses could not explore other aspects of treatment that were of a priori interest. This included considerations such as whether dosage and types of steroid (e.g. methylprednisolone, dexamethasone, and prednisone) were associated with different impacts on efficacy; and whether key subgroups related to gender, age, dizziness, patients’ history of steroid use, and severity of initial hearing loss impacted effectiveness. Lastly, it should be noted that the findings of our network meta-analyses should be interpreted with caution given that 7 of 19 included studies were assessed to be at high risk of bias and the remaining 12 were scored at unclear risk of bias, and there were few studies informing each treatment node.

### Conclusions

Unclear to high risk of bias trials rated IT plus systemic steroid treatment as the best among the compared six nodes. However, consideration of our findings may be limited by no studies or few studies with small sample size for some pairwise comparisons, not having accounted for the types of steroids and dosages, and lack of high quality RCTs. Given the stated limitations of the existing data, further research originating from methodologically sound and rigorous trials with adequate reporting is needed to confirm our findings.

## Supporting information

S1 Text PRISMA flow diagram of study selection. (DOCX)

S2 Text Tables of additional study information. (DOCX)

S3 Text Additional information regarding network meta-analyses. (DOCX)

S4 Text NMAs including complementary medicine interventions. (DOCX)

S5 Text NMAs including IT steroid (as a salvage treatment) after systemic steroids. (DOCX)

S6 Text PRISMA NMA checklist. (DOCX)

S1 Data Collected study details and outcomes. (XLSX)

## Abbreviations

MD: mean difference
NMA: network meta-analysis
PICO: population, intervention, comparator, outcome
PRESS: peer review of electronic search strategies
PTA: pure tone average
SDS: speech discrimination score
SR: systematic review
SRT: speech reception threshold
SSNHL: sudden sensorineural hearing loss
