# Supplementary material for: A systematic review and network meta-analysis of existing pharmacologic therapies in patients with idiopathic sudden sensorineural hearing loss
Source: PLoS One. 2019 Sep 9;14(9):e0221713. doi: 10.1371/journal.pone.0221713 (PMC6733451; doi:10.1371/journal.pone.0221713)
Supplement: S1 Text — (DOCX) [file pone.0221713.s001.docx]

# S1 Text: Search Strategy

RCTs

2017 Jul 27 (updated on Feb 8^th^, 2018)

Database: Embase Classic+Embase <1947 to 2017 July 26>, Ovid MEDLINE(R) Epub Ahead of Print, In-Process & Other Non-Indexed Citations, Ovid MEDLINE(R) Daily and Ovid MEDLINE(R) <1946 to Present>

Search Strategy:

--------------------------------------------------------------------------------

1 exp Hearing Loss, Sensorineural/ (50701)

2 Hearing Loss/ (37311)

3 Hearing Loss, Unilateral/ (1165)

4 Hearing Loss, Sudden/ (3944)

5 sudden*.tw,kw. (228447)

6 (1 or 2 or 3) and (4 or 5) (4384)

7 (sudden* adj3 ((loss* or lose or losing or lost) adj2 hear*)).tw,kw. (5134)

8 (sudden* adj3 ((degrad* or deteriorat*) adj2 hear*)).tw,kw. (37)

9 (sudden* adj3 (impair* adj2 hear*)).tw,kw. (69)

10 (sudden* adj3 deaf*).tw,kw. (3315)

11 (sudden* adj3 (hypac?usia* or hypacus#s or hypoac?usia* or hypoacus#s)).tw,kw. (35)

12 (SSHL or SSNHL or ISSHL or ISSNHL or SISHL or SISNHL).tw,kw. (1305)

13 (SSHLs or SSNHLs or ISSHLs or ISSNHLs or SISHLs or SISNHLs).tw,kw. (0)

14 or/4,6-13 (9034)

15 exp Animals/ not (exp Animals/ and Humans/) (15373598)

16 14 not 15 (7663)

17 (comment or editorial or interview or news).pt. (1750917)

18 (letter not (letter and randomized controlled trial)).pt. (1959538)

19 16 not (17 or 18) (7407)

20 (controlled clinical trial or randomized controlled trial or pragmatic clinical trial).pt. (559951)

21 clinical trials as topic.sh. (187587)

22 Randomized Controlled Trials as Topic/ (184839)

23 (randomi#ed or randomi#ation* or randomly or RCT$1 or placebo*).tw,kw. (1961467)

24 ((singl* or doubl* or trebl* or tripl*) adj (mask* or blind* or dumm*)).tw,kw. (367265)

25 trial.ti. (420874)

26 or/20-25 (2506883)

27 19 and 26 [RCTs] (481)

28 27 use ppez [MEDLINE RECORDS] (315)

29 exp perception deafness/ (26965)

30 hearing impairment/ (72258)

31 unilateral hearing loss/ (1668)

32 sudden deafness/ (4626)

33 sudden*.tw,kw. (228447)

34 (29 or 30 or 31) and (32 or 33) (3489)

35 (sudden* adj3 ((loss* or lose or losing or lost) adj2 hear*)).tw,kw. (5134)

36 (sudden* adj3 ((degrad* or deteriorat*) adj2 hear*)).tw,kw. (37)

37 (sudden* adj3 (impair* adj2 hear*)).tw,kw. (69)

38 (sudden* adj3 deaf*).tw,kw. (3315)

39 (sudden* adj3 (hypac?usia* or hypacus#s or hypoac?usia* or hypoacus#s)).tw,kw. (35)

40 (SSHL or SSNHL or ISSHL or ISSNHL or SISHL or SISNHL).tw,kw. (1305)

41 (SSHLs or SSNHLs or ISSHLs or ISSNHLs or SISHLs or SISNHLs).tw,kw. (0)

42 or/32,34-41 (9264)

43 exp animal experimentation/ or exp animal model/ or exp animal experiment/ or nonhuman/ or exp vertebrate/ (45967024)

44 exp human/ or exp human experimentation/ or exp human experiment/ (35976445)

45 43 not 44 (9992285)

46 42 not 45 (9021)

47 editorial.pt. (988355)

48 letter.pt. not (letter.pt. and randomized controlled trial/) (1954821)

49 46 not (47 or 48) (8708)

50 randomized controlled trial/ or controlled clinical trial/ (1190232)

51 exp "clinical trial (topic)"/ (246811)

52 (randomi#ed or randomi#ation* or randomly or RCT$1 or placebo*).tw,kw. (1961467)

53 ((singl* or doubl* or trebl* or tripl*) adj (mask* or blind* or dumm*)).tw,kw. (367265)

54 trial.ti. (420874)

55 or/50-54 (2679103)

56 49 and 55 [RCTs] (673)

57 56 use emczd [EMBASE RECORDS] (383)

58 28 or 57 [BOTH DATABASES] (698)

59 remove duplicates from 58 (449) [TOTAL UNIQUE RECORDS]

60 59 use ppez [MEDLINE UNIQUE RECORDS] (293)

61 59 use emczd [EMBASE UNIQUE RECORDS] (156)

***************************

Reviews

Database: Embase Classic+Embase <1947 to 2017 July 26>, Ovid MEDLINE(R) Epub Ahead of Print, In-Process & Other Non-Indexed Citations, Ovid MEDLINE(R) Daily and Ovid MEDLINE(R) <1946 to Present>

Search Strategy:

--------------------------------------------------------------------------------

1 exp Hearing Loss, Sensorineural/ (50701)

2 Hearing Loss/ (37311)

3 Hearing Loss, Unilateral/ (1165)

4 Hearing Loss, Sudden/ (3944)

5 sudden*.tw,kw. (228447)

6 (1 or 2 or 3) and (4 or 5) (4384)

7 (sudden* adj3 ((loss* or lose or losing or lost) adj2 hear*)).tw,kw. (5134)

8 (sudden* adj3 ((degrad* or deteriorat*) adj2 hear*)).tw,kw. (37)

9 (sudden* adj3 (impair* adj2 hear*)).tw,kw. (69)

10 (sudden* adj3 deaf*).tw,kw. (3315)

11 (sudden* adj3 (hypac?usia* or hypacus#s or hypoac?usia* or hypoacus#s)).tw,kw. (35)

12 (SSHL or SSNHL or ISSHL or ISSNHL or SISHL or SISNHL).tw,kw. (1305)

13 (SSHLs or SSNHLs or ISSHLs or ISSNHLs or SISHLs or SISNHLs).tw,kw. (0)

14 or/4,6-13 (9034)

15 exp Animals/ not (exp Animals/ and Humans/) (15373598)

16 14 not 15 (7663)

17 (comment or editorial or interview or news).pt. (1750917)

18 (letter not (letter and randomized controlled trial)).pt. (1959538)

19 16 not (17 or 18) (7407)

20 limit 19 to systematic reviews [Limit not valid in Embase; records were retained] (3780)

21 meta analysis.pt. (83476)

22 exp meta-analysis as topic/ (52519)

23 (meta-analy* or metanaly* or metaanaly* or met analy* or integrative research or integrative review* or integrative overview* or research integration or research overview* or collaborative review*).tw,kw. (279761)

24 (systematic review* or systematic overview* or evidence-based review* or evidence-based overview* or (evidence adj3 (review* or overview*)) or meta-review* or meta-overview* or meta-synthes* or "review of reviews" or technology assessment* or HTA or HTAs).tw,kw. (327945)

25 exp Technology assessment, biomedical/ (22459)

26 (cochrane or health technology assessment or evidence report).jw. (33642)

27 (network adj (MA or MAs)).tw,kw. (15)

28 (NMA or NMAs).tw,kw. (3634)

29 indirect comparison?.tw,kw. (3651)

30 (indirect treatment* adj1 comparison?).tw,kw. (478)

31 (mixed treatment* adj1 comparison?).tw,kw. (1150)

32 (multiple treatment* adj1 comparison?).tw,kw. (200)

33 (multi-treatment* adj1 comparison?).tw,kw. (3)

34 simultaneous comparison?.tw,kw. (907)

35 mixed comparison?.tw,kw. (35)

36 or/21-35 (590541)

37 19 and 36 (118)

38 20 or 37 [SYSTEMATIC REVIEWS] (3789)

39 38 use ppez [MEDLINE RECORDS] (122)

40 exp perception deafness/ (26965)

41 hearing impairment/ (72258)

42 unilateral hearing loss/ (1668)

43 sudden deafness/ (4626)

44 sudden*.tw,kw. (228447)

45 (40 or 41 or 42) and (43 or 44) (3489)

46 (sudden* adj3 ((loss* or lose or losing or lost) adj2 hear*)).tw,kw. (5134)

47 (sudden* adj3 ((degrad* or deteriorat*) adj2 hear*)).tw,kw. (37)

48 (sudden* adj3 (impair* adj2 hear*)).tw,kw. (69)

49 (sudden* adj3 deaf*).tw,kw. (3315)

50 (sudden* adj3 (hypac?usia* or hypacus#s or hypoac?usia* or hypoacus#s)).tw,kw. (35)

51 (SSHL or SSNHL or ISSHL or ISSNHL or SISHL or SISNHL).tw,kw. (1305)

52 (SSHLs or SSNHLs or ISSHLs or ISSNHLs or SISHLs or SISNHLs).tw,kw. (0)

53 or/43,45-52 (9264)

54 exp animal experimentation/ or exp animal model/ or exp animal experiment/ or nonhuman/ or exp vertebrate/ (45967024)

55 exp human/ or exp human experimentation/ or exp human experiment/ (35976445)

56 54 not 55 (9992285)

57 53 not 56 (9021)

58 editorial.pt. (988355)

59 letter.pt. not (letter.pt. and randomized controlled trial/) (1954821)

60 57 not (58 or 59) (8708)

61 meta-analysis/ (214776)

62 "systematic review"/ (145282)

63 "meta analysis (topic)"/ (36152)

64 (meta-analy* or metanaly* or metaanaly* or met analy* or integrative research or integrative review* or integrative overview* or research integration or research overview* or collaborative review*).tw,kw. (279761)

65 (systematic review* or systematic overview* or evidence-based review* or evidence-based overview* or (evidence adj3 (review* or overview*)) or meta-review* or meta-overview* or meta-synthes* or "review of reviews" or technology assessment* or HTA or HTAs).tw,kw. (327945)

66 biomedical technology assessment/ (21338)

67 (cochrane or health technology assessment or evidence report).jw. (33642)

68 (network adj (MA or MAs)).tw,kw. (15)

69 (NMA or NMAs).tw,kw. (3634)

70 indirect comparison?.tw,kw. (3651)

71 (indirect treatment* adj1 comparison?).tw,kw. (478)

72 (mixed treatment* adj1 comparison?).tw,kw. (1150)

73 (multiple treatment* adj1 comparison?).tw,kw. (200)

74 (multi-treatment* adj1 comparison?).tw,kw. (3)

75 simultaneous comparison?.tw,kw. (907)

76 mixed comparison?.tw,kw. (35)

77 or/61-76 (635691)

78 60 and 77 [SYSTEMATIC REVIEWS] (187)

79 78 use emczd [EMBASE RECORDS] (116)

80 39 or 79 [BOTH DATABASES] (238)

81 remove duplicates from 80 (167) [TOTAL UNIQUE RECORDS]

82 81 use ppez [MEDLINE UNIQUE RECORDS] (113)

83 81 use emczd [EMBASE UNIQUE RECORDS] (54)

Cochrane Library

Date Run: 27/07/17 18:56:22.577

ID Search Hits

#1 [mh "Hearing Loss, Sensorineural"] 403

#2 [mh ^"Hearing Loss"] 289

#3 [mh ^"Hearing Loss, Unilateral"] 16

#4 [mh "Hearing Loss, Sudden"] 138

#5 sudden*:ti,ab,kw 3521

#6 (#1 or #2 or #3) and (#4 or #5) 82

#7 (sudden* near/3 ((loss* or lose or losing or lost) near/2 hear*)):ti,ab,kw 318

#8 (sudden* near/3 ((degrad* or deteriorat*) near/2 hear*)):ti,ab,kw 0

#9 (sudden* near/3 (impair* near/2 hear*)):ti,ab,kw 2

#10 (sudden* near/3 deaf*):ti,ab,kw 175

#11 (sudden* near/3 (hypac*usia* or hypacus?s or hypoac*usia* or hypoacus?s)):ti,ab,kw 1

#12 (SSHL or SSNHL or ISSHL or ISSNHL or SISHL or SISNHL):ti,ab,kw 82

#13 (SSHLs or SSNHLs or ISSHLs or ISSNHLs or SISHLs or SISNHLs):ti,ab,kw 0

#14 {or #4, #6-#13} 409

DSR – 7 [Reviews]

DARE – 15 [Reviews]

CENTRAL – 380 [Trials]

HTA – 6 [Reviews]

NHS EED – 1 (*do not download*)

***************************
