# Supplementary material for: A systematic review and network meta-analysis of existing pharmacologic therapies in patients with idiopathic sudden sensorineural hearing loss
Source: PLoS One. 2019 Sep 9;14(9):e0221713. doi: 10.1371/journal.pone.0221713 (PMC6733451; doi:10.1371/journal.pone.0221713)
Supplement: S2 Text — (DOCX) [file pone.0221713.s002.docx]

# S2 Text: Tables of Additional Study Information

**Table A:** Review eligibility criteria (research question and its PICOS)

| Item | Description |
| --- | --- |
| **Research question** | To assess the relative effects of competing treatments for management of idiopathic SSNHL in terms of hearing recovery and other key patient outcomes. |
| **Population** | Adult patients with idiopathic single-sided SSNHL, defined as a 30 decibel hearing loss in three consecutive frequencies in one ear whose onset occurs in <3 days, with cause being disruption of the cochlea of the inner ear, the vestibular nerve or higher regions of auditory processing^6^. Cases of binaural and non-idiopathic single-sided SSNHL will not be included; such cases are usually associated with underlying conditions and should be managed in accordance. |
| **Intervention/**  **Comparators** | To maintain homogeneity across studies, the review focuses on first line therapy, excluding salvage therapy. The following interventions are of interest: *systemic steroids* (dexamethasone, hydrocortisone, betamethasone, prednisone, cortisone, prednisolone, methylprednisolone); *antivirals* (valacyclovir, acyclovir); *systemic volume expansion* (hydroxyethyl starch); *anti-thrombotics* (pentoxifylline, batroxobin, recombinant tissue plasminogen activator); *vasodilators* (prostaglandin E 1, naftidrofuryl); *increased tissue oxygenation therapies* (carbogen); *hyperbaric oxygen therapy;* *intratympanic steroid-combination therapies* (intratympanic methylprednisolone, intratympanic dexamethasone); *anti-inflammatory therapies* (Dextran 40, Dextran 40 + procaine hydrochloride); *anti-platelet vasodilatation* (prostacyclin, pentoxifylline); *other therapies* [magnesium aspartate, magnesium sulphate, fibrinogen/LDL apheresis, mannitol, nifedipine, fludiazepam, diazepam, hyperbaric oxygen, vitamin A, vitamin E, zinc, Chinese herbal medicine, Ginkgo biloba extract, AM-111 (a c-Jun N-terminal Kinase (JNK) ligand), Ozone therapy (auto-haemotherapy)]. |
| **Outcomes** | Endpoints of interest include hearing measures assessed via audiometric tests [i.e., pure tone audiometry, speech recognition scores (including word recognition and speech discrimination scores, and speech reception threshold (SRT)], and via clinical tests (i.e., tuning fork by Rinner test, and Weber test) where available; extent of recovery (e.g., Sieigel’s Criteria, or other such endpoint measures that categorize patients’ recovery as complete, marked, slight, or none based on decibels of improved hearing); quality of life (generic and disease-specific measures); reduction of tinnitus [could be measured via different techniques such as psychoacoustic tests of tinnitus (e.g., pitch match, loudness match, maskability, residual inhibition), rating scales (e.g., verbal rating scale, numerical rating scale, visual analog scale, poster style, mechanical device, etc.), questionnaires describing functional effects (e.g., tinnitus questionnaire, tinnitus handicap questionnaire, tinnitus severity scale, subjective tinnitus severity scale/tinnitus reaction questionnaire, tinnitus severity grading, tinnitus severity index, tinnitus handicap inventory, intake interview for tinnitus retraining therapy), and patients’ global perception of treatment-related changes]; incidence of vestibular endpoints (e.g., vertigo); harms (e.g., withdrawals due to adverse effects, otitis media, residual tympanic membrane perforation). |
| **Study Design** | Randomized controlled trials of any duration will be included. |

**Table B:** Baseline hearing level and hearing loss in the included studies according to intervention groups

| **Mean (SD) hearing level in frequencies (0.25-8 KHz):** | | | | | | | | | | | | | |
| --- | --- | --- | --- | --- | --- | --- | --- | --- | --- | --- | --- | --- | --- |
| **Author (Publication Year)**  **Treatment Groups** | **0.25 KHz** | **0.5 KHz** | **1 KHz** | | **2 KHz** | | **3 KHz** | | **4 KHz** | | **8 KHz** | | **Average across range of frequencies/Overall Mean (SD) PTA** |
| **Hong (2009)**^44^  **Group 1:** ITdexamethasone  **Group 2:** Oral prednisolone + other medications such as peripheral vasodilator, ginkgo biloba extract | G1:  63.07 (20.00)  G2:  68.00 (20.00) | G1: 74.10 (20.00)  G2: 80.00 (19.74) | G1: 81.53 (20.26)  G2: 83.33 (20.00) | | G1: 76.41 (20.00)  G2: 78.46 (20.00) | | G1: 77.94 (20.26)  G2: 78.71 (19.49) | | G1: 75.89 (20.26)  G2: 78.71 (19.49) | | G1: 76.92 (19.74)  G2: 83.33 (19.49) | | G1: 77.5 (27.6)  G2: 79.9 (23.5) |
| **Bianchin (2010)**^46^  **Group 1:** HELP apheresis + standard treatment (glycerol infusion + IM dexamethasone)  **Group 2:** Standard treatment (glycerol infusion + IM dexamethasone) | G1:  34.5 (NR)  G2:  46.8 (NR) | G1: 37.5 (NR)  G2: 46.0 (NR) | G1: 40.1 (NR)  G2: 41.9 (NR) | | G1: 40.7 (NR)  G2: 38.4 (NR) | | NR  NR | | NR  NR | | NR  NR | | NR  NR |
| **Eftekharian (2015)**^47^  **Group 1:**  IV methylprednisolone + oral prednisolone  **Group 2:** Oral prednisolone | NR  NR | G1: 65.51 (34.26)  G2: 62.50 (29.77) | G1: 72.24 (32.83)  G2: 63.87 (30.73) | | G1: 80.34 (25.14)  G2: 66.12 (28.71) | | G1: 86.20 (23.66)  G2: 74.83 (25.28) | | G1: 87.24 (23.91)  G2: 77.74 (24.62) | | NR  NR | | G1:76.07(25.6)  G2:66.85(26.54) |
| **Filipo (2013)**^40^  **Group 1:** IT Prednisolone +/- oral prednisolone (oral prednisolone given if not recovered after 7 days of treatment)  **Group 2:** Placebo +/- oral prednisolone (oral prednisolone given if not recovered after 7 days of treatment) | G1:  54.90 (13.60)  G2: 51.71 (12.07) | G1: 55.94 (8.99)  G2: 50.81 (10.81) | G1: 51.21 (11.32)  G2: 50.81 (12.79) | | G1: 51.12 (11.01)  G2: 50.63 (51.00) | | NR  NR | | G1: 53.64 (10.22)  G2: 53.15 (9.73) | | G1: 53.22 (12.59)  G2: 56.39 (13.52) | | G1: 53.7 (9.25)  G2: 52.3(10)  NR |
| **Kosyakov (2012)**^52^  **Group 1**: IT dexamethasone  **Group 2**: IV dexamethasone + IV (Pentoxifylline, Cocarboxylase, Potassium and magnesium aspartate) + IM vitamin B-complex **Group 3**: IV dexamethasone | G1: 35.1 (19.6)  G2:  32.9 (22.0)  G3:  33.1 (21.3) |  | G1:  38.5 (16.1)  G2:  35.2 (17.0)  G3:  38.4 (22.0) | |  | | NR  NR  NR | | G1: 50.1 (15.0)  G2:  44.3 (18.4)  G3:  45.8 (18.5) | | | | G1:  41.0 (12.9)  G2:  37.1 (16.7)  G3:  39.1 (17.0) |
| **Koo(2016)**^38^  **G1:** Ginkgo biloba extract  **G2:** Placebo | NR |  | NR | |  | | NR | | NR | | | | ACT:  G1: 61.34(21.53)  G2: 63.12(24.28)  BCT:  G1: 55.42(16.09)  G2: 54.59(16.45) |
| **Tsounis (2017)**^53^  **Group 1**: IV prednisolone + PO methylprednisolone  **Group 2**: IT methylprednisolone **Group 3**: IT methylprednisolone + IV prednisolone + PO methylprednisolone | NR |  | NR | |  | | NR | | NR | | | | G1: 81.1 (28.8)  G2: 81.4 (23.3)  G3: 79.1 (25.1) |
| **Gundogan (2013)**^45^  **Group 1:** IT steroid (methylprednisolone) + Systemic steroid (oral methylprednisolone)  **Group 2:** Systemic steroid (oral methylprednisolone) | NR |  | NR | |  | | NR | | NR | | | | G1: 80.6(22.82)  G2: 76.3(27.18) |
| **Lim (2012)**^12,55^  **Group 1**: Systemic steroid (oral Prednisolone)  **Group 2:** Intratympanic steroid (Dexamethasone)  **Group 3:**  Intratympanic steroid (Dexamethasone) +systemic steroid (oral prednisolone) | NR |  | NR | |  | | NR | | NR | | | | G1: 57.8(28.5)  G2: 58.9(31.2)  G3: 56.8(28.3) |
| **Dispenza (2011)**^14^  **Group 1:** Intratympanic steroid (Dexamethasone)  **Group 2:** Oral steroid (Prednisolone) | NR |  | NR | |  | | NR | | NR | | | | G1: 65(NR)  G2: 51(NR) |
| **Gordin (2002)**^42^  **Group 1:** Carbogen inhalation + intravenous MgSO4  **Group 2:** Carbogen inhalation | NR |  | NR | |  | | NR | | NR | | | | G1: 56.8(NR)  G2: 54.2(NR) |
| **Westerlaken (2003)**^13^  **Group 1:** IV acyclovir + IV prednisolone  **Group 2:** Placebo + IV Prednisolone |  |  |  | |  | |  | |  | | | | G1: 62.9 (21.6)  G2: 83.6(28) |
| **Hultcrantz(2014)**^4^  **Group 1:** Systemic Steroid (Oral Prednisolone)  **Group 2**: Placebo | NR |  | NR | |  | | NR | | NR | | | | G1: 67.4(20.9)  G2: 64(18.1) |
| **Hunchaisri (2015)**^41^  **Group 1:** Chelated zinc + standard treatment (oral prednisolone + betahistine+ vitamin B1-6-12)  **Group 2:** Standard treatment (oral prednisolone + betahistine+ vitamin B1-6-12) | NR |  | NR | |  | | NR | | NR | | | | G1:68.1(25)  G2:56.1(19,7) |
| **Park (2011)**^39^  **Group 1:** Simultaneous IT-DEXA [Intratympanic dexamethasone plus systemic steroid (IV dexamethasone followed by oral prednisolone)]  **Group 2:** Subsequent IT-DEXA [systemic steroid (IV dexa followed by oral prednisolone) followed by Intratympanic dexamethasone] | NR |  | NR | |  | | NR | | NR | | | | G1: 73.12 (17.01)  G2: 72.27(20.91) |
|  | **Number of patients with hearing loss degree (initial PTA) of:** | | | | | | | | | | |  | |
| **Author (Publication Year)**  **Treatment Groups** | | | | **Mild (> 20 to 40 dB)** | | **Moderate (41-70dB)** | | **Moderate to Severe** | | **Severe (71- 90 dB)** | | **Profound (> 90 dB)** | |
| **Hong (2009)**^44^  **Group 1:** IT steroid (dexamethasone)  **Group 2:** Oral steroid (prednisolone)+ other medications such as peripheral vasodilator, ginkgo biloba extract | | | | G1: 2  G2: 2 | | G1: 6  G2: 4 | | G1: 4  G2: 5 | | G1: 10  G2: 10 | | NR  NR | |
| **Yang (2010)**^43^  **Group 1:** Zinc +[IV Steroid (Dexamethasone)+ Radiopaque contrast (diatrizoate sodium)+ Plasma expander (Dextran 40)]  **Group 2:** [IV Steroid (Dexamethasone)+ Radiopaque contrast (diatrizoate sodium)+ Plasma expander (Dextran 40)] | | | | G1: 0  G2: 6 | | G1: 10  G2: 17 | | NR  NR | | G1: 12  G2: 8 | | G1: 11  G2: 12 | |
| **Wang (2012)**^48^  **Group 1:** Oral [Ginaton (extract of Ginkgo biloa leaves) + prednisone + mecobalamin]  **Group 2:** IV [Ginaton + IV Dexamethasone + IV mecobalamin] | | | | G1: 2  G2: 2 | | G1: 6  G2: 10 | | NR  NR | | G1: 12  G2: 8 | | G1: 6  G2: 8 | |
| **Swachia (2016)**^54^  **Group 1:** Oral steroid (prednisone)  **Group 2**: IT steroid (methylprednisolone) | | | | G1 & G2: 9 | | G1 & G2: 10 | | NR  NR | | G1 & G2: 11 | | G1 & G2: 12 | |
| **Ermutlu (2017)**^33^  **Group 1:** Oral steroid (prednisolone)  **Group 2**: IT steroid (dexamethasone) | | | | Mild hearing loss (≤ 50 dB)  G1: 11  G2:12  Severe hearing loss (≥50 dB)  G1:5  G2:7 | | | | | | | | | |

Abbreviations: ACT=air conduction threshold; BCT=bone conduction threshold; dB: decibels; G1=group1; G2=group 2; IM= intramuscular; IT=intratympanic; IV=intravenous

**Table C**: Study characteristics of the included studies in SSNHL network meta-analysis and systematic review

| **Author (Publication Year)** | **Inclusion criteria**  **(underlined text refers to ISSNHL definition);**  **Intervention groups** | **Mean (SD) Days between onset and treatment;**  **Quality Assessment (QA); Time of outcome assessment*** |
| --- | --- | --- |
| **Hultcrantz (2014)**^4^, companion papers: Nosrati-Zarenoe (2012)^40^, Nosrati-Zarenoe (2011)^38^ | Patients aged 18 to 80 years, referred by general practitioners or seeking care directly, presenting with sudden onset of hearing loss developing within 24 hours, and without any known cause (no earlier or present ear diseases). The average change in hearing threshold should be 30 dB or higher for the 3 most affected contiguous frequencies in the affected ear. Enrolment and treatment were to be started within 7 days from onset.  **Group 1:** Systemic Steroid (Oral Prednisolone)  **Group 2**: Placebo  **Details of treatment protocol**: Prednisolone as 10 mg capsules given as a single dose of 60 mg daily for 3 days, thereafter reduced by 10 mg per day, with a total treatment time of eight days. If recovery was complete treatment stopped; otherwise, medication was continued at 10 mg daily to a total of 30 days from beginning. Similar protocol was carried out for placebo arm. | **Group 1**: 3.0 (1.9)  **Group 2:** 3.2 (2.3)  **QA:** Unclear Risk of Bias  **Follow up time used in the analyses**: 90 days |
| **Koo (2015)**^38^ | Patients aged 20–70 years, ISSNHL, no history of other treatments for hearing loss, and a visit to the hospital within 1 week from the day of symptom onset. ISSNHL was diagnosed by the sudden onset SNHL with 30 dB and more decrease of hearing threshold in at least consecutive three frequencies.  **Group 1:** [IV EGb761 (Ginkgo bilbo extract) + methylprednisolone+ Oral EGb761]  **Group 2:** (Placebo+ methylprednisolone+ Oral EGb761)  **Details of treatment protocol**: Methylprednisolone was administered orally for 14 days (48 mg for the first 7 days, and then tapered to 40 mg for the next 2 days, 16 mg for the next 2 days, and 8 mg for the final 3 days). From day 1 (first day of administration) today 5, EGb761 or normal saline was administered once daily by intravenous infusion. In the EGb761 group, EGb761 (Tanamin ) 175 mg/ 50 mL in normal saline (500 mL) was administered; normal saline (550 mL) was used in the placebo group. Oral EGb761 (Tanamin) tablets (160 mg/day, twice daily) were administered in both groups from day 15 to day 28. | **Group 1:** 3.52 (1.96)  **Group 2**: 3.60 (1.73)  **QA:** High Risk of Bias  **Follow up time used in the analyses**: NA (the study didn’t report the outcomes in the network meta-analyses) |
| **Lim (2012)**^12^ | The diagnostic criteria for ISSNHL consisted of acute onset of hearing loss greater than 30 dB in 3 consecutive frequencies occurring within 3 days.  **Group 1**: Systemic steroid (oral Prednisolone)  **Group 2:** Intratympanic steroid (Dexamethasone)  **Group 3:**  Intratympanic steroid (Dexamethasone) +systemic steroid (oral prednisolone)  **Details of treatment protocol**: Group 1: Prednisolone (Solondo; Yuhan, Seoul, Korea) for 10 days on a schedule consisting of 60 mg/d for 5 days, 40 mg/d for 2 days, 20 mg/d for 2 days, and 10 mg/d for 1 day. Group 2: IT Dexamethasone (dexamethasone disodium phosphate, 5 mg/mL, 0.3-0.4 mL; Il Sung Pharm, Seoul,Korea) done twice weekly for 2 consecutive weeks.  Group 3: The protocol for Group 1 and 2 were administered simultaneously. | **Group 1:** 5.4 (3.1)  **Group 2:** 10.1 (8.1)  **Group 3**: 9.6 (7.5)  **QA:** High Risk of Bias  **Follow up time used in the analyses**: 21 days |
| **Dispenza (2011)**^14^ | SSHL of at least 30 dB across three contiguous frequencies over a period of 24 h.  **Group 1:** Intratympanic steroid (Dexamethasone)  **Group 2:** Oral steroid (Prednisolone)  **Details of treatment protocol**: The transtympanic injection of Dexamethasone 4 mg/ml was repeated weekly for a total of four injections. 60 mg of oral Prednisone tapered over 14 days. | **Group 1**: 9.4 (NR)  **Group 2**: 3.8 (NR)  **QA:** Unclear Risk of Bias  **Follow up time used in the analyses**: NA (it didn’t report the time point of interest) |
| **Park (2011)**^39^ | Unilateral sensorineural hearing loss with an average hearing loss of 30 dB in 3 consecutive frequencies within 3 days.  **Group 1:** Simultaneous IT-DEXA [Intratympanic dexamethasone plus systemic steroid (IV dexamethasone followed by oral prednisolone)]  **Group 2:** Subsequent IT-DEXA [systemic steroid (IV dexa followed by oral prednisolone) followed by Intratympanic dexamethasone]  **Details of treatment protocol**: All patients were hospitalized, and all were treated with intravenous dexamethasone for 7 days (10 mg/d for 5 days and 7.5 mg/d for 2 days) and then with oral steroids (prednisolone) for 3 days in tapered doses after the patients were discharged from the hospital. 0.3 to 0.4 ml of dexamethasone (5 mg/mL) was instilled intratympanically for total of 6 injections over 2 weeks. | **Group 1**: 3.52 (3.07)  **Group 2**: 3.00 (2.53)  **QA:** Unclear Risk of Bias  **Follow up time used in the analyses**: 90 days |
| **Filipo (2013)**^40^ | Patients diagnosed with ISSNHL* within 3 days from the onset, no previous therapy for ISSNHL, and age between 15 and 85 years.  *****ISSNHL was defined as,” a loss of at least 30 dB in three contiguous frequencies over a time course of 72 hours or less.” in introduction section of this study. The study included both children and adults.  **Group 1:** IT Prednisolone +/- oral prednisolone (oral prednisolone given if not recovered after 7 days of treatment)  **Group 2:** Placebo +/- oral prednisolone (oral prednisolone given if not recovered after 7 days of treatment)  **Details of treatment protocol**: Group 1: Intratympanic administration of 0.3 ml of prednisolone (Deltacortene Sol; Bruno Farmaceutici, Rome, Italy) at a dose of 62.5 mg/mL once a day for 3 consecutive days. Patients who did not show a complete recovery at T1 (7 days from the beginning of treatment) were treated with 8 days of oral prednisone at a tapering dose (62.5 mg per day for 4 days, followed by 37.5 mg for 2 days, and 25 mg for the last 2 days). Group 2: IT injection of 0.3 mL of a saline solution (0.9% sodium chloride) once a day for 3 consecutive days. The oral prednisone protocol was the same as for Group 1. | **Group 1:** 7 days for IT treatment; 17 days for oral treatment  **Group 2:** 7 days for IT treatment; 17 days for oral treatment  **QA:** Unclear Risk of Bias  **Follow up time used in the analyses**: 7 days |
| **Gordin (2002)**^42^ | ISHL (30 dB hearing loss in three frequencies) that had developed within 2 weeks of referral, with no evidence of known cause. (Not stated under the inclusion criteria: The treatment was started only in patients with normal renal function, as measured by normal values of urea and creatinine).  **Group 1:** Carbogen inhalation + intravenous MgSO4  **Group 2:** Carbogen inhalation  **Details of treatment protocol**: Group 1: intravenous therapy of 4 g of MgSO4 in 1000 ml of saline daily (14 drops/min) + carbogen (95% O2, 5% CO2) inhalation (for a half-hour every 2 hours). Group 2: carbogen (95% O2, 5% CO2) inhalation (for a half-hour every 2 hours). | **Group 1:** 4.4 (NR)  **Group 2**: 4.7 (NR)  **QA:** High Risk of Bias  **Follow up time used in the analyses**: NA (Both treatment arms, “MgSO4 + carbogen” and “carbogen”, were disconnected with the rest of network so we exclude this study) |
| **Yang (2010)**^43^ | A hearing loss of 30 dB or more over at least three contiguous frequencies within 72 hours. The cause of SSNHL in these patients was unknown.  **Group 1:** Zinc + [ IV Steroid (Dexamethasone)+ Radiopaque contrast (diatrizoate sodium) + Plasma expander (Dextran 40)]  **Group 2**: [ IV Steroid (Dexamethasone)+ Radiopaque contrast (diatrizoate sodium) + Plasma expander (Dextran 40)  **Details of treatment protocol**: Group 1: Oral zinc gluconate (Zinga 78 mg, 10 mg elemental zinc; Panion & BF Biotech, Tapei, Taiwan) 2 tablets twice a day (1 hour before breakfast and 1 hour before lunch) was given for 2 months. Intravenous corticosteroids (dexamethasone 5 mg/6 hours for 2 days, followed by 5 mg/8 hours for 2 days, and then 5 mg/12 hours for 1 day) and radiopaque contrast (diatrizoate sodium, Hypaque 76, 10 ml/day for 5 days)/plasma expander (low molecular weight dextran, Dextran 40, 500 ml/day for 5 days). Group 2: The same as for Group 1 except not receiving oral zinc. | **Group 1:** 4.8 (NR)  **Group 2:** 5.2 (NR)  **QA:** High Risk of Bias  **Follow up time used in the analyses**: 60 days |
| **Hong (2009)**^44^ | Patients with sensorineural hearing loss of 30 dB or more with over three contiguous audiometric frequencies that occurred in fewer than three days.  **Group 1:** Intratympanic steroid (dexamethasone)  **Group 2:** Oral steroid (prednisolone)+ other medications such as peripheral vasodilator, ginkgo biloba extract  **Details of treatment protocol**: Group 1: IT injections of 0.3 to 0.4 cc of dexamethasone (5 mg/mL) into the middle ear were performed (by anesthetizing the tympanic membrane with 10 percent Xylocaine) once a day over the course of eight days. Group 2: oral steroids for eight days in tapering doses (prednisolone 60 mg/d for 4 days, followed by 40 mg/d for 2 days, and 20 mg/d for 2 days) while also treating with other medications, such as a peripheral vasodilator and ginkgo biloba extract. | **Group 1:** 3.4 (NR)  **Group 2:** 3.9 (NR)  **QA:** Unclear Risk of Bias  **Follow up time used in the analyses**: 90 days |
| **Gundogan (2013)**^45^ | Patients with (1) unexplained sudden sensorineural hearing loss, which was defined as a sensorineural hearing loss of at least 30 dB at 3 contiguous frequencies over a period of 3 days; (2) time from the onset of hearing loss to the treatment of 14 days; (3) no initial treatment before; (4) no history of ear disease in the affected ear; (5) and unilateral sudden hearing loss.  **Group 1:** Combination therapy [intratympanic steroid (methylprednisolone) + oral steroid (methylprednisolone)  **Group 2:** Oral steroid (methylprednisolone)  **Details of treatment protocol**: IT methylprednisolone was performed 4 times for 2 consecutive weeks (once every 3 days). Patients were hospitalized for one week and received 14-day course of oral steroid (1 mg/kg of oral methylprednisolone and 10 mg taper every 3 days). | **Group 1:** 4.70 (4.00)  **Group 2:** 5.14 (3.52)  **QA:** High Risk of Bias  **Follow up time used in the analyses**: 28 days |
| **Bianchin (2010)**^46^ | The inclusion criterion was the presence of an acute, one sided SSHL that occurred not later than 20 days before the beginning of treatment, with a difference of at least 30 dB as compared to the unaffected ear in at least three connected frequencies. At diagnosis, the patients showed a value of LDL cholesterol >120 mg/dL and/or fibrinogen >320 mg/dL.  **Group 1:** HELP apheresis + standard treatment (glycerol infusion + intramuscular dexamethasone)  **Group 2:** Standard treatment (glycerol infusion + intramuscular dexamethasone)  **Details of treatment protocol**: Group 1: HELP apheresis plus standard treatment (HELP-ST) consisted of a single selective apheresis session (treated 3 L of plasma in 2 hours using a machine that monitors and controls fibrinogen/LDL apheresis on an outpatient basis.), followed by an infusion of 500 mL of glycerol, once a day for 10 days, and intramuscular administration of dexamethasone, 8 mg once a day for 10 days. Group 2: the same protocol as for Group 1 except not receiving HELP apheresis. | **Group 1**: 12 (NR)  **Group 2**: 13 (NR)  **QA:** Unclear Risk of Bias  **Follow up time used in the analyses**: NA (Insufficient data for PTA improvement; unclear definition for binary outcomes) |
| **Eftekharian (2015)**^47^ | Sensorineural hearing loss of 30 dB or more covering at least three contiguous audiometric frequencies, which occur within three days or fewer, no identifiable cause despite adequate investigation, normal or near normal hearing in the contralateral ear, age between 18–60 years, no more than 10 days from the onset of disease, no history of previous treatment, no contraindication for proposed therapy  **Group 1:** Intravenous steroid (methylprednisolone) + oral steroid (prednisolone)  **Group 2:** Oral steroid (prednisolone)  **Details of treatment protocol**: Group 1: 500-mg daily intravenous methylprednisolone for 3 consecutive days, followed by 1 mg/kg (maximum 60 mg) oral prednisolone for 11 days (total treatment: 14 days). Group 2: the same protocol as for Group 1 except not receiving IV steroid. | **Group 1:** 6.7 (2.2)  **Group 2**: 7.3 (2.3)  **QA:** Unclear Risk of Bias  **Follow up time used in the analyses**: 90 days |
| **Westerlaken (2003)**^13^, companion paper: Stokroos (1998)^5048^ | I) Sensorineural hearing loss of unknown cause; 2) hearing loss of at least 30 dB hearing level (HL) for 3 subsequent I-octave steps in frequency in the standard pure tone audiogram; 3) blank otologic history; and 4) hearing loss occurring within a period of 24 hours. The study included both children and adults.  **Group 1:** IV acyclovir + IV prednisolone  **Group 2:** Placebo + IV Prednisolone  **Details of treatment protocol**: Group 1: Intravenous prednisolone in a dose of 1 mg/kg body weight on day 1, to be diminished in equal steps to 0 mg over the course of 7 days. In addition, acyclovir 10 mg/kg body weight intravenously 3 times daily for 7 days. Group 2: the same protocol as for Group 1 except receiving placebo instead of acyclovir 10 mg/kg body weight intravenously 3 times daily for 7 days. | **Group 1**:4.4* (3.9)  **Group 2**: 4.2* (3.4)  *onset to study enrolment  **QA:** High Risk of Bias  **Follow up time used in the analyses**: NA (this study was excluded from analyses for failing to control for severity of initial hearing loss despite double blind randomization) |
| **Hunchaisri (2015)**^41^ | Patients with normal results of auditory brainstem response (ABR). Idiopathic sudden sensorineural hearing loss (ISSNHL) is defined as an abrupt onset of sensorineural hearing loss, within three days, and of at least 30 dB at three consecutive frequencies. The study included both children and adults.  **Group 1:** Chelated zinc + standard treatment (oral prednisolone + betahistine+ vitamin B1-6-12)  **Group 2:** Standard treatment (oral prednisolone + betahistine+ vitamin B1-6-12)  **Details of treatment protocol**: All patients received oral prednisolone, 60 mg/day in adults and 1 mg/kg/day in children, for seven days, combined with betahistine 12 mg and vitamin B1-6-12 three times a day for one month. In the zinc group, oral chelated zinc (75 mg, equivalent to 15 mg elemental zinc; Qualimed, Bangkok, Thailand) one tablet three times after meal was added for one month. | **Group 1:** 26.6 (37.8)  **Group 2:** 29.8 (40.9)  **QA:** Unclear Risk of Bias  **Follow up time used in the analyses**: NA (the study was not included in the network meta-analyses due to longer delay between onset and treatment. |
| **Wang (2013)**^48^ | Patients with a unilateral sensorineural hearing loss that developed at least 30 dB in three contiguous frequencies within 72 h, and with duration of onset 3 months or more without receiving prior treatment. the affected ear must have been at least 30 dB worse than that in the contralateral ear in at least 1 of the 4 PTA frequencies.  **Group 1:** Oral [Ginaton (extract of Ginkgo biloa leaves) + prednisone + mecobalamin]  **Group 2:** IV [Ginaton + IV Dexamethasone + IV mecobalamin]  **Details of treatment protocol:** The oral group took oral medication with Ginaton (extract of Ginkgo biloba leaves) tablets 80 mg/time and 3 times per day for 14 days, prednisone 1 mg/kg (60 mg/day maximum) daily for 5 days followed by a 5-day taper (50, 40, 30, 20, and to 10 mg) for a total of 10 days of treatment, and mecobalamin tablets 500μg/time and 3 times/day for 14 days. The intravenous group administered by intravenous injection received Ginaton injection with a dose of 105 mg every day by intravenous injection for 14 days, dexamethasone 10 mg/day for 5 days followed by a dose of 5 mg/day for 5 days, and mecobalamin injection 500μg/ day by intravenous injection for 14 days. | **Group 1:** Mean years from onset to study enrolment: 8.2 (range 0.5-19)  **Group 2:** Mean years from onset to study enrolment: 7.6 (range 0.25-17)  **QA:** Unclear Risk of Bias  **Follow up time used in the analyses**: NA (the study was not included in the network meta-analyses due to longer delay between onset and treatment. |
| **Kosyakov (2012)**^52^ | Patients with SSNHL with hearing loss in 3 contiguous frequencies of at least 30 dB, who had not  previously been treated and were at least 18 years old.  **Group 1**: IT steroid (Dexamethasone)  **Group 2**: IV steroid (Dexamethasone)+IV (Pentoxifylline+Cocarboxylase+Potassium and magnesium aspartate)+IM vitamin B-complex  **Group 3**: IV steroid (Dexamethasone)  **Details of treatment protocol**: Patients in group 1 received Dexamethasone (Dex) through a tympanostomy tube fixed in the posteroinferior quadrant of the tympanic membrane 4mg/cc every day for 10 days, 4 mg every other day over 20 days and then 4 mg 2 times a week over 5 months by injecting the drug in the tympanic cavity through the tympanostomy tube. Patients in group 2 received Dexamethasone (0.1 mg/kg) in 200 ml of isotonic solution intravenously; Pentoxifylline, Cocarboxylase, Potassium and magnesium aspartate intravenously and vitamin B-complexe intramuscularly were administered daily for 10 days. Patients in group 3 received intravenous administration of Dexamethasone (0.1 mg/kg) daily over 10 days. | **Groups 1-3:** < 1 month between the onset and the beginning of the therapy  **QA:** Unclear Risk of Bias  **Follow up time used in the analyses**: 30 days |
| **Swachia (2016)**^54^ | Patients aged between 18 and 65 years reporting SSNHL who met NIDCD criteria† were included.  **Group 1**: Oral steroid (prednisone)  **Group 2**: IT steroid (methylprednisolone)  **Details of treatment protocol**: Patients in group I were treated with oral prednisone. The dosage was 1 mg/kg body weight for the first 10 days. The drug was tapered to 0.5 mg/kg body weight for the next 2 days and then 0.25 mg/kg body weight for 2 days. Patients in group 2 received intratympanic methylprednisolone. One milliliter of the drug solution containing 40 mg of the drug (40 mg/mL) was injected into the middle ear cavity through transtympanic route. The drug was injected twice a week for 2 weeks in a row.  † The National Institute on Deafness and Other Communication Disorders (NIDCD) has defined SSNHL as a subjective sensation of hearing impairment in one or both ears developing within 72 h with a decrease in hearing of more than or equal to 30 decibels (dB) on three consecutive frequencies in comparison to a normal ear on audiometry. | **Groups 1-2:** < 14 days between the onset and inclusion  **QA:** Unclear Risk of Bias  **Follow up time used in the analyses**: 60 days |
| **Tsounis (2017)**^53^ | Eligible subjects were adults aged 18 years or older both male and female with minimum 30 dB HL hearing loss in three consecutive octaves that had occurred within a course of 3 days.  **Group 1**: IV steroid (prednisolone) + PO steroid (methylprednisolone)  **Group 2**: IT steroid (IT methylprednisolone)  **Group 3**: Combination steroids (IV+IT+ Oral steroids: prednisolone+ methylprednisolone)  **Details of treatment protocol**: Patients in group 1 received 1 mg/kg of body weight prednisolone per day for 7 days followed by 0.5 mg/kg of body weight prednisolone per day for another 3 days. After completing this course, patients were discharged and continued their treatment with oral methylprednisolone 32 mg/ day for 4 days followed by oral methylprednisolone 16 mg/ day for another 3 days. Patients in group 2 received analgesia and while lying in the supine position with the head tilted 45° to the healthy side a 25-gauge spinal needle was introduced into the posterior–inferior quadrant of the tympanic membrane, and 0.4–0.6 ml of 62.5 mg/ml methylprednisolone were slowly instilled intratympanically into the middle ear cleft. Patients were then instructed to avoid moving or swallowing for 20 min to create the optimal conditions for the solution to fill the round window niche. Intratympanic methylprednisolone injections were performed on the day of presentation, 3, 5 and 10 days after presentation (total of 4 times). In case complete recovery was confirmed by a pure tone audiogram the treatment was interrupted. Patients in group 3 received combination of both drugs in group 1 and 2. | **Group 1:** 3.1±3.0  **Group 2:** 4.6±3.0  **Group 3:** 4.0±3.9  **QA:** Unclear Risk of Bias  **Follow up time used in the analyses**: 90 days |
| **Ermutlu (2017)**^33^ | All patients in the study population presented with unilateral SSHL of at least 30 dB including at least three frequencies and occurring within 72 h. Additional inclusion criteria were as follows: (1) age between 18 and 80 years, (2) time prior to treatment not exceeding 7 days, and (3) no history of previous treatment.  **Group 1**: Oral steroid (prednisolone)  **Group 2**: IT steroid (dexamethasone)  **Details of treatment protocol**: Patients in group 1 received Prednisolone was administered for the OS group starting with a daily divided dose of 1 mg/kg (maximum 80 mg) and tapering 10 mg every 3 days. In group 2 patients ITS administration was performed three times every other day using a surgical microscope. Patient was placed with the head tilted 45° to the opposite side. Local anesthesia was administered by applying a 10% lidocaine-soaked cotton ball onto the tympanic membrane for approximately 10 min. Following the creation of an anterosuperior puncture with a 22-gauge needle for ventilation, a 27-gauge needle was introduced in the posteroinferior quadrant of the tympanic membrane to deliver 0.5–0.7 cc dexamethasone (DXM) (8 mg/2 ml) through the tympanic membrane. The patient was instructed to remain in the otologic position and to avoid swallowing or talking for 30 min. The tympanic membrane was regularly checked until the perforation was healed | **Group 1:** 2.69 (NR)  **Group 2:** 3.74 (NR)  **QA:** High Risk of Bias  **Follow up time used in the analyses**: 90 days |

*Time of outcome assessment used in the analysis, and in the time-adjusted analyses.

†In the version updated in March 2017, NIDCD definition of SSHL had removed “within 72 h” https://www.nidcd.nih.gov/health/sudden-deafness

Abbreviations: QA: quality assessment; NA= not applicable; NR= not reported

**Table D**: Risk of bias assessment for the included studies

| **Domain** | **Support for judgment** | | **Review authors’ judgment** | | **Overall Study Risk** | |
| --- | --- | --- | --- | --- | --- | --- |
| **Hultcrantz et al. (2014)**^4,49,51^ | | | | | Unclear | |
| **Selection bias** |  | |  | |  | |
| Random sequence generation | Randomization was performed by Apoteket Production & Laboratories (APL Stockholm) independent of the participating researchers in permuted blocks of 10 and allocation ratio of 1:1. | | Low | |  |  |
| Allocation concealment | Central; The randomization code was double-blinded and kept by APL. | | Low | |  |  |
| **Performance bias** |  | |  | |  |  |
| Blinding of participants and personnel | The study stated that it was triple blinded, so perhaps the participants and personal; Table 1 show no significant different in baseline characteristics between the arms of RCT. In the thesis paper, they mention that outcome assessors were also blinded; also in 229 they mention blinding of outcome assessors | | Low | |  |  |
| **Detection bias** |  | |  | |  |  |
| Blinding of outcome assessment | Unclear if outcome assessment was blinded or not, although as no details are provided, it seems unlikely. However, outcomes of interest reported in this paper are not deemed likely to be affected by lack of blinding. | | Low | |  |  |
| **Attrition bias** |  | |  | |  |  |
| Incomplete outcome data | only 2 patients (4.4%) in the treatment arm were having missing data and were excluded from analyses. | | Low | |  |  |
| **Reporting bias** |  | |  | |  |  |
| Selective reporting | no protocol and registry is reported; there are companion papers but none refer to protocol/registry; had two sets of primary and secondary outcome, so unclear if selective outcome reporting. | | Unclear | |  |  |
| **Other bias** |  | |  | |  |  |
| Other sources of bias | Non-industry funding ( This trial was supported by grants from the Medical Research Council of Southeast Sweden (FORSS)) and no conflict of interest, no other notable issue | | Low | |  |  |
| **Outcomes** | PTA improvement, hearing recovery | |  | |  | |
| **Koo et al. (2015)**^38^ | | | | | High | |
| **Selection bias** |  | |  | |  | |
| Random sequence generation | Random allocation sequence was generated by a statistician of Yuyu Pharma through the website. Randomized block design (block size 6) was used. | | Low | |  |  |
| Allocation concealment | Yuyu Pharma kept the investigators blinded. Principal and sub-investigators of each hospital enrolled participants and assigned participants to interventions according to randomization numbers. | | Low | |  |  |
| **Performance bias** |  | |  | |  |  |
| Blinding of participants and personnel | Yuyu Pharma supplied the active product and the placebo product of the same shape to the three institutions and maintained blinding of researchers. Principal and sub-investigators of each hospital enrolled participants and assigned participants to interventions according to randomization numbers | | Low | |  |  |
| **Detection bias** |  | |  | |  |  |
| Blinding of outcome assessment | "After completion of the clinical trial, the results were revealed and analyzed by outcome evaluators". | | High | |  |  |
| **Attrition bias** |  | |  | |  |  |
| Incomplete outcome data | 20% attrition in the control, and 4-17% in the treatment arm; reasons for 3 pts in treatment and 4 pts in control is not known, the study didn't do anything e.g. imputation | | High | |  |  |
| **Reporting bias** |  | |  | |  |  |
| Selective reporting | The protocol mentions Speech detection threshold (SDT) as one of the outcomes but it is not reported in the article. Instead Speech discrimination score (SDS ) is reported which is not mentioned in the protocol. No explanation is given for this discrepancy. | | High | |  |  |
| **Other bias** |  | |  | |  |  |
| Other sources of bias | The study was funded by YuYu Pharma. No further explanation is provided in how Pharma was involved in the results and their interpretation. | | Unclear | |  |  |
| **Outcomes** | PTA improvement, Hearing recovery, ACT, BCT, SDS, THI, SRT, Harm | |  | |  | |
| **Lim et al. (2012)**^12^ | | | | | High | |
| **Selection bias** |  | |  | |  | |
| Random sequence generation | The method of randomization is a consecutive allocation by visit sequence (not sure what exactly visit sequence mean). The 60 patients were randomly, prospectively, and equally (n = 20 per group) assigned to 3 groups based on the method of steroid administration: oral route (group I), intratympanic dexamethasone injection (ITDI; group II), and oral 1 ITDI (group III). | | High | |  |  |
| Allocation concealment | The method of randomization is a consecutive allocation by visit sequence. No further info if it was concealed. | | High | |  |  |
| **Performance bias** |  | |  | |  |  |
| Blinding of participants and personnel | Given that the study interventions had different routes of administration blinding would not be feasible based on the expert opinion. | | Low | |  |  |
| **Detection bias** |  | |  | |  |  |
| Blinding of outcome assessment | Outcome assessors were blinded to pts allocation. | | Low | |  |  |
| **Attrition bias** |  | |  | |  |  |
| Incomplete outcome data | No attrition. | | Low | |  |  |
| **Reporting bias** |  | |  | |  |  |
| Selective reporting | no protocol or registry reported. | | Unclear | |  |  |
| **Other bias** |  | |  | |  |  |
| Other sources of bias | no funding, no conflict of interest, no other noted issue | | Low | |  |  |
| **Outcomes** | PTA improvement, Extend recovery | |  | |  | |
| **Dispenza et al. (2011)**^14^ | | | | | Unclear | |
| **Selection bias** |  | |  | |  | |
| Random sequence generation | "The patients were randomly divided in two groups according to treatment" no more information is provided | | Unclear | |  |  |
| Allocation concealment | insufficient information | | Unclear | |  |  |
| **Performance bias** |  | |  | |  |  |
| Blinding of participants and personnel | It was not feasible due to different route of administration. | | Low | |  |  |
| **Detection bias** |  | |  | |  |  |
| Blinding of outcome assessment | It's unclear whether audiologists/ outcome assessors were blinded to allocation or not. | | unclear | |  |  |
| **Attrition bias** |  | |  | |  |  |
| Incomplete outcome data | Total loss is around 9%; however, the one with unknown reason is 5.8% in total. It is also not known that the loss belongs to which arm. | | Unclear | |  |  |
| **Reporting bias** |  | |  | |  |  |
| Selective reporting | No protocol or registry reported | | Unclear | |  |  |
| **Other bias** |  | |  | |  |  |
| Other sources of bias | No conflict of interest but unclear funding, no other issue observed. | | Unclear | |  |  |
| **Outcomes** | Clinical recovery time, Hearing recovery, PTA improvement | |  | |  | |
| **Park et al. (2011)**^39^ | | | | | Unclear | |
| **Selection bias** |  | |  | |  | |
| Random sequence generation | Randomization was performed using a table of random numbers and SPSS software (version 11.0 for Windows; SPSS, Inc, an IBM Company, Chicago, Illinois). | | Low | |  |  |
| Allocation concealment | Insufficient info | | Unclear | |  |  |
| **Performance bias** |  | |  | |  |  |
| Blinding of participants and personnel | Both arms received the same exact treatment but different timing (simultaneously or subsequently to systemic steroid), so less likely to create problem if the participants and physicians were unblinded. | | Unclear | |  |  |
| **Detection bias** |  | |  | |  |  |
| Blinding of outcome assessment | The outcome assessors (audiologists) were blinded | | Low | |  |  |
| **Attrition bias** |  | |  | |  |  |
| Incomplete outcome data | Only 4% were lost to follow up with reasonable reasons and balanced across the arms | | Low | |  |  |
| **Reporting bias** |  | |  | |  |  |
| Selective reporting | no registry or protocol reported | | Unclear | |  |  |
| **Other bias** |  | |  | |  |  |
| Other sources of bias | no funding, no conflict of interest, no other noted issue | | Low | |  |  |
| **Outcomes** | Clinical recovery time, Hearing recovery, PTA improvement | |  | |  | |
| **Filipo et al. (2013)**^40^ | | | | | Unclear | |
| **Selection bias** |  | |  | |  | |
| Random sequence generation | "The 50 patients were randomized into two groups" no more information is provided. | | Unclear | |  |  |
| Allocation concealment | No information | | Unclear | |  |  |
| **Performance bias** |  | |  | |  |  |
| Blinding of participants and personnel | Triple blinded | | Low | |  |  |
| **Detection bias** |  | |  | |  |  |
| Blinding of outcome assessment | It says triple blinded, but we are not sure if outcome assessors were actually blinded. | | Unclear | |  |  |
| **Attrition bias** |  | |  | |  |  |
| Incomplete outcome data | No attrition | | Low | |  |  |
| **Reporting bias** |  | |  | |  |  |
| Selective reporting | no dropouts | | Low | |  |  |
| **Other bias** |  | |  | |  |  |
| Other sources of bias | no protocol or registry reported | | Unclear | |  |  |
| **Outcomes** | Harm, Hearing recovery, PTA improvement | |  | |  | |
|  |  | |  | |  | |
| **Gordin et al. (2002)**^42^ | | | | | Unclear | |
| **Selection bias** |  | |  | |  | |
| Random sequence generation | No info | | Unclear | |  |  |
| Allocation concealment | No info | | Unclear | |  |  |
| **Performance bias** |  | |  | |  |  |
| Blinding of participants and personnel | No info | | unclear | |  |  |
| **Detection bias** |  | |  | |  |  |
| Blinding of outcome assessment | No info | | unclear | |  |  |
| **Attrition bias** |  | |  | |  |  |
| Incomplete outcome data | Fifty-nine patients in the carbogen group had inadequate follow-up information and were excluded from the study, leaving a final group of 60 patients. Fifty-six patients were lost to follow-up in the Mg2+ group, leaving 73 patients. Additionally, 20 and 10 were excluded due to other reasons (10 due to side effect and 20 due to contamination). No additional information regarding the characteristics of lost to follow up pts. | | High | |  |  |
| **Reporting bias** |  | |  | |  |  |
| Selective reporting | only improvement was reported | | Unclear | |  |  |
| **Other bias** |  | |  | |  |  |
| Other sources of bias | no info about funding and conflict of interest, no other noted issue | | Unclear | |  |  |
| **Outcomes** | Hearing recovery (improvement) | |  | |  | |
|  |  | |  | |  | |
| **Yang et al. (2010)**^43^ | | | | | High | |
| **Selection bias** |  | |  | |  | |
| Random sequence generation | insufficient info | | Unclear | |  |  |
| Allocation concealment | no info | | Unclear | |  |  |
| **Performance bias** |  | |  | |  |  |
| Blinding of participants and personnel | no blinding of patients but it could have been done because both were oral; the study recommends double blinding in the future studies | | High | |  |  |
| **Detection bias** |  | |  | |  |  |
| Blinding of outcome assessment | Outcome assessors were blinded to the patients’ allocation during the audiometry test | | Low | |  |  |
| **Attrition bias** |  | |  | |  |  |
| Incomplete outcome data | no dropout | | Low | |  |  |
| **Reporting bias** |  | |  | |  |  |
| Selective reporting | no protocol or registry reported | | Unclear | |  |  |
| **Other bias** |  | |  | |  |  |
| Other sources of bias | no info about funding and conflict of interest | | Unclear | |  |  |
| **Outcomes** | PTA improvement, Hearing recovery | |  | |  | |
| **Hong et al. (2009)**^44^ | | | | | Unclear | |
| **Selection bias** |  | |  | |  | |
| Random sequence generation | Insufficient information about the sequence generation process | | Unclear | |  |  |
| Allocation concealment | Insufficient information | | Unclear | |  |  |
| **Performance bias** |  | |  | |  |  |
| Blinding of participants and personnel | unblinded but it was oral vs intratympanic, so it will not be penalized based on expert opinion | | Low | |  |  |
| **Detection bias** |  | |  | |  |  |
| Blinding of outcome assessment | Outcome assessors were blinded. | | Low | |  |  |
| **Attrition bias** |  | |  | |  |  |
| Incomplete outcome data | >10% losses in each group with no known reason for 10% of them | | Unclear | |  |  |
| **Reporting bias** |  | |  | |  |  |
| Selective reporting | no protocol or registry reported | | Unclear | |  |  |
| **Other bias** |  | |  | |  |  |
| Other sources of bias | no conflict of interest, funding seems non-industry (BioGreen21 Program, Rural Development Administration, Republic of Korea), no other noted issue | | Low | |  |  |
| **Outcomes** | PTA improvement, Hearing recovery | |  | |  | |
| **Gundogan et al. (2013)**^45^ | | | | | High | |
| **Selection bias** |  | |  | |  | |
| Random sequence generation | The blocked randomization was used in this study. | | Low | |  |  |
| Allocation concealment | insufficient information | | Unclear | |  |  |
| **Performance bias** |  | |  | |  |  |
| Blinding of participants and personnel | Unblinded but blinding not feasible because different rout of administration (combination vs oral). | | Low | |  |  |
| **Detection bias** |  | |  | |  |  |
| Blinding of outcome assessment | Outcome assessors were unblinded. | | High | |  |  |
| **Attrition bias** |  | |  | |  |  |
| Incomplete outcome data | 7.5% of participants were excluded from analyze because they didn't make it to the 1 month visit (no reason is given) in both groups | | Unclear | |  |  |
| **Reporting bias** |  | |  | |  |  |
| Selective reporting | no protocol or registry reported | | Unclear | |  |  |
| **Other bias** |  | |  | |  |  |
| Other sources of bias | no conflict of interest and no funding; no other noted issue | | Low | |  |  |
| **Outcomes** | Harm, SDS, Hearing recovery, PTA improvement | |  | |  | |
| **Hunchaisri et al. (2005)**^41^ | | | | | High | |
| **Selection bias** |  | |  | |  | |
| Random sequence generation | Insufficient info | | Unclear | |  |  |
| Allocation concealment | Insufficient info | | Unclear | |  |  |
| **Performance bias** |  | |  | |  |  |
| Blinding of participants and personnel | The study doesn't mention anything about blinding. We assume it was unblinded trial. Both arms received standard treatment via same rout of administration (oral) and could have been blinded to their allocations. This unblinding could have influenced the participants to seek zinc outside the study. | | Unclear | |  |  |
| **Detection bias** |  | |  | |  |  |
| Blinding of outcome assessment | The study doesn't mention anything about blinding. We assume it was unblinded trial. Both arms received standard treatment via same rout of administration (oral) and could have been blinded to their allocations. This unblinding could have influenced the participants to seek zinc outside the study. | | Unclear | |  |  |
| **Attrition bias** |  | |  | |  |  |
| Incomplete outcome data | Of total 40 subjects, 10 were excluded. Of ten patients excluded, one had acoustic neuroma, one had diabetes, three had hyperlipidemia, three had drug adverse effect (nausea, vomiting, and vertigo), and two were lost to follow-up. Of these, 5 have fair reasons for exclusion but it is unclear that the other five (3 due to adverse effect and 2 lost to follow up) belong to which arm, if they were balanced across groups, how their characteristics differed than those remained in the study, and if that would affect the results (12.5%). | | Unclear | |  |  |
| **Reporting bias** |  | |  | |  |  |
| Selective reporting | no protocol or registry is reported | | Unclear | |  |  |
| **Other bias** |  | |  | |  |  |
| Other sources of bias | Funding is not reported. No other issue noted. | | Unclear | |  |  |
| **Outcomes** | Harm, SDS, hearing recovery, PTA improvement | |  | |  | |
| **Wang et al. (2013)**^48^ | | | | | Unclear | |
| **Selection bias** |  | |  | |  | |
| Random sequence generation | The randomization codes were computer generated using SAS software (SAS Institute Inc, Cary, North Carolina). | | Low | |  |  |
| Allocation concealment | Insufficient info | | Unclear | |  |  |
| **Performance bias** |  | |  | |  |  |
| Blinding of participants and personnel | Unblinded but different routes of administration, so blinding not feasible/Audiologists were blinded to treatment. | | Low | |  |  |
| **Detection bias** |  | |  | |  |  |
| Blinding of outcome assessment | Unblinded but different routes of administration, so blinding not feasible/Audiologists were blinded to treatment. | | Low | |  |  |
| **Attrition bias** |  | |  | |  |  |
| Incomplete outcome data | No lost to follow up | | Low | |  |  |
| **Reporting bias** |  | |  | |  |  |
| Selective reporting | no protocol or registry is reported | | Unclear | |  |  |
| **Other bias** |  | |  |  | | |
| Other sources of bias | Non-industry funding but no declaration of conflict of interest; no other noted issue | | Unclear |  |  |  |
| **Outcomes** | PTA improvement, hearing recovery | |  |  | | |
| **Kosyakov et al. (2017)**^52^ | | | | | Unclear | |
| **Selection bias** |  | |  | |  | |
| Random sequence generation | The division into groups was based on mechanical randomization. | | Unclear | |  |  |
| Allocation concealment | No information | | Unclear | |  |  |
| **Performance bias** |  | |  | |  |  |
| Blinding of participants and personnel | No information | | Unclear | |  |  |
| **Detection bias** |  | |  | |  |  |
| Blinding of outcome assessment | No information | | Unclear | |  |  |
| **Attrition bias** |  | |  | |  |  |
| Incomplete outcome data | 9 in one arm and 12 in other were withdrawn due to loss of sleep; one developed otitis media and was excluded but not sure in which arm. There are losses of 12-16% and unbalanced | | Unclear | |  |  |
| **Reporting bias** |  | |  | |  |  |
| Selective reporting | No protocol, no registry | | Unclear | |  |  |
| **Other bias** |  | |  | |  |  |
| Other sources of bias | No information about funding and conflict of interest. | | Unclear | |  |  |
| **Outcomes** | PTA improvement, hearing recovery | |  | |  | |
| **Ermutlu et al. (2017)**^33^ | | | | | High | |
| **Selection bias** |  | |  | |  | |
| Random sequence generation | insufficient information | | Unclear | |  |  |
| Allocation concealment | insufficient information | | Unclear | |  |  |
| **Performance bias** |  | |  | |  |  |
| Blinding of participants and personnel | Unblinded but different routes of administration, so blinding not feasible | | Low | |  |  |
| **Detection bias** |  | |  | |  |  |
| Blinding of outcome assessment | insufficient information | | Unclear | |  |  |
| **Attrition bias** |  | |  | |  |  |
| Incomplete outcome data | Losses were 17% and unbalanced (more in oral arm) | | High | |  |  |
| **Reporting bias** |  | |  | |  |  |
| Selective reporting | No protocol or registry; the trial does not report PTA improvement (continuous outcome) which is reported by almost all (15) studies on this topic; however, it reports other outcomes (not of interest to us) and we gave it the benefit of doubt that it may not have enough space to report PTA continuous data. | | Unclear | |  |  |
| **Other bias** |  | |  |  | | |
| Other sources of bias | No information about funding; authors declare no conflict of interest | | Unclear |  |  |  |
| **Outcomes** | Hearing recovery (binary outcome) | |  |  | | |
| **Swachia et al. (2016)**^54^ | | | | | | Unclear |
| **Selection bias** | |  | |  | |  |
| Random sequence generation | | Insufficient info | | Unclear | |  |
| Allocation concealment | | Insufficient info | | Unclear | |  |
| **Performance bias** | |  | |  | |  |
| Blinding of participants and personnel | | Unblinded but different routes of administration, so blinding not feasible/Audiologists were blinded to treatment. | | Low | |  |
| **Detection bias** | |  | |  | |  |
| Blinding of outcome assessment | | Insufficient info | | Unclear | |  |
| **Attrition bias** | |  | |  | |  |
| Incomplete outcome data | | Insufficient info | | Unclear | |  |
| **Reporting bias** | |  | |  | |  |
| Selective reporting | | no protocol or registry is reported, Insufficient info | | Unclear | |  |
| **Other bias** | |  | |  | |  |
| Other sources of bias | | No conflict of interest and funding declared. | | Low | |  |
| **Outcomes** | | PTA improvement, hearing recovery (categorical) | |  | |  |

| **Tsounis et al. (2017)**^53^ | | | Unclear |
| --- | --- | --- | --- |
| **Selection bias** |  |  |  |
| Random sequence generation | Randomization was accomplished by generating sequential random numbers (sequential randomization) using a computer-based software. | Low |  |
| Allocation concealment | The random numbers were placed in closed envelopes and were given sequentially to every patient that was recruited. | Low |  |
| **Performance bias** |  |  |  |
| Blinding of participants and personnel | Not feasible due to different medication administration route. "Treating physicians and patients were aware of the allocated" | Low |  |
| **Detection bias** |  |  |  |
| Blinding of outcome assessment | The physicians that performed the audiologic assessment and data analysis were kept blinded to the allocation. | Low |  |
| **Attrition bias** |  |  |  |
| Incomplete outcome data | Losses was 22% and almost balanced across groups | Unclear |  |
| **Reporting bias** |  |  |  |
| Selective reporting | Study protocol is not accessible; however, they report both PTA (continuous), and binary outcome (extent of recovery) | Unclear |  |
| **Other bias** |  |  |  |
| Other sources of bias | Funding is not reported, conflict of interest was declared as none. | Unclear |  |
| **Outcomes** | PTA improvement, hearing recovery, adverse events |  |  |

**Further details on hearing measures assessed via SRT, BCT and ACT that were reported by single studies:**

SRT percentage: SRT, the minimum hearing level for speech at which an individual can just detect the presence of speech material 50% of the time, was improved (subjects were determined to be ‘improved’ if the threshold was reduced by more than 10 dB), in 18 of 24 patients (75%) that received IV EGb761 (Ginkgo bilbo extract) + methylprednisolone + Oral EGb761 but it remained unchanged or aggravated (subjects were determined to be aggravated if the threshold was increased by more than 10 dB and ‘unchanged’ if the difference was less than 10 dB.) in 6 patients on day 28±2 post treatment^38^. Similarly, SRT percentage was improved in 17/24 patients (68%) that received placebo+ methylprednisolone+ Oral EGb761 but remained unchanged or aggravated in 7/24 (29.16%) on day 28±2 post- treatment^38^. The post-treatment SRT percentage did not differ between the two arms on day 28 [Mean (SD) was 32.04(31.91) in IV EGb761 group compared to 39.04(30.23) in placebo arm; p=0.232]^38^.

BCT: BCT was improved (subjects were determined to be ‘improved’ if the threshold was reduced by more than 10 dB) in 19 of 24 patients (79.16%) that received IV EGb761 (Ginkgo bilbo extract) + methylprednisolone + Oral EGb761, but it remained unchanged or aggravated(subjects were determined to be aggravated if the threshold was increased by more than 10 dB and ‘unchanged’ if the difference was less than 10 dB.) in 5 (20.83%) patients on day 28^th^ post-treatment^38^. Similarly, BCT was improved in 15/24 patients (62.5%) that received placebo+ methylprednisolone+ Oral EGb761 but remained unchanged or aggravated in 9/24 (37.5%) on day 28^th^ post-treatment^38^. The post-treatment BCT did not demonstrate a statistically significant difference between the two arms on day 28 [Mean (SD) was 22.51(23.77) in IV EGb761 group compared to 30.64(23.13) in placebo arm, p=0.082]^38^.

ACT: ACT was improved (subjects were determined to be ‘improved’ if the threshold was reduced by more than 10 dB) in 21 of 24 patients (87.5%) that received IV EGb761 (Ginkgo bilbo extract) + methylprednisolone + Oral EGb761, but it remained unchanged or aggravated(subjects were determined to be aggravated if the threshold was increased by more than 10 dB and ‘unchanged’ if the difference was less than 10 dB) in 3 (12.5%) patients on day 28^th^ post-treatment^38^. Similarly, ACT was improved in 18/24 patients (75%) that received placebo+ methylprednisolone+ Oral EGb761 but remained unchanged or aggravated in 6/24 (25%) on day 28^th^ post- treatment^38^. The post-treatment ACT did not demonstrate a statistically significant difference between the two arms on day 28 [Mean (SD) was 23.84(25.42) in IV EGb761 group compared to 34.63(28.90) in placebo arm, p=0.082]^38^.
