# Supplementary material for: A systematic review and network meta-analysis of existing pharmacologic therapies in patients with idiopathic sudden sensorineural hearing loss
Source: PLoS One. 2019 Sep 9;14(9):e0221713. doi: 10.1371/journal.pone.0221713 (PMC6733451; doi:10.1371/journal.pone.0221713)
Supplement: S3 Text — (DOCX) [file pone.0221713.s003.docx]

# S3 Text: Additional Information Regarding Network Meta-Analyses

For studies which reported PTA at baseline and at final follow-up with corresponding standard deviations (but without average changes and corresponding standard deviations per group), we calculated the mean changes from baseline and imputed the standard errors of the mean changes as

$$\delta_{ij}=\bar{x}_{ij}-\bar{y}_{ij}$$

$${SE}^{2}\left( \delta_{ij} \right)={{SD}^{2}\left( x_{ij} \right)}/{N_{ij}}+{{SD}^{2}\left( y_{ij} \right)}/{N_{ij}}-{2 \rho SD\left( x_{ij} \right) SD\left( y_{ij} \right)}/{N_{ij}}$$

where $SD\left( x_{ij} \right)$ and $SD\left( y_{ij} \right)$ denote the standard deviation of the PTA $x_{ij}$ at baseline and $y_{ij}$ at follow-up, respectively, and $N_{ij}$ is the sample size of the $j^{\mathrm{th}}$ arm in the $i^{\mathrm{th}}$ study. The correlation coefficient between outcomes at baseline and at follow-up were assigned a Uniform prior distribution, $\rho\sim\mathrm{Uniform}(0.5, 1)$, since the correlation ranged 0.53-0.92 calculated based on the SDs of hearing gain, initial and final PTA per arm reported from three studies^12,43,54^.

**Table A: Summary of Model Fit Details for Network Meta-Analyses**

| **Model** | **No. of Studies / Arms** | **Total Residual Deviance** | **Deviance Information Criteria** | **SD (95% CrI)** |
| --- | --- | --- | --- | --- |
| **PTA Improvement** | | | | |
| FE Consistency | 9 / 21 | 32.78 | 130.0 | NA |
| RE Consistency | 9 / 21 | 21.59 | 122.9 | 7.02 (1.57, 15.33) |
| RE Unrelated Means | 9 / 21 | 21.47 | 123.5 | 6.69 (0.84, 16.77) |
| **Responders’ Recovery** | | | | |
| FE Consistency | 10 / 23 | 33.40 | 125.7 | NA |
| RE Consistency | 10 / 23 | 24.03 | 121.9 | 0.96 (0.17, 2.13) |
| RE Unrelated Means | 10 / 23 | 22.31 | 119.7 | 0.61 (0.03, 1.92) |
| **Total Recovery** | | | | |
| FE Consistency | 9 / 21 | 28.57 | 115.9 | NA |
| RE Consistency | 9 / 21 | 21.17 | 113.1 | 0.83 (0.11, 1.94) |
| RE Unrelated Means | 9 / 21 | 19.51 | 111.2 | 0.53 (0.02, 1.92) |

**Table B: The number of studies and patients related to each treatment node in the network diagram without complementary medicine interventions (Figure 2 left panel).**

| **Nodes** | **Numbers of arms and patients** | | |
| --- | --- | --- | --- |
|  | **PTA improvement** | **Total recovery** | **Responders’ recovery** |
| **Placebo** | **2 arms from 2 studies, 71 patients:** Placebo (2) | **2 arms from 2 studies, 67 patients:** Placebo (2) | **2 arms from 2 studies, 67 patients:** Placebo (2) |
| **PO steroid** | **6 arms from 6 studies, 177 patients:**  PO prednisolone (4)  PO methylprednisolone (1)  PO prednisone (1) | **6 arms from 6 studies, 168 patients:**  PO prednisolone (5)  PO methylprednisolone (1) | **7 arms from 7 studies, 189 patients:**  PO prednisolone (5)  PO methylprednisolone (1) PO prednisone (1) |
| **IV steroid** | **2 arms from 1 study, 50 patients:** IT dexamethasone (2) | **2 arms from 1 study, 50 patients:** IT dexamethasone (2) | **2 arms from 1 study, 50 patients:** IT dexamethasone (2) |
| **IV + PO steroid** | **2 arms from 2 studies, 64 patients:**  IV methylpred + PO prednisolone (1)  IV prednisolone + PO methylpred (1) | **2 arms from 2 studies, 64 patients:**  IV methylpred + PO prednisolone (1)  IV prednisolone + PO methylpred (1) | **2 arms from 2 studies, 64 patients:**  IV methylpred + PO prednisolone (1)  IV prednisolone + PO methylpred (1) |
| **IT steroid** | **6 arms from 6 studies, 149 patients:** IT dexamethasone (3)  IT methylprednisolone (2)  IT prednisolone (1) | **6 arms from 6 studies, 143 patients:** IT dexamethasone (3)  IT methylprednisolone (2)  IT prednisolone (1) | **7 arms from 7 studies, 168 patients:** IT dexamethasone (4)  IT methylprednisolone (2)  IT prednisolone (1) |
| **IT + systemic steroid** | **3 arms from 3 studies, 90 patients:**  IT dexamethasone + PO prednisolone (1)  IT + PO/taper methylprednisolone (1)  IT methylprednisolone + IV prednisolone + PO methylprednisolone (1) | **3 arms from 3 studies, 90 patients:**  IT dexamethasone + PO prednisolone (1)  IT + PO/taper methylprednisolone (1)  IT methylprednisolone + IV prednisolone + PO methylprednisolone (1) | **3 arms from 3 studies, 90 patients:**  IT dexamethasone + PO prednisolone (1)  IT + PO/taper methylprednisolone (1)  IT methylprednisolone + IV prednisolone + PO methylprednisolone (1) |

**Figures A-D are from the PTA improvement analyses**

**Figure A

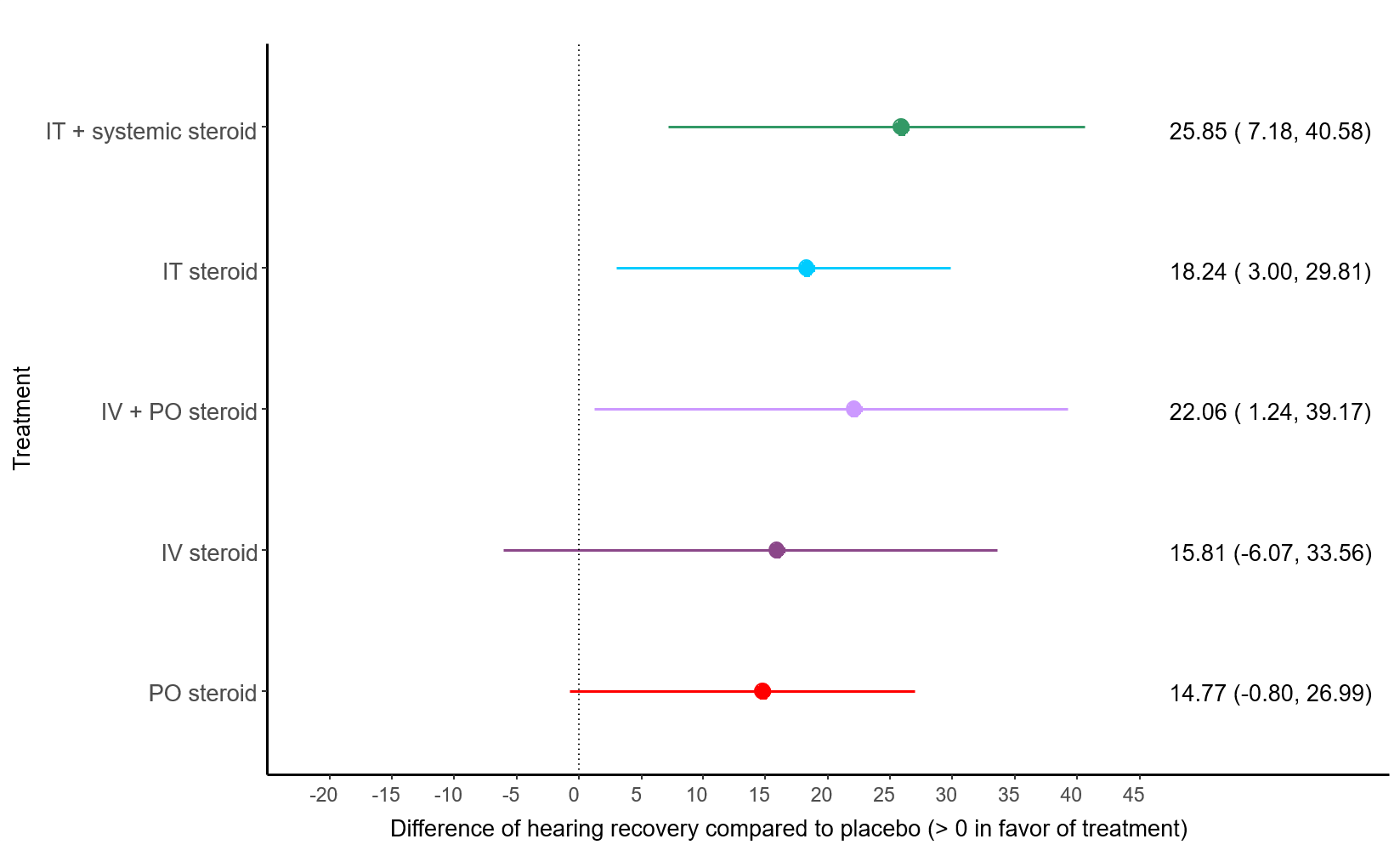


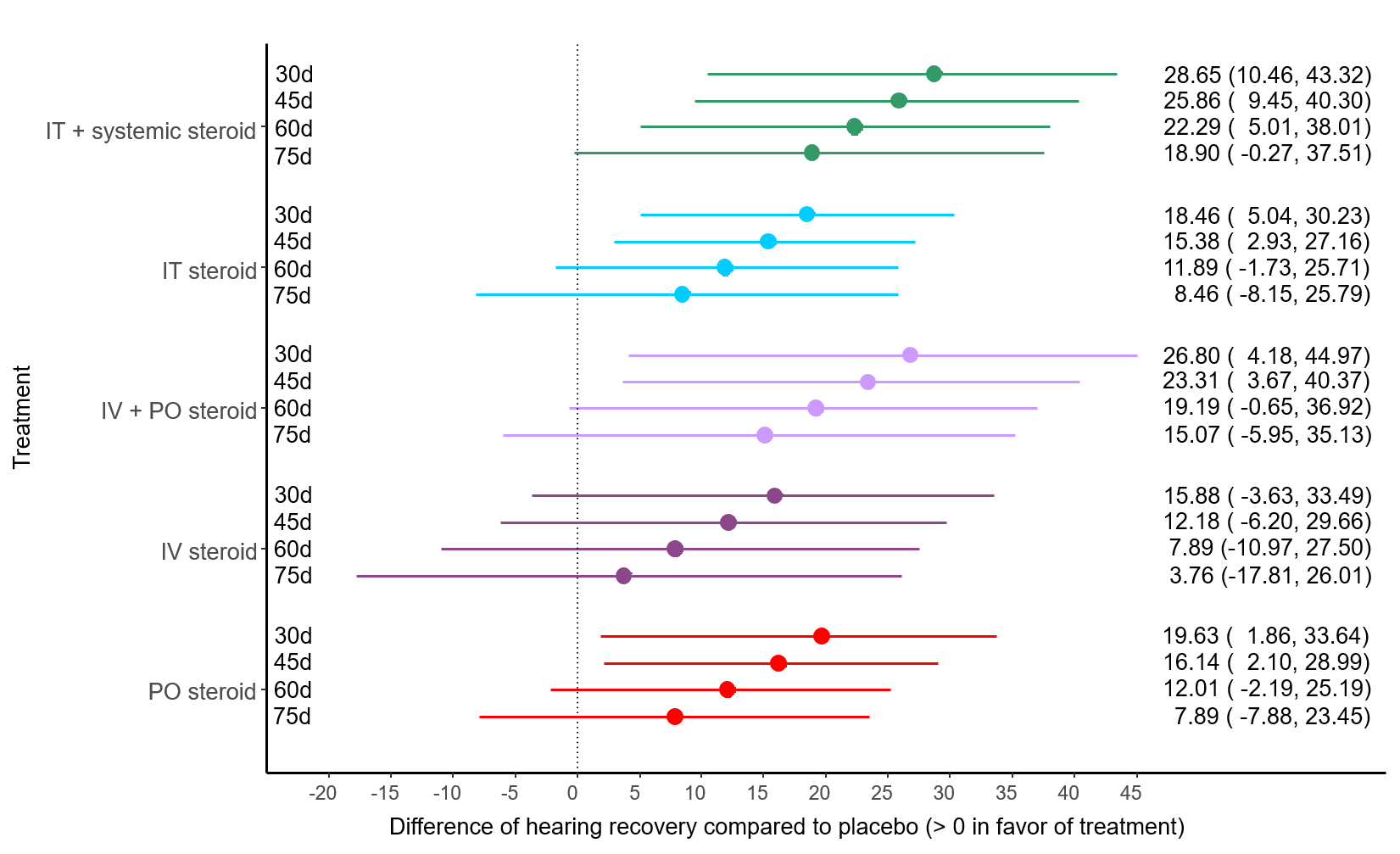
**

**Figure A:** Estimated difference of PTA improvement (dB) compared to placebo from the RE consistency model (with 95% credible intervals). Top: estimates from unadjusted NMA, bottom: estimates at the follow-up time of 30, 45, 60, 75 days from the time-adjusted model.

**Figure B**

**
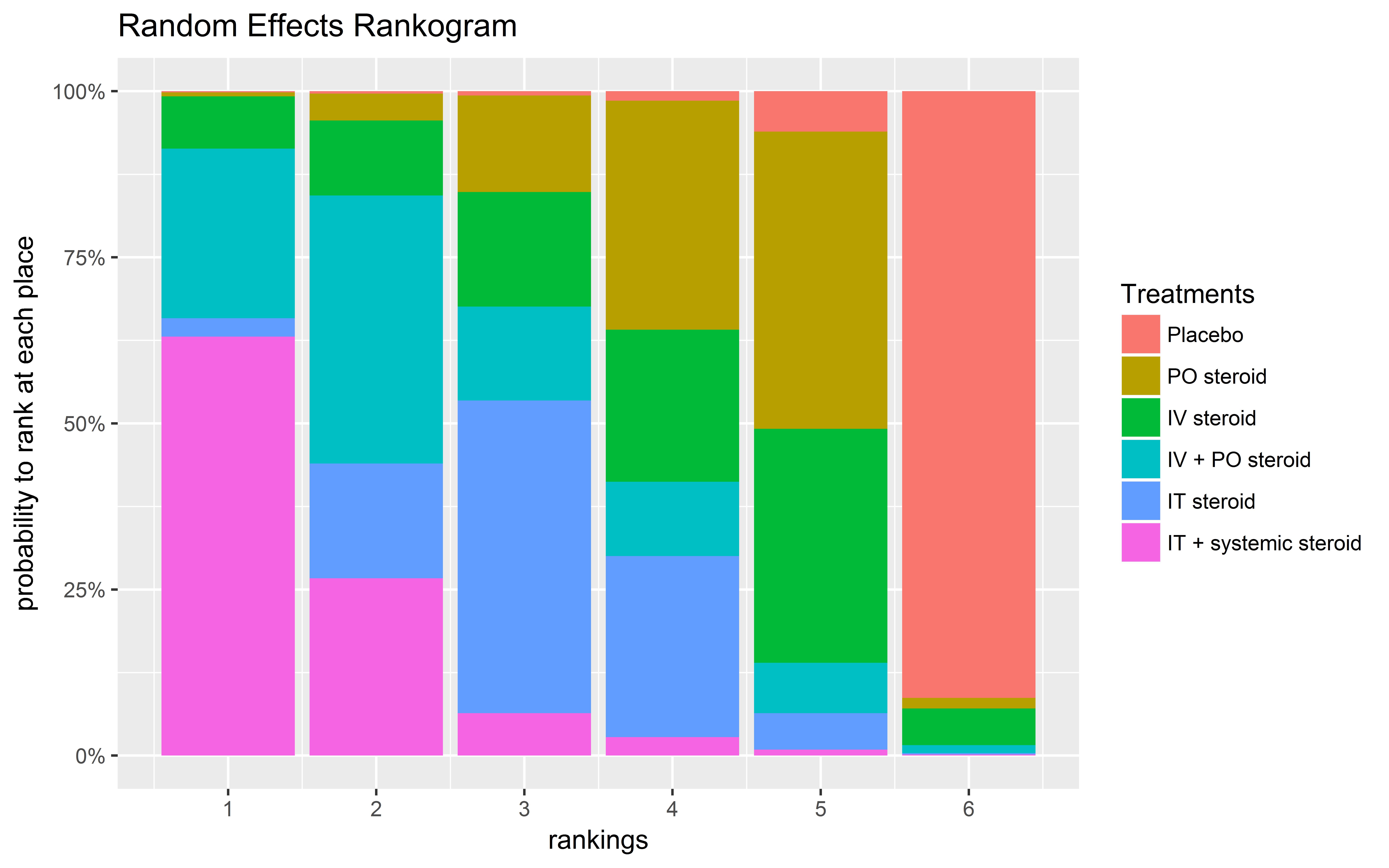

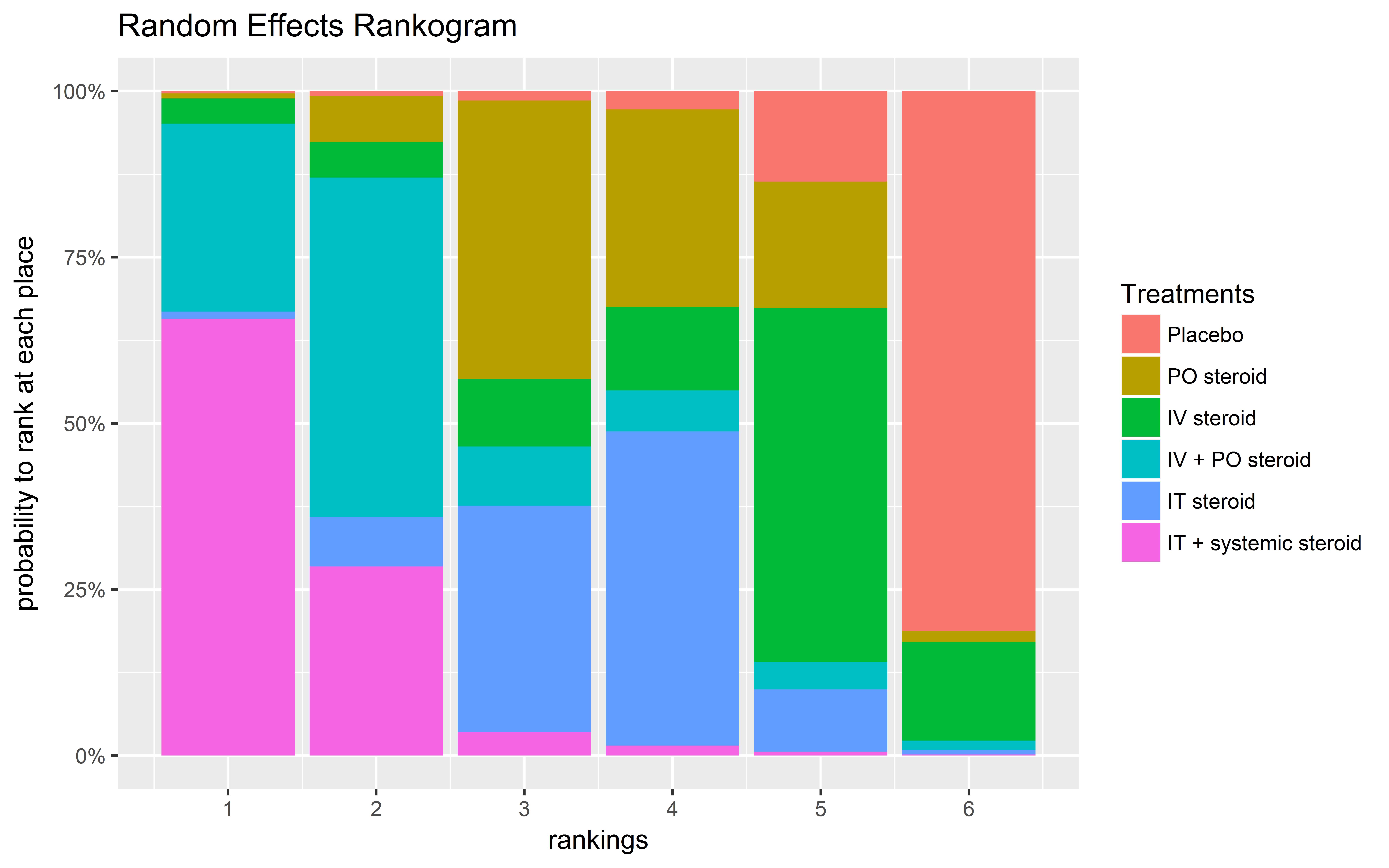
**

**Figure B:** The probability of treatments to be ranked at each place for PTA improvement from the RE consistency model (left: estimates from unadjusted NMA, right: estimates at the follow-up time of 60 days from the time-adjusted model).

**Figure C**

**
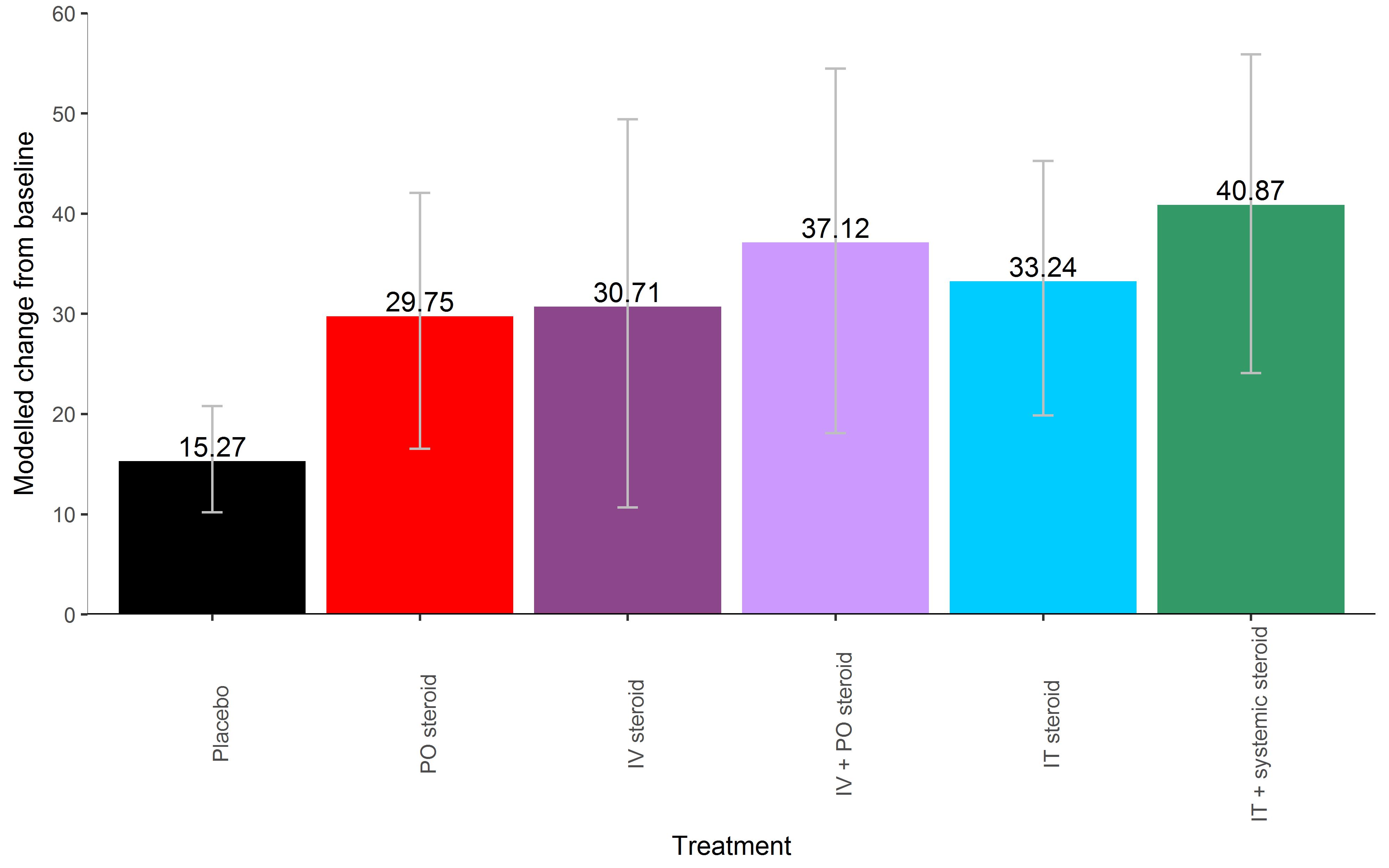

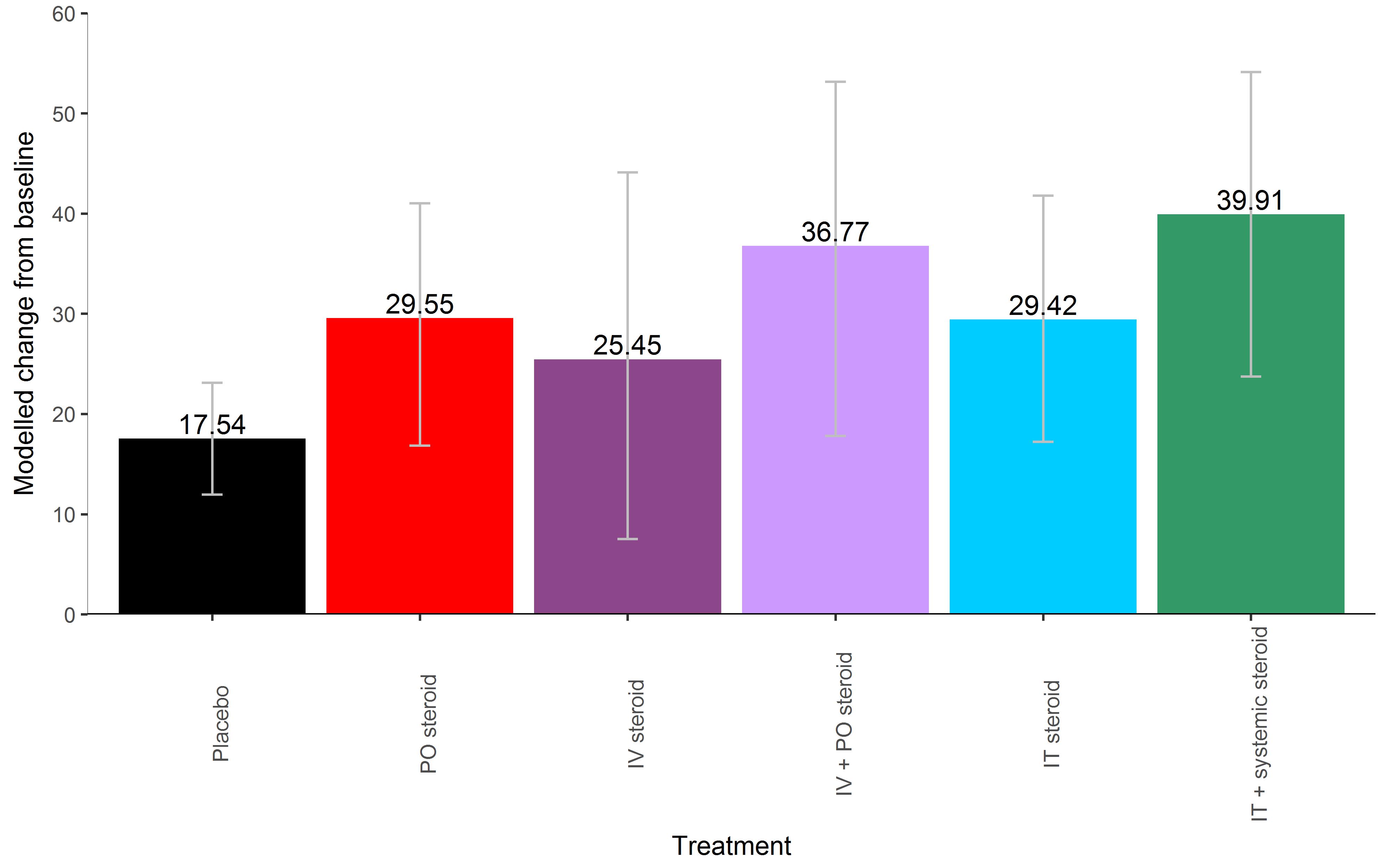
**

**Figure C:** Modelled PTA improvement (dB) from the RE consistency model with 95% CrIs (left: estimates from unadjusted NMA, right: estimates at the follow-up time of 60 days from the time-adjusted model).

**Figure D**

| **IT + systemic steroid** | 0.961 | 0.699 | 0.945 | 0.972 | **0.990** |
| --- | --- | --- | --- | --- | --- |
| 0.916 | **IT steroid** | 0.141 | 0.787 | 0.486 | 0.961 |
| 0.711 | 0.278 | **IV + PO steroid** | 0.885 | 0.874 | 0.972 |
| 0.886 | 0.672 | 0.764 | **IV steroid** | 0.269 | 0.840 |
| 0.971 | 0.819 | 0.859 | 0.555 | **PO steroid** | 0.959 |
| **0.993** | **0.987** | **0.980** | 0.938 | 0.970 | **Placebo** |

**Figure D:** League table of the pairwise probabilities that a treatment is better than another in PTA improvement from the unadjusted (lower triangle) and the time-adjusted models (estimated at the follow-up time of 60 days, upper triangle). For each comparison, the lower/right-most comparison for each comparison is the reference treatment. Shaded cells denote comparisons where the corresponding 95% credible interval excluded 1 (i.e. ruled out a null difference).

Figures E-H are from the binary outcome analyses (responders’ recovery and total recovery).

**Figure E**

**
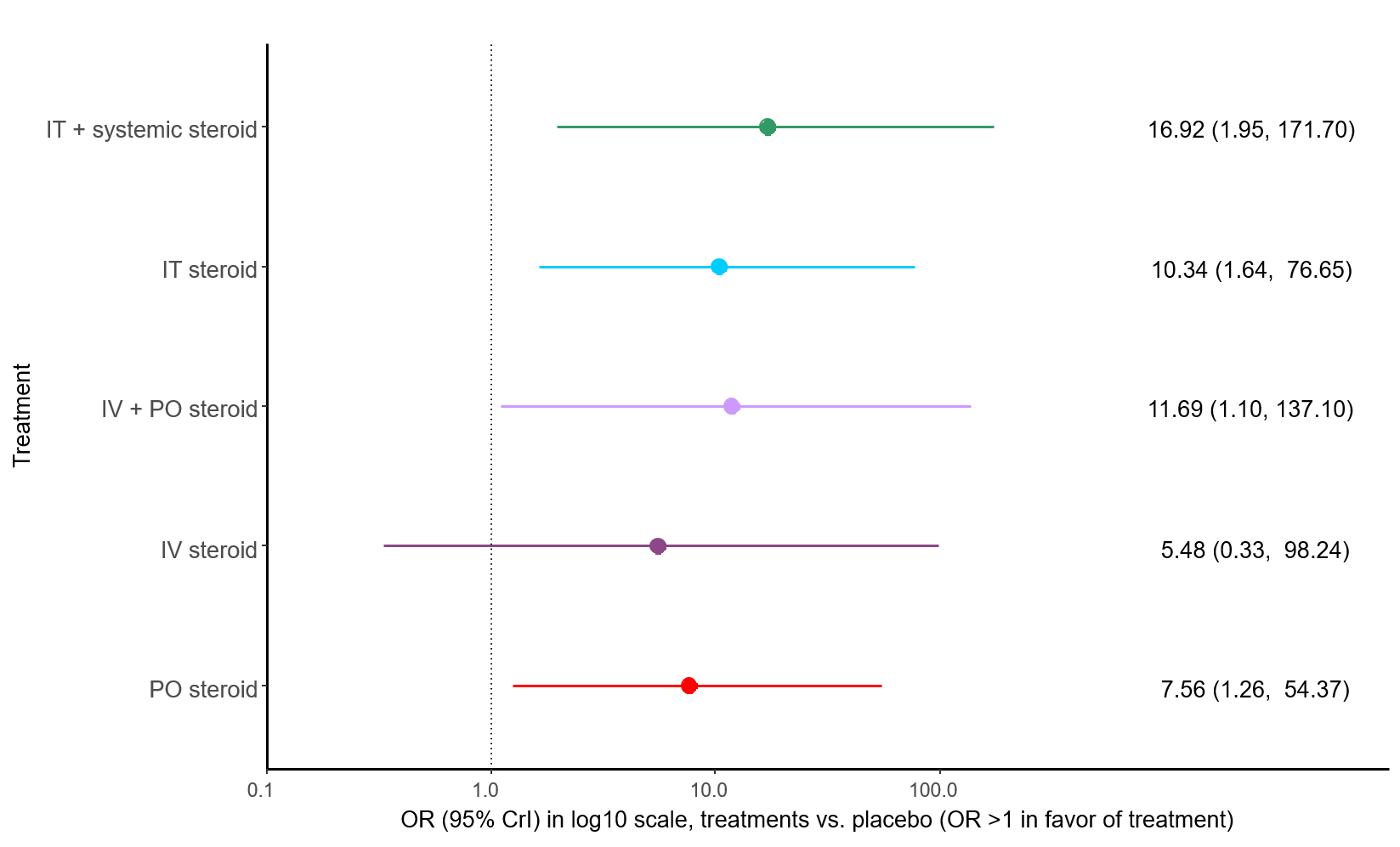


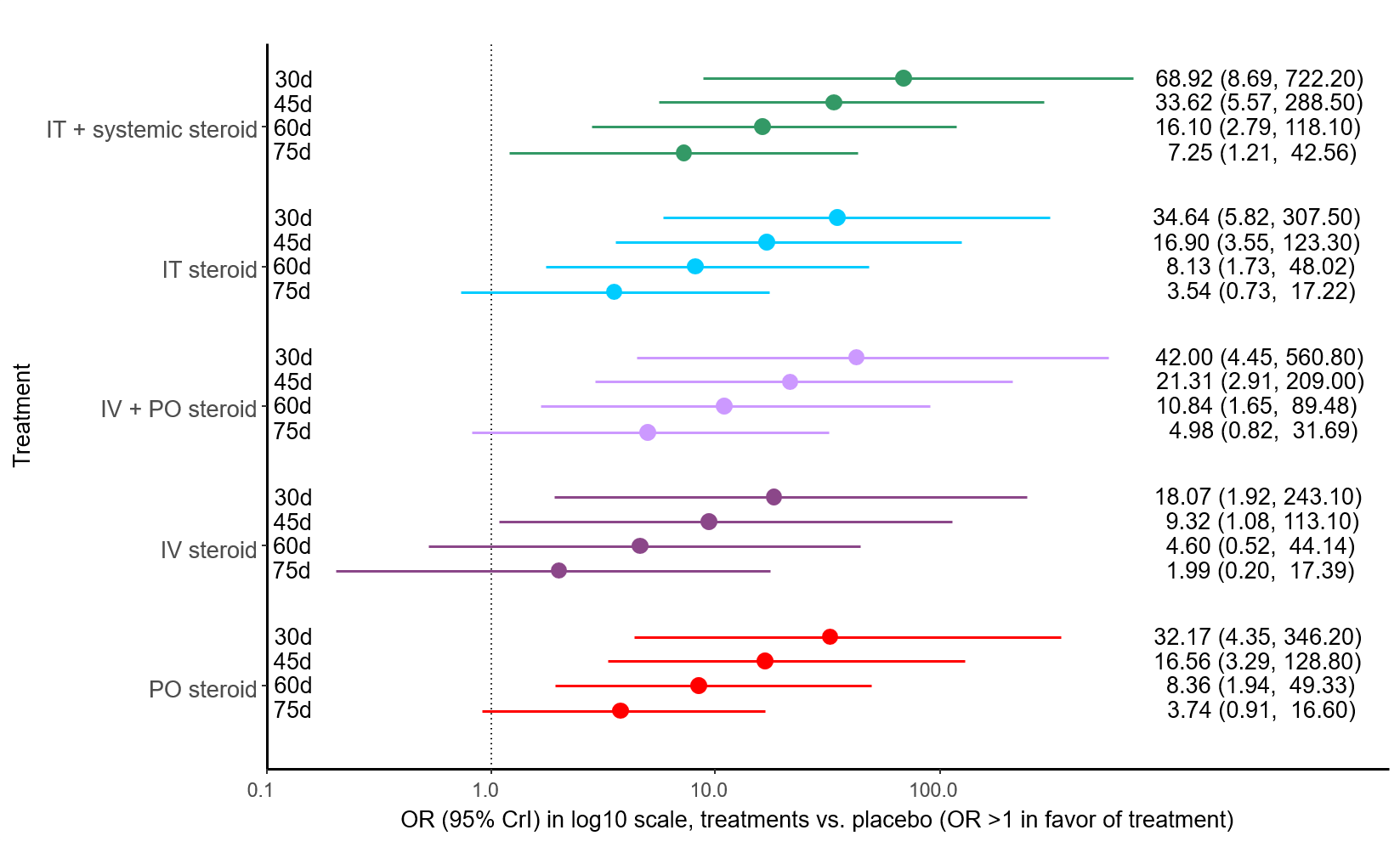

Figure E:** Estimated odds ratio compared to placebo for responders’ recovery from the RE consistency model (with 95% credible intervals). Top: estimates from unadjusted NMA, bottom: estimates at the follow-up time of 30, 45, 60, 75 days from the time-adjusted model.

**Figure F**
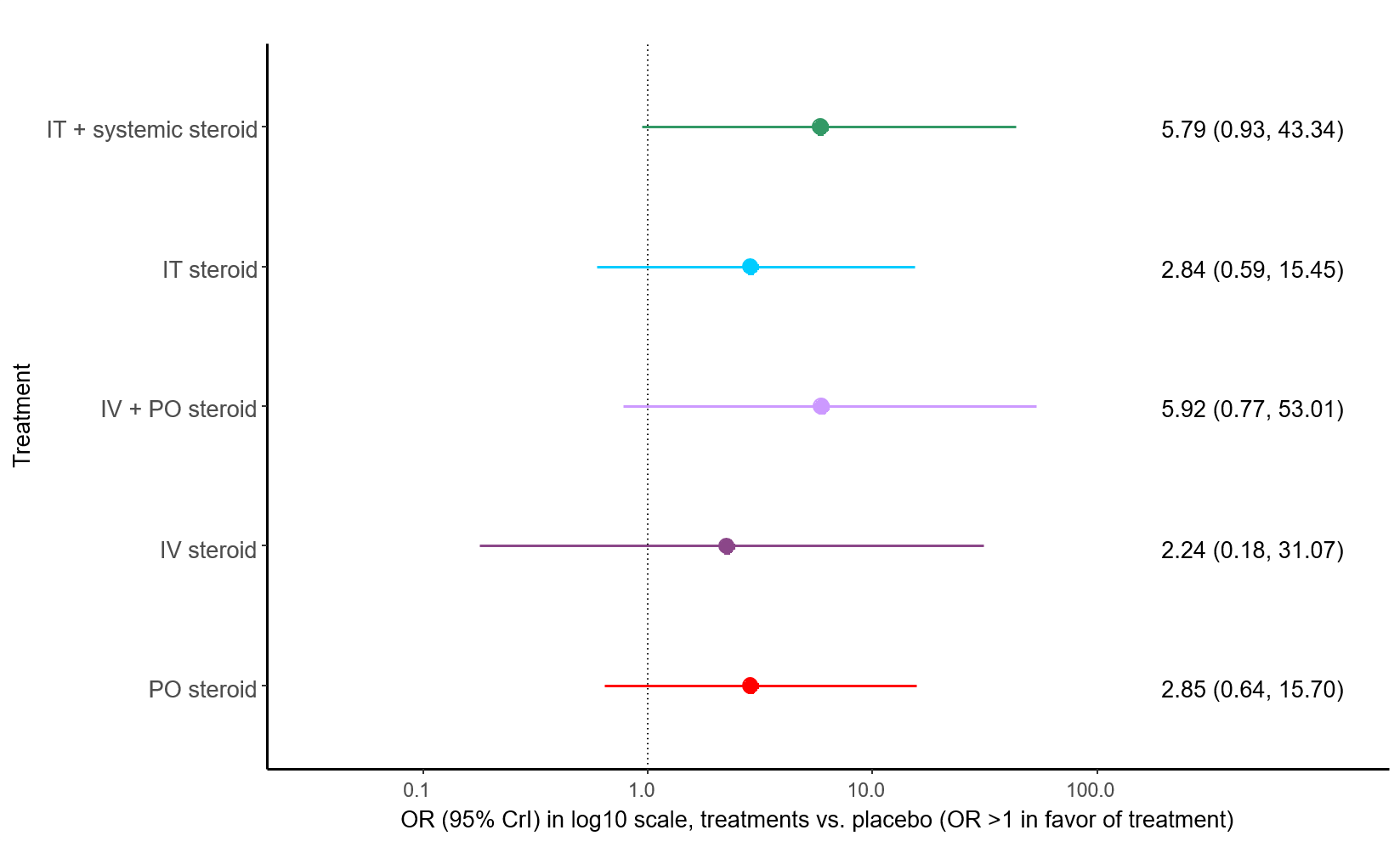

**
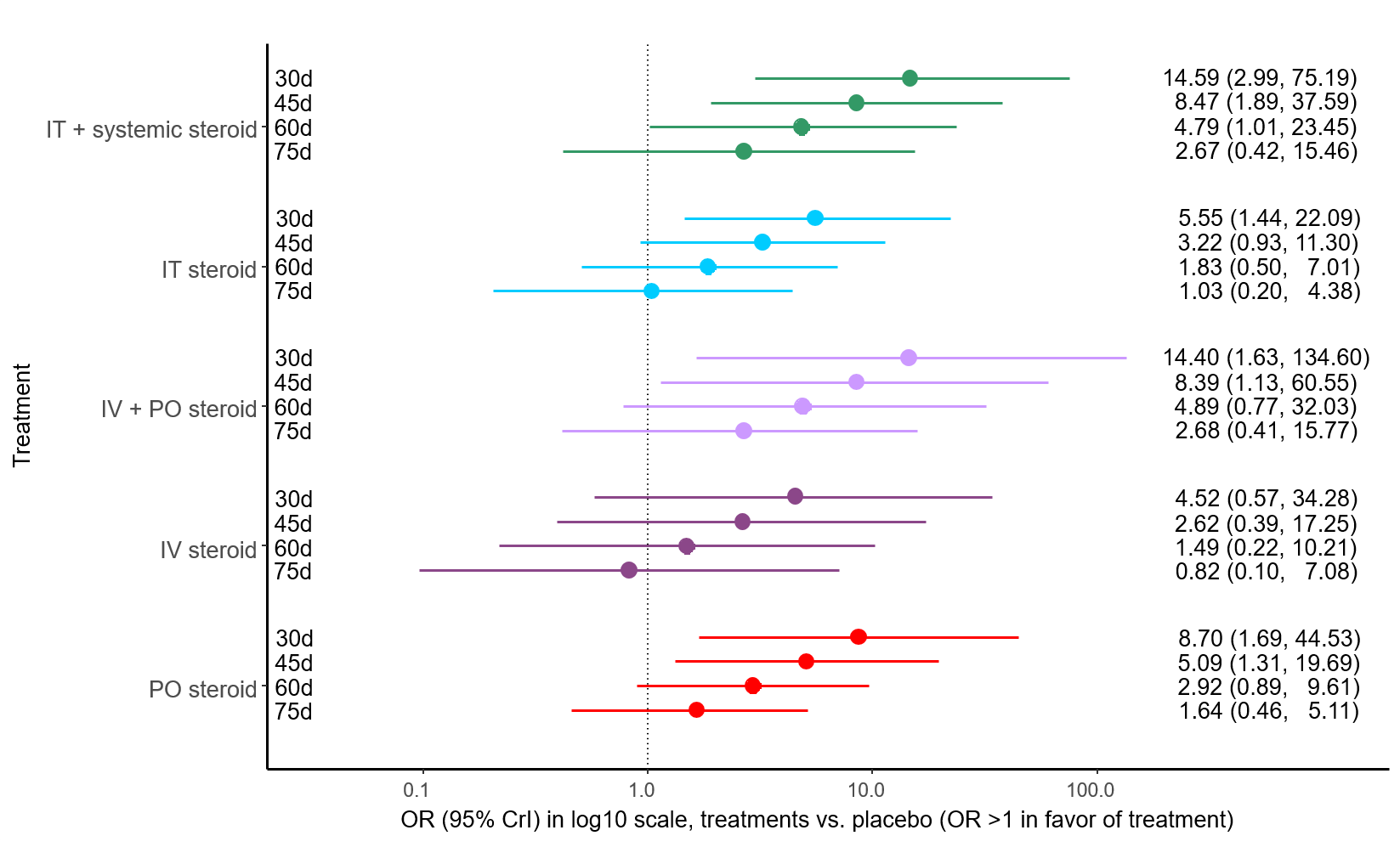
**

**Figure F:** Estimated odds ratio compared to placebo for total recovery from the RE consistency model (with 95% credible intervals). Top: estimates from unadjusted NMA, bottom: estimates at the follow-up time of 30, 45, 60, 75 days from the time-adjusted model.

**Figure G**

**
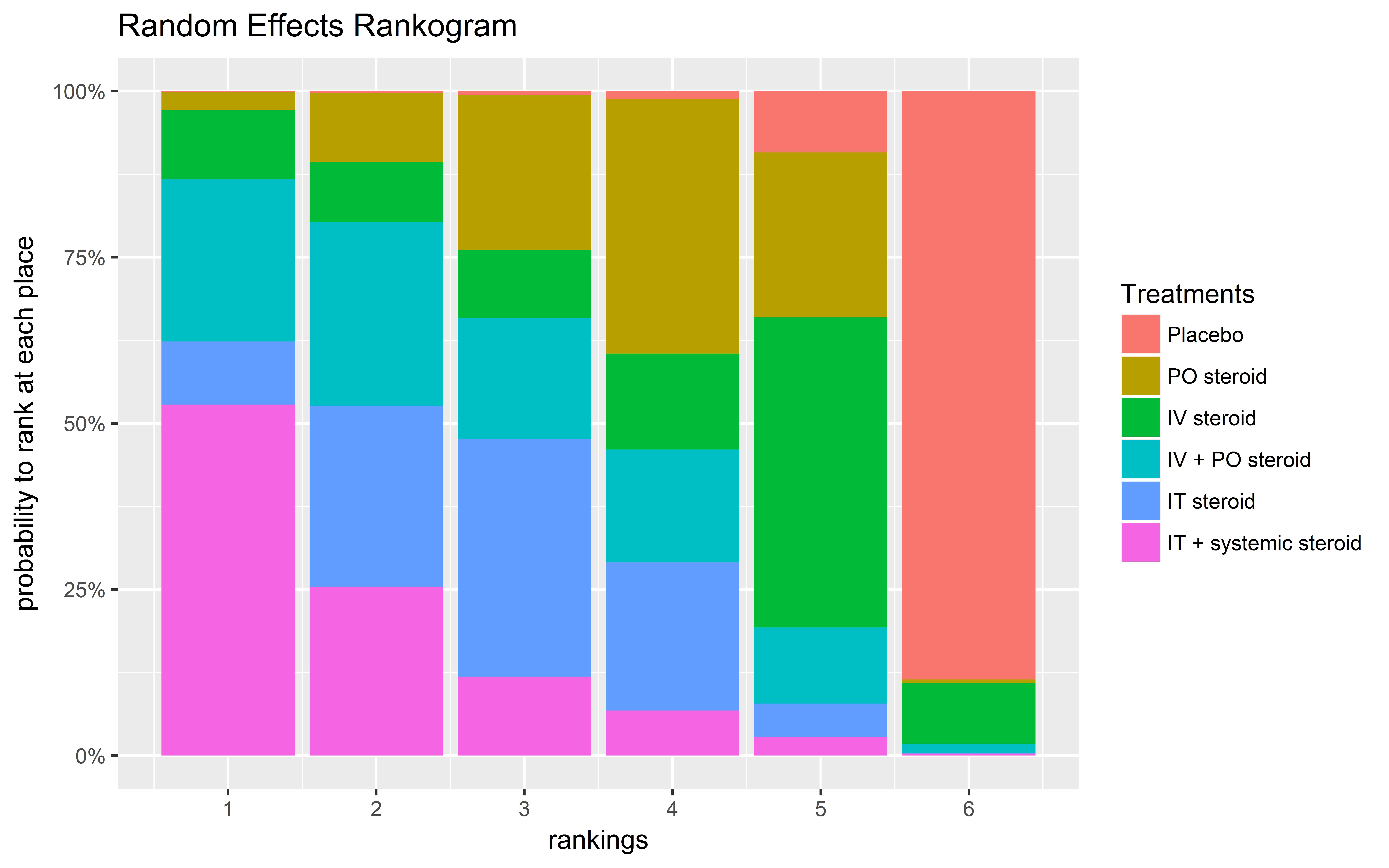

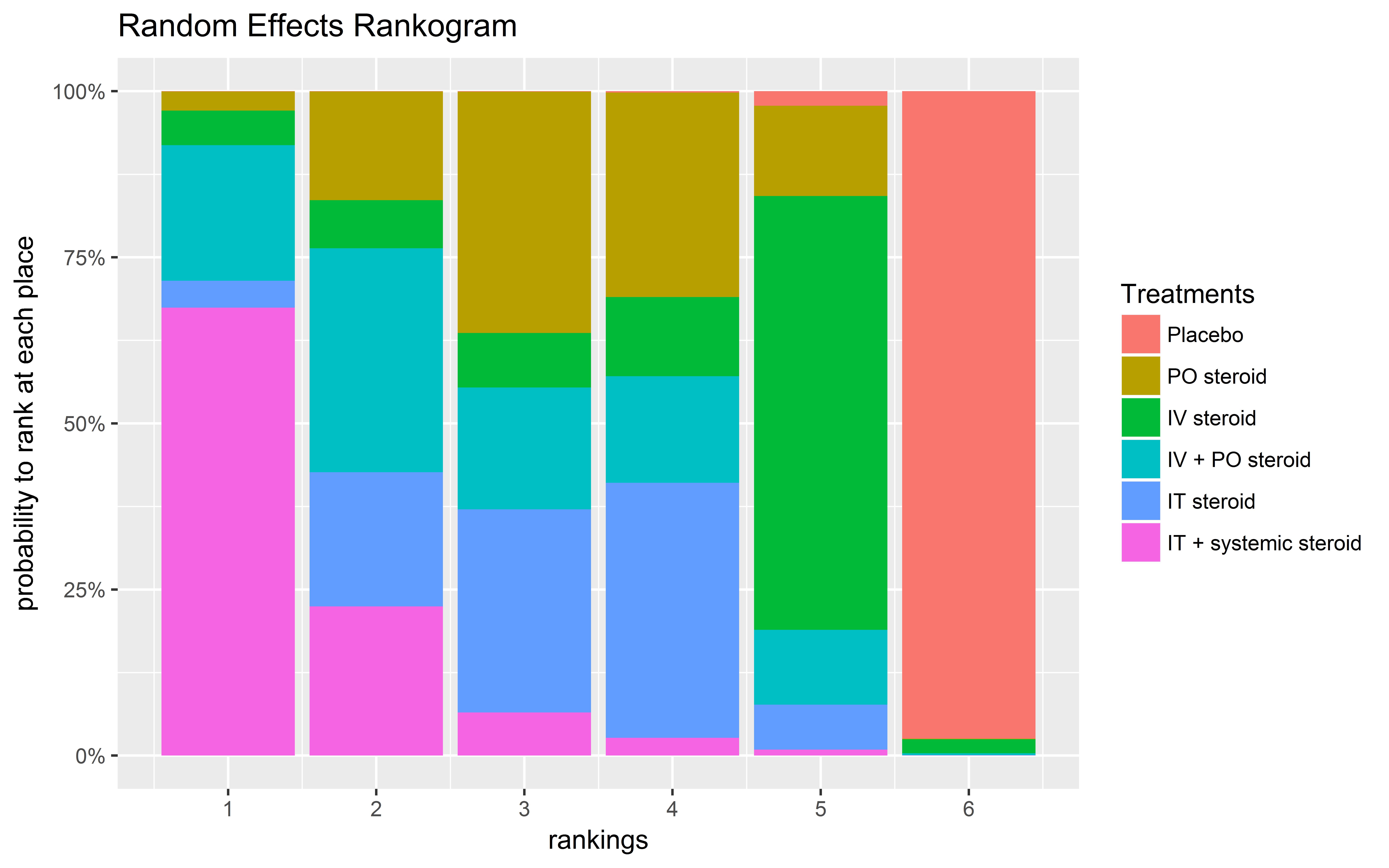

Figure G:** The probability of treatments to be ranked at each place for responders’ recovery from the RE consistency model (left: estimates from unadjusted NMA, right: estimates at the follow-up time of 60 days from the time-adjusted model).

**Figure H**

**
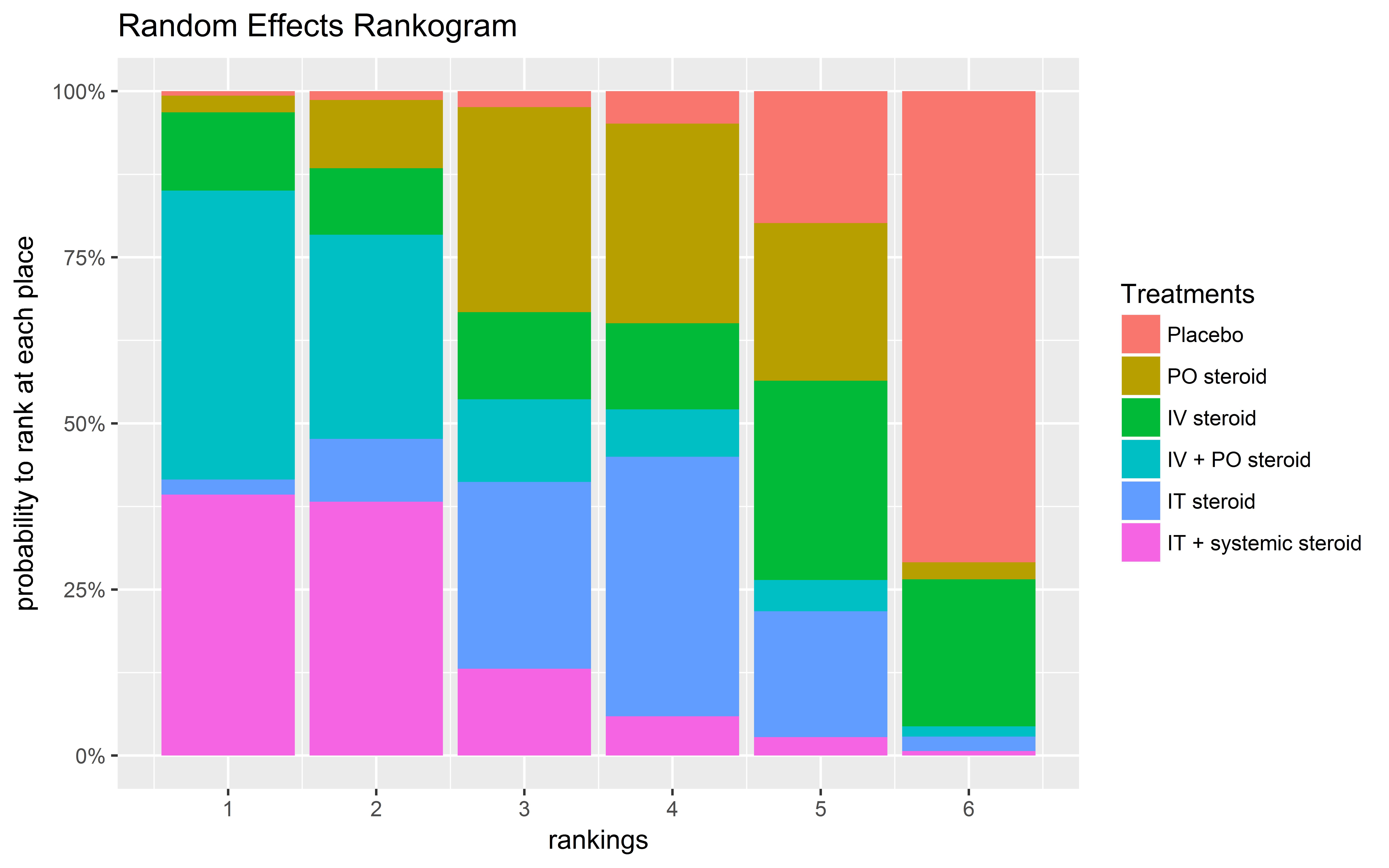

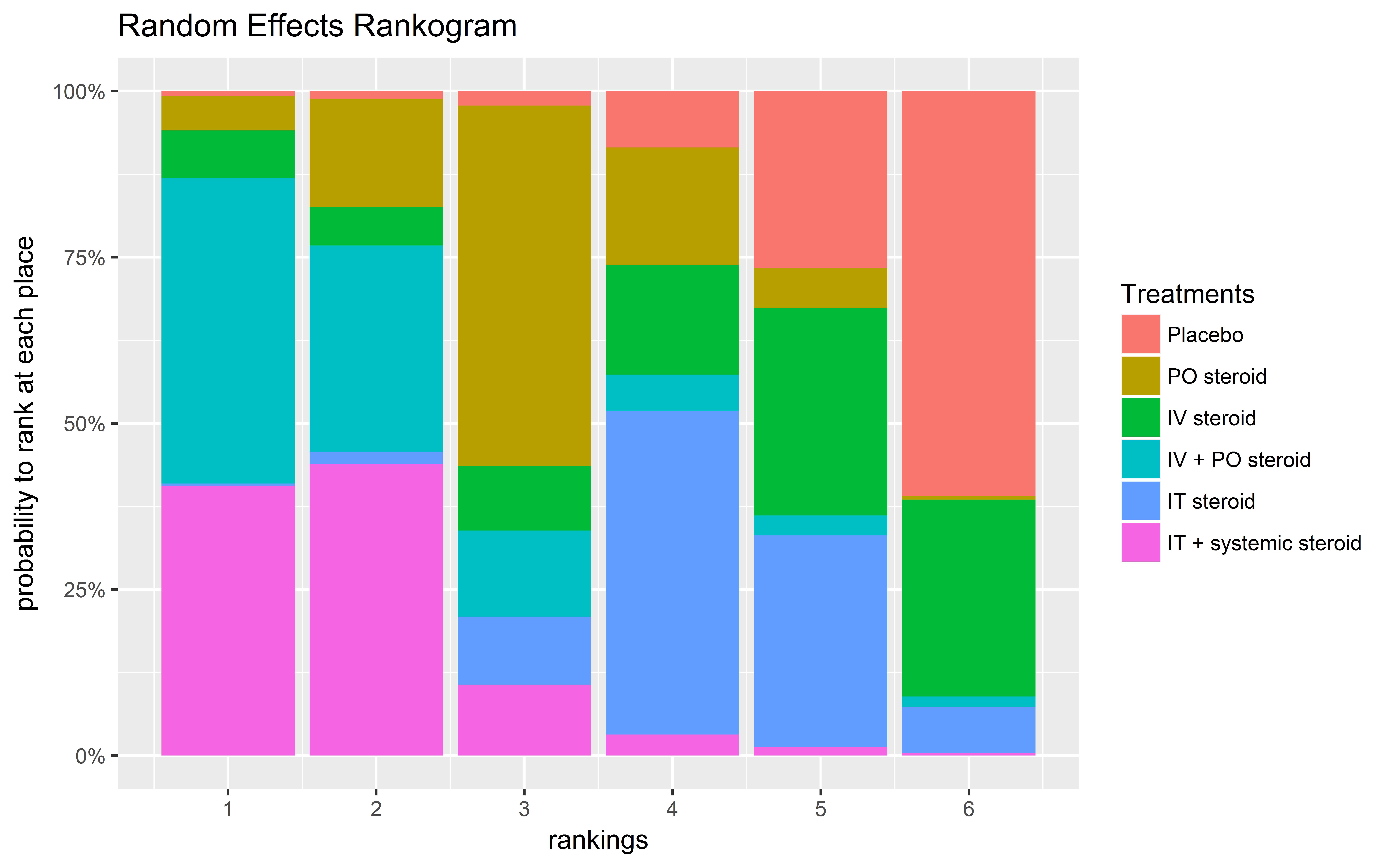
**

**Figure H:** The probability of treatments to be ranked at each place for total recovery from the RE consistency model. (left: estimates from unadjusted NMA, right: estimates at the follow-up time of 60 days from the time-adjusted model).

**The OpenBUGS code for PTA improvement modified upon the TSD-2 random effects model**

library(R2OpenBUGS)

# TSD-2 code modified to include both sources of data

# 1) mean PTA improvement from baseline and corresponding SEs per arm from 7 studies

# 2) PTA at the baseline and at the endpoint with the corresponding SDs from 2 studies

# Normal likelihood, identity link

# Random effects model for multi-arm trials

norm_ident_consist <- function(){ # *** PROGRAM STARTS

for (i in 1:ns){

for (k in 1:na[i]){

sesq[i,k] <- pow(se[i,k], 2) # calculate var for the improvement

}

}

for (i in (ns+1):(ns+ns2)){

for (k in 1:na[i]){

sesq[i,k] <- (pow(sd.bsl[i-ns,k], 2) + pow(sd.end[i-ns,k], 2)

- 2 * rho * sd.bsl[i-ns,k] * sd.end[i-ns,k])/N[i-ns,k]

} # calculate var for baseline – final

}

for(i in 1:(ns+ns2)){ # LOOP THROUGH STUDIES

w[i,1] <- 0

# adjustment for multi-arm trials is zero for control arm

delta[i,1] <- 0 # treatment effect is 0 for control arm

mu[i] ~ dnorm(0, 0.0001) # vague priors for all trial baselines

for (k in 1:na[i]){ # LOOP THROUGH ARMS

prec[i,k] <- 1/sesq[i,k] # set precisions

y[i,k] ~ dnorm(theta[i,k], prec[i,k]) # normal likelihood

theta[i,k] <- mu[i] + delta[i,k] # model for linear predictor

dev[i,k] <- (y[i,k]-theta[i,k])*(y[i,k]-theta[i,k])*prec[i,k] # Deviance contribution

}

resdev[i] <- sum(dev[i,1:na[i]]) # summed residual deviance contribution

for (k in 2:na[i]){ # LOOP THROUGH ARMS

delta[i,k] ~ dnorm(md[i,k], taud[i,k])

# trial-specific treatment effect distributions

md[i,k] <- d[t[i,k]] - d[t[i,1]] + sw[i,k]

# mean of treat effects distributions (with multi-arm trial correction)

taud[i,k] <- tau*2*(k-1)/k

# precision of treat effects distributions (with multi-arm trial correction)

w[i,k] <- delta[i,k] - d[t[i,k]] + d[t[i,1]] # adjustment for multi-arm RCTs

sw[i,k] <- sum(w[i,1:k-1])/(k-1) # cumulative adjustment for multi-arm trials

}

}

totresdev <- sum(resdev[]) # Total Residual Deviance

rho ~ dunif(0.5, 1)

d[1] <- 0 # treatment effect is 0 for ref trt

for (k in 2:nt){

d[k] ~ dnorm(0, 0.0001) # vague priors for treatment effects

}

sd ~ dunif(0, 20) # vague prior for between-trial SD

tau <- pow(sd, -2) # between-trial precision

# Output

# pairwise treatment effect for all possible pair-wise comparisons, if nt>2

for (c in 1:(nt-1)) {

for (k in (c+1):nt) {

Diff[c,k] <- d[k] - d[c]

better[c,k]<- step(Diff[c,k]) # assumes a positive result is "good"

}

}

# ranking on relative scale

for (k in 1:nt) {

rk[k] <- nt+1-rank(d[],k) # assumes events are "good"

best[k] <- equals(rk[k],1) # probability that treatment k is best

for (i in 1:nt){

prk[i,k] <- equals(rk[k],i) # prob of treatment k being each rank i

}

}

for (k in 1:nt) {

for (h in 1:nt){

cumeffectiveness[k,h] <- sum(prk[1:h,k])
 # The cumulative ranking probability of treatment k to be among the h best treatments.

}

SUCRA[k] <- sum(cumeffectiveness[k, 1:(nt-1)])/(nt-1)

# The surface under the cumulative rankings for treatment k.

}

# Provide estimates of effects T[k]

# Absolute effects with placebo treatment based on number of placebo controlled trials

for (i in 1:ns){

mu1[i] <- mu[i] * equals(t[i,1],1)

}

A <- sum(mu1[])/nt1

for (k in 1:nt) {

T[k] <- A + d[k]

}

} # *** PROGRAM ENDS
