# Supplementary material for: A systematic review and network meta-analysis of existing pharmacologic therapies in patients with idiopathic sudden sensorineural hearing loss
Source: PLoS One. 2019 Sep 9;14(9):e0221713. doi: 10.1371/journal.pone.0221713 (PMC6733451; doi:10.1371/journal.pone.0221713)

# S4 Text: NMAs Including Complementary Medicine Interventions

Figures A-C are from the PTA improvement analyses**.** Figures D-F are from the binary outcome analyses (responders’ recovery and total recovery).

**Figure A:** The league table of posterior median pairwise differences in PTA improvement from the unadjusted (lower triangle) and the time-adjusted models (estimated at the follow-up time of 60 days, upper triangle) based on NMAs including complementary medicine interventions, with credible intervals (2.5% and 97.5% quantiles). A complete summary of estimates for efficacy from the RE consistency model assuming vague priors is displayed. Statistically significant pairwise differences in PTA improvement are shown in bold, underlined font with grey shading. For each comparison, the lower/right-most treatment is the reference treatment.

| **IT + systemic steroid** | 10.58 (-1.18, 21.48) | 3.63 (-21.49, 28.23) | 3.17 (-10.37, 17.19) | 14.60 (-4.62, 32.86) | 9.95 (-10.24, 29.41) | 10.38 (-0.46, 20.41) | **22.19 (4.87, 37.77)** |
| --- | --- | --- | --- | --- | --- | --- | --- |
| 7.68 (-3.99, 18.83) | **IT steroid** | -6.97 (-28.08, 14.63) | -7.38 (-21.29, 7.72) | 4.02 (-9.83, 17.95) | -0.62 (-17.62, 16.50) | -0.19 (-9.36, 8.94) | 11.66 (-2.77, 25.06) |
| -0.59 (-26.14, 24.88) | -8.25 (-30.80, 14.74) | **IV steroid  + zinc** | -0.47 (-26.46, 26.70) | 10.97 (-5.67, 27.32) | 6.34 (-21.58, 34.09) | 6.76 (-17.40, 30.37) | 18.68 (-7.55, 43.19) |
| 3.67 (-10.84, 18.56) | -3.98 (-18.01, 10.83) | 4.29 (-22.34, 31.35) | **IV + PO steroid** | 11.40 (-10.17, 31.69) | 6.75 (-15.28, 27.71) | 7.18 (-6.93, 20.06) | 18.97 (-0.91, 36.73) |
| 10.36 (-8.12, 28.55) | 2.66 (-11.78, 17.26) | 10.89 (-6.91, 28.67) | 6.65 (-14.10, 26.79) | **IV steroid** | -4.64 (-27.18, 18.16) | -4.21 (-21.69, 13.13) | 7.70 (-12.39, 26.52) |
| 8.59 (-12.90, 29.56) | 0.91 (-17.02, 18.88) | 9.20 (-20.11, 37.90) | 4.89 (-18.48, 27.47) | -1.74 (-24.88, 21.10) | **PO steroid + vasodil + ginkgo** | 0.44 (-18.30, 18.88) | 12.22 (-10.33, 33.62) |
| 11.09 (-0.39, 21.89) | 3.42 (-5.31, 11.93) | 11.70 (-12.97, 35.70) | 7.39 (-7.38, 21.24) | 0.78 (-16.37, 17.32) | 2.53 (-17.40, 22.32) | **PO steroid** | 11.84 (-2.30, 25.05) |
| **25.75 (7.33, 40.26)** | **18.14 (3.11, 29.55)** | 26.43 (-1.86, 50.42) | **22.05 (1.26, 38.76)** | 15.56 (-6.01, 32.84) | 17.14 (-6.76, 37.62) | 14.68 (-0.55, 26.73) | **Placebo** |

**Figure B**

**
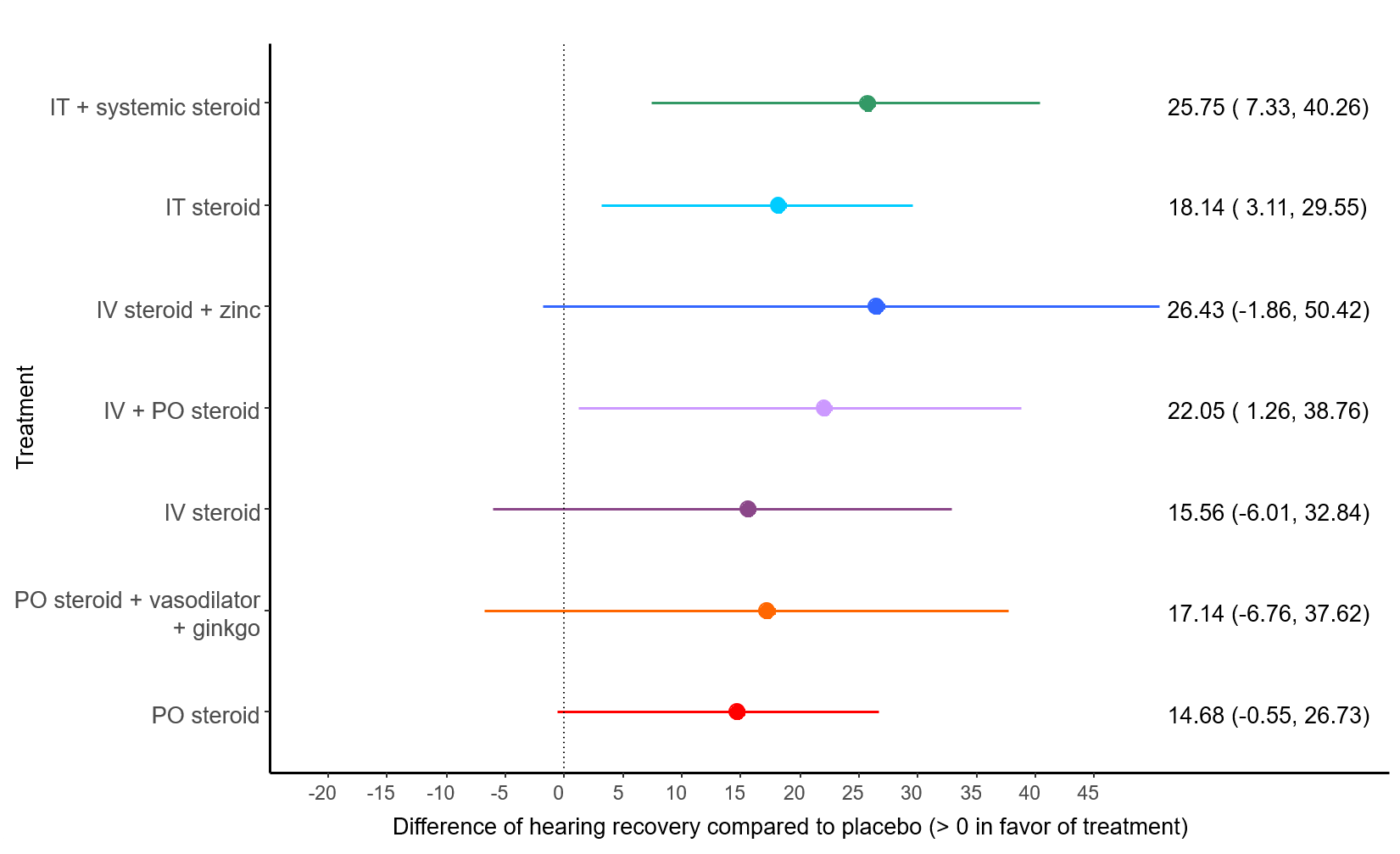


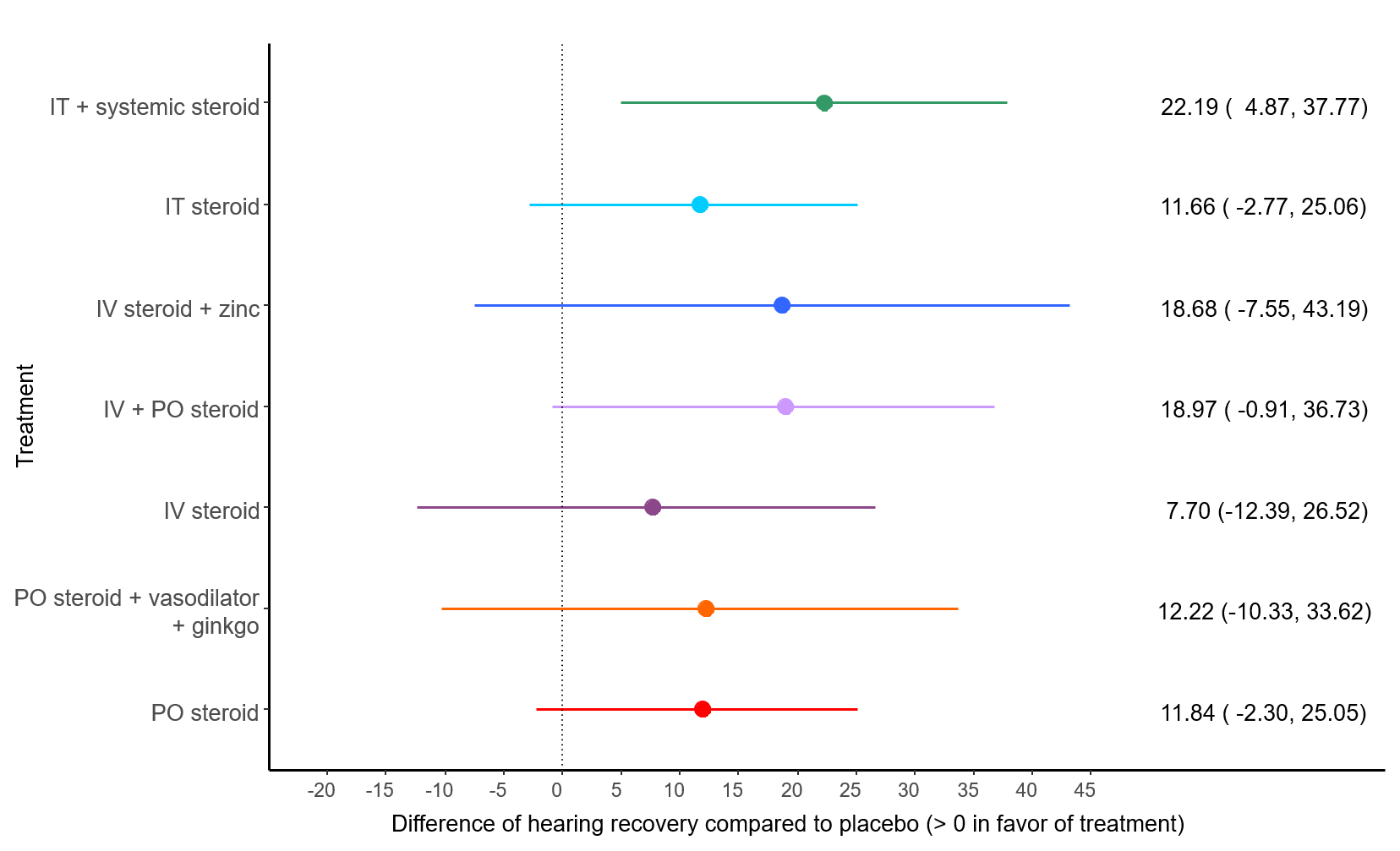
**

**Figure B:** Estimated difference of PTA improvement (dB) compared to placebo from the RE consistency model (with 95% credible intervals) based on NMAs including complementary medicine interventions. Top: estimates from unadjusted NMA, bottom: estimates at the follow-up time of 60 days from the time-adjusted model.

**Figure C**

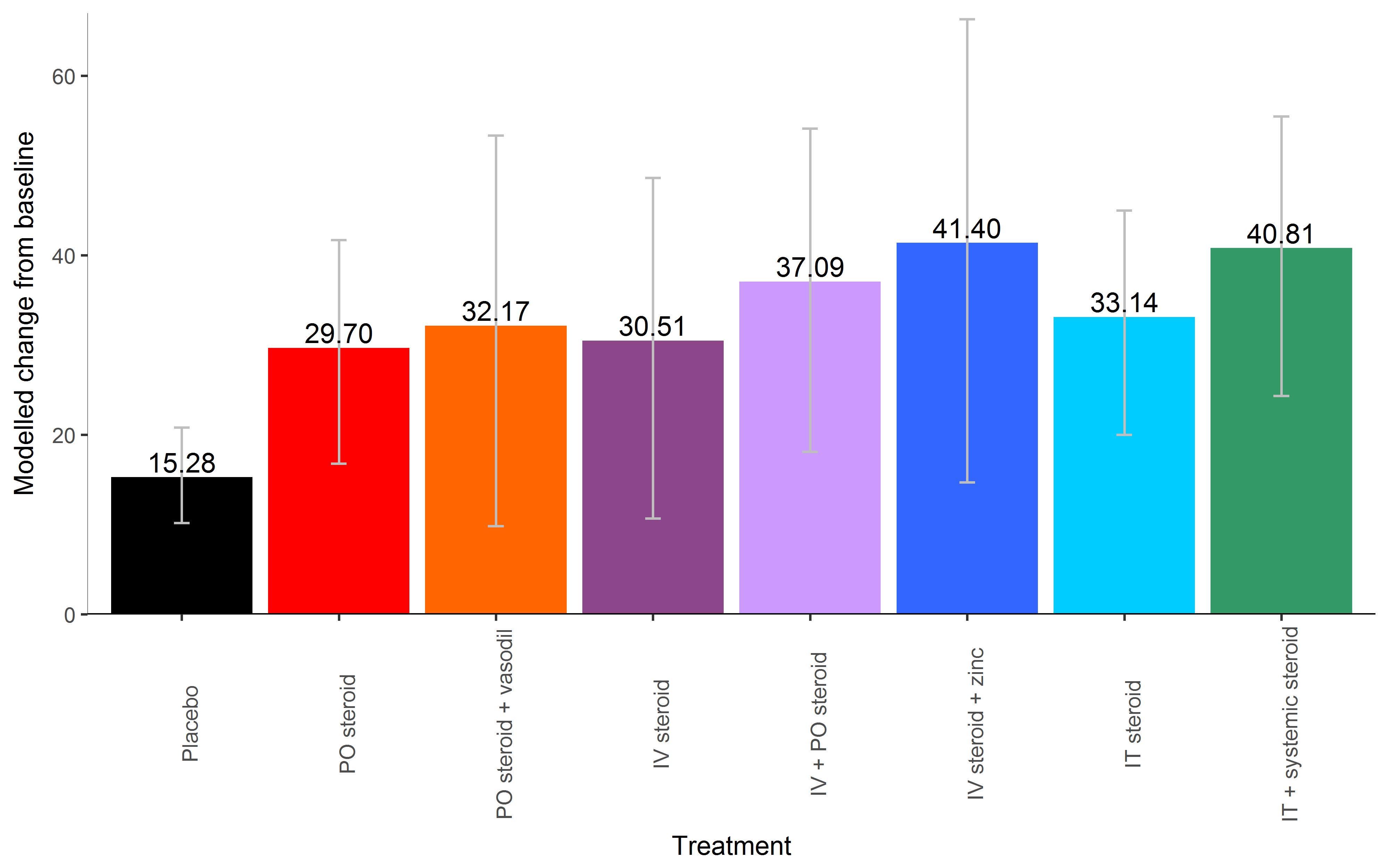


**
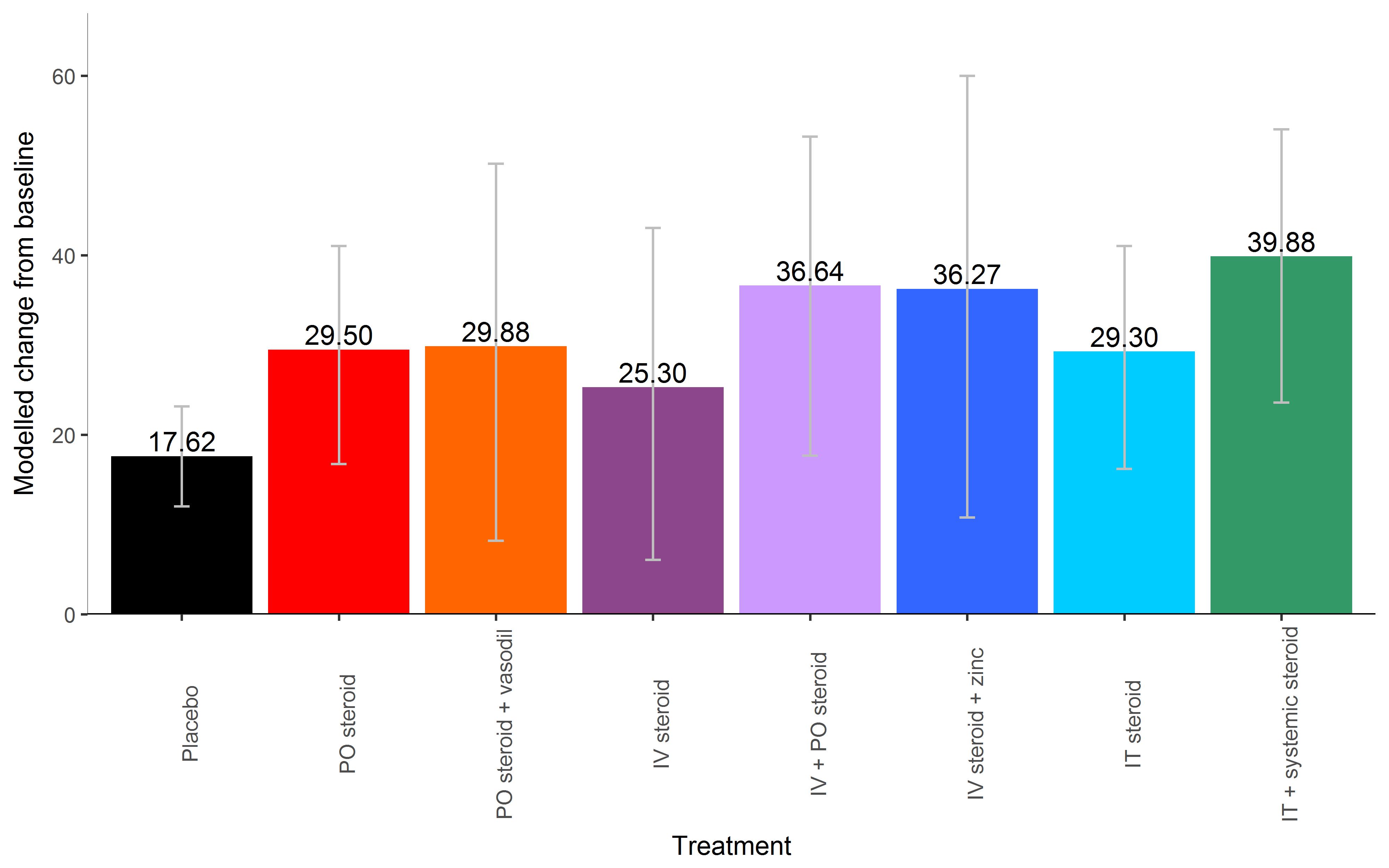
**

**Figure C:** Modelled PTA improvement (dB) from the RE consistency model with 95% CrIs based on NMAs including complementary medicine interventions (top: estimates from unadjusted NMA, bottom: estimates at the follow-up time of 60 days from the time-adjusted model).

**Figure D:** League tables of posterior median odds ratio in responders’ recovery / total recovery from the unadjusted (lower triangle) and the time-adjusted models (upper triangle) based on NMAs including complementary medicine interventions, with credible intervals (2.5% and 97.5% quantiles). A complete summary of estimates for efficacy from the RE consistency model assuming vague priors is displayed. For each comparison, the lower/right-most treatment is the reference treatment. Statistically significant pairwise odds ratio estimates are shown in bold, underlined font with grey shading. **Panel A:** responders’ recovery, **Panel B:** total recovery.

A: Responders’ recovery

| **IT + systemic steroid** | 1.97 (0.66, 5.99) | 1.46 (0.41, 5.18) | 3.44 (0.51, 24.99) | 2.49 (0.35, 19.34) | 1.92 (0.69, 5.23) | **15.73 (2.80, 92.95)** |
| --- | --- | --- | --- | --- | --- | --- |
| 1.65 (0.37, 7.24) | **IT steroid** | 0.73 (0.20, 2.70) | 1.75 (0.40, 8.36) | 1.27 (0.22, 7.69) | 0.98 (0.39, 2.35) | **7.79 (1.78, 39.98)** |
| 1.46 (0.22, 9.62) | 0.89 (0.15, 5.20) | **IV + PO steroid** | 2.37 (0.30, 19.78) | 1.72 (0.22, 14.30) | 1.34 (0.40, 4.14) | **10.86 (1.68, 75.59)** |
| 3.11 (0.24, 42.16) | 1.88 (0.23, 16.39) | 2.13 (0.14, 33.91) | **IV steroid** | 0.74 (0.07, 7.86) | 0.57 (0.09, 3.22) | 4.56 (0.53, 37.03) |
| 2.12 (0.12, 39.21) | 1.29 (0.11, 15.67) | 1.46 (0.07, 31.31) | 0.69 (0.03, 18.45) | **PO steroid + vasodil + ginkgo** | 0.77 (0.10, 4.95) | 6.23 (0.58, 64.66) |
| 2.25 (0.53, 9.19) | 1.37 (0.43, 4.23) | 1.54 (0.27, 8.37) | 0.73 (0.06, 7.86) | 1.06 (0.07, 15.08) | **PO steroid** | **7.95 (1.95, 40.73)** |
| **17.25 (1.92, 171.60)** | **10.45 (1.66, 77.01)** | **11.82 (1.13, 142.70)** | 5.56 (0.33, 99.91) | 8.08 (0.38, 184.30) | **7.66 (1.27, 54.60)** | **Placebo** |

B: Total recovery

| **IT + systemic steroid** | 2.63 (0.92, 7.69) | 0.98 (0.30, 3.34) | 3.06 (0.41, 26.22) | 2.97 (0.46, 20.26) | 1.62 (0.61, 4.50) | 4.79 (1.01, 23.85) |
| --- | --- | --- | --- | --- | --- | --- |
| 2.03 (0.54, 8.27) | **IT steroid** | 0.38 (0.10, 1.40) | 1.17 (0.24, 6.42) | 1.14 (0.21, 6.14) | 0.62 (0.25, 1.56) | 1.83 (0.50, 6.88) |
| 0.98 (0.19, 5.31) | 0.48 (0.09, 2.44) | **IV + PO steroid** | 3.14 (0.32, 33.50) | 3.06 (0.43, 21.92) | 1.66 (0.44, 6.36) | 4.87 (0.78, 31.75) |
| 2.56 (0.23, 30.45) | 1.26 (0.17, 9.50) | 2.61 (0.20, 35.63) | **IV steroid** | 0.97 (0.07, 11.74) | 0.52 (0.08, 3.31) | 1.55 (0.22, 10.65) |
| 2.29 (0.19, 31.08) | 1.13 (0.13, 9.93) | 2.35 (0.16, 35.55) | 0.90 (0.05, 17.46) | **PO steroid + vasodil + ginkgo** | 0.54 (0.08, 3.85) | 1.61 (0.17, 15.74) |
| 2.04 (0.56, 7.37) | 1.00 (0.31, 2.96) | 2.09 (0.42, 9.67) | 0.80 (0.08, 7.60) | 0.89 (0.07, 9.67) | **PO steroid** | 2.93 (0.89, 9.90) |
| 5.77 (0.95, 43.48) | 2.86 (0.60, 15.30) | 5.90 (0.78, 51.99) | 2.27 (0.18, 31.06) | 2.55 (0.18, 39.42) | 2.84 (0.64, 15.71) | **Placebo** |

**Figure E:** Estimated odds ratio compared to placebo for responders’ recovery from the RE consistency model (with 95% credible intervals) based on NMAs including complementary medicine interventions. Top: estimates from unadjusted NMA, bottom: estimates at the follow-up time of 60 days from the time-adjusted model.


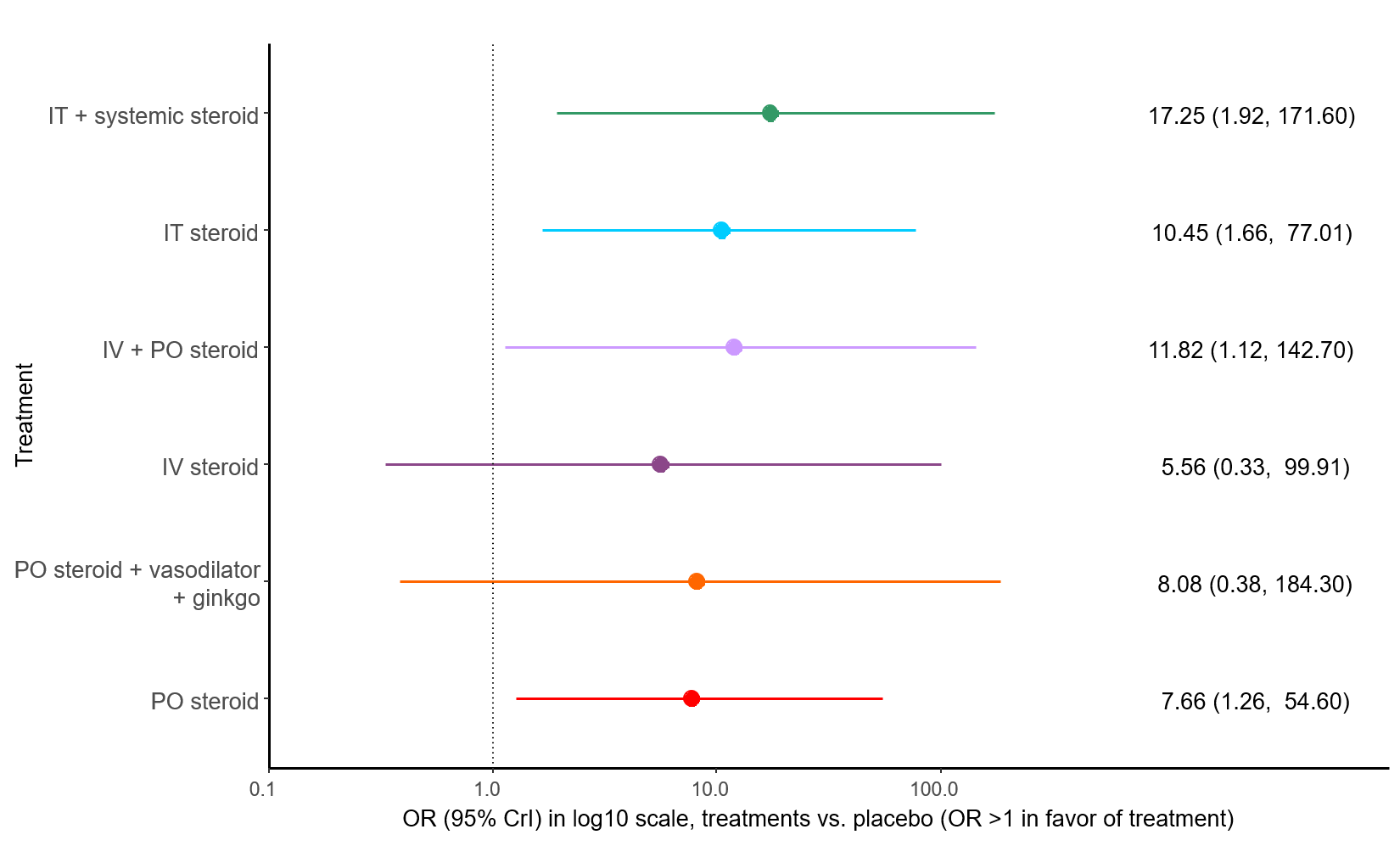


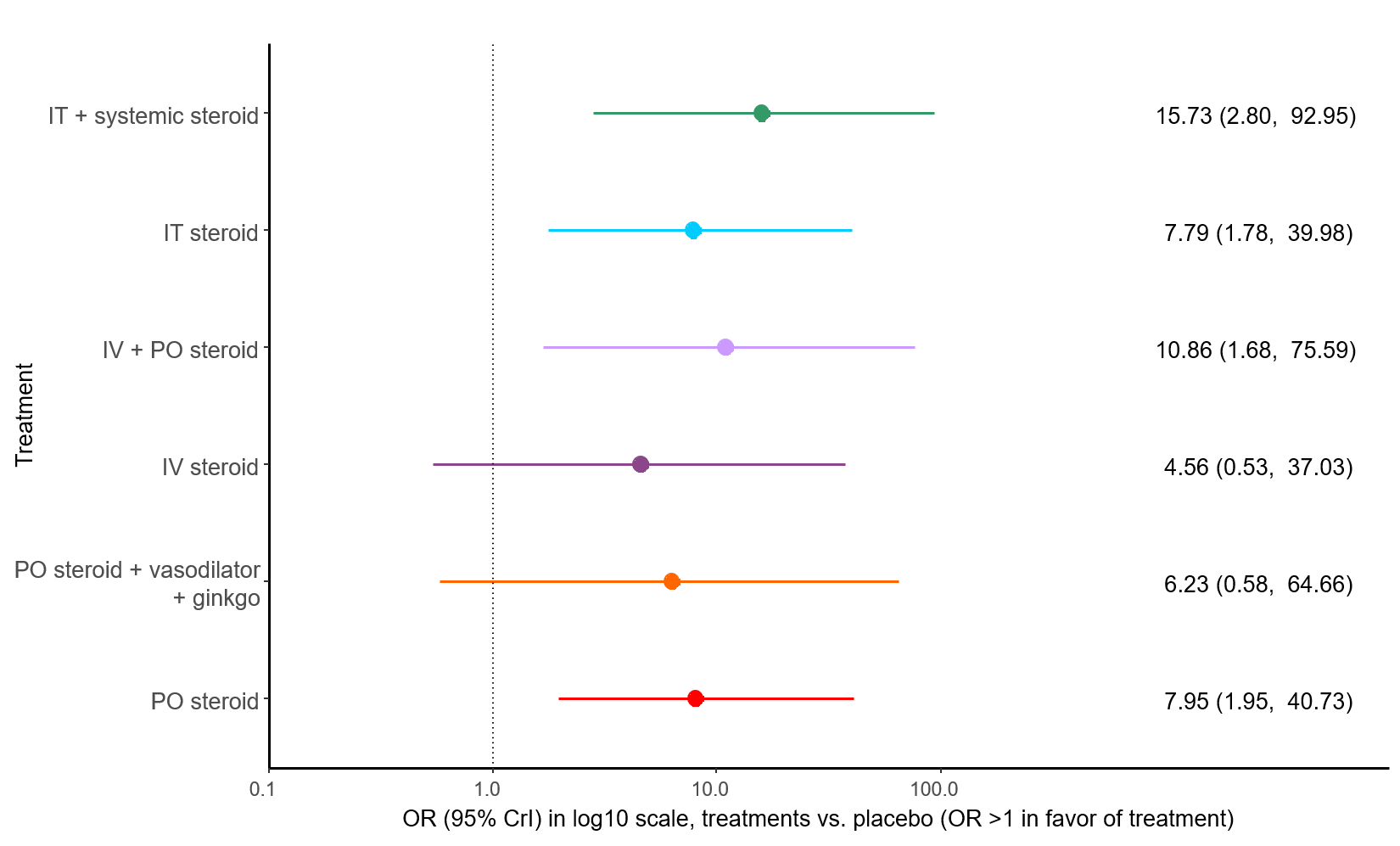


**Figure F:** Estimated odds ratio compared to placebo for total recovery from the RE consistency model (with 95% credible intervals) based on NMAs including complementary medicine interventions. Top: estimates from unadjusted NMA, bottom: estimates at the follow-up time of 60 days from the time-adjusted model.


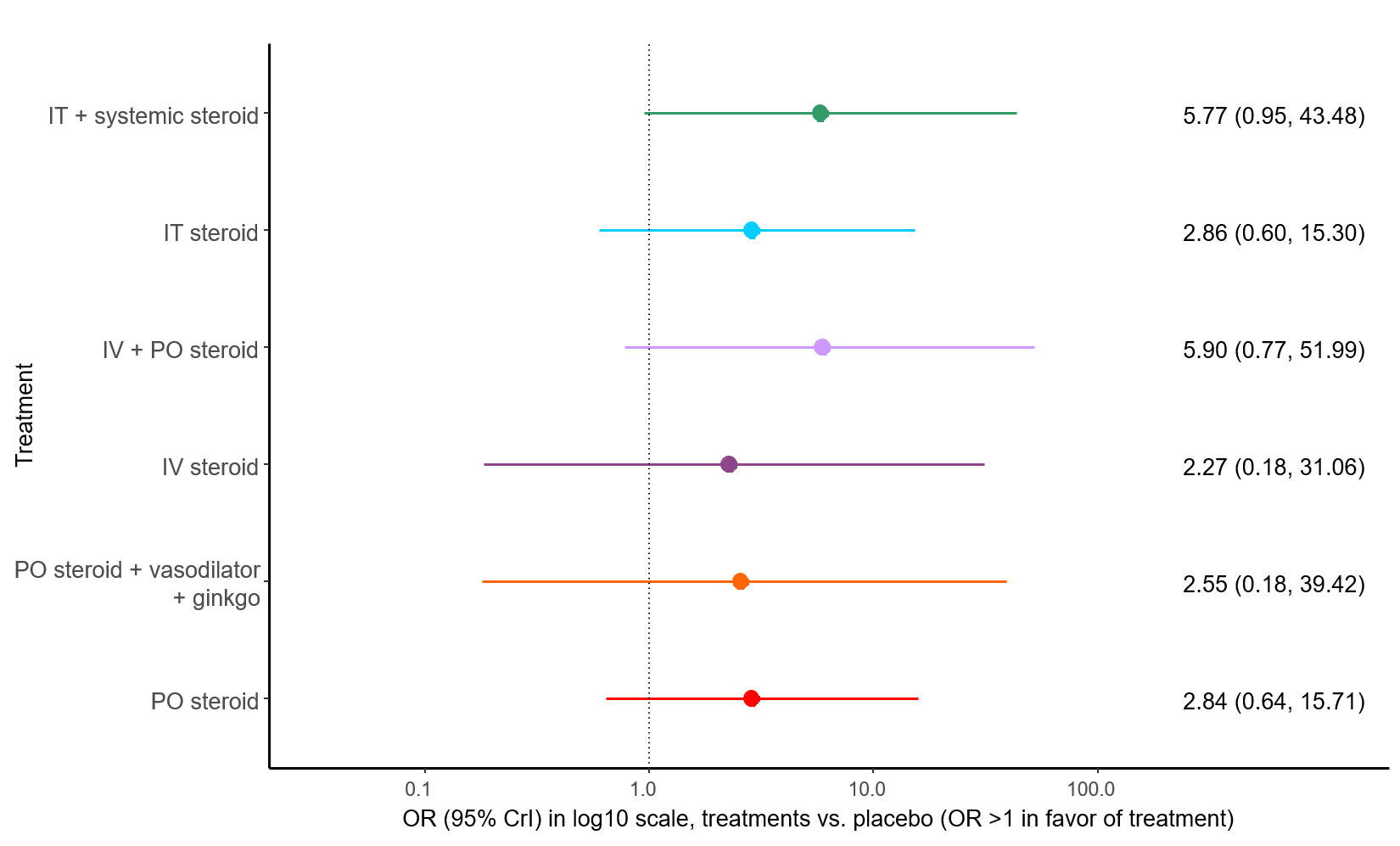


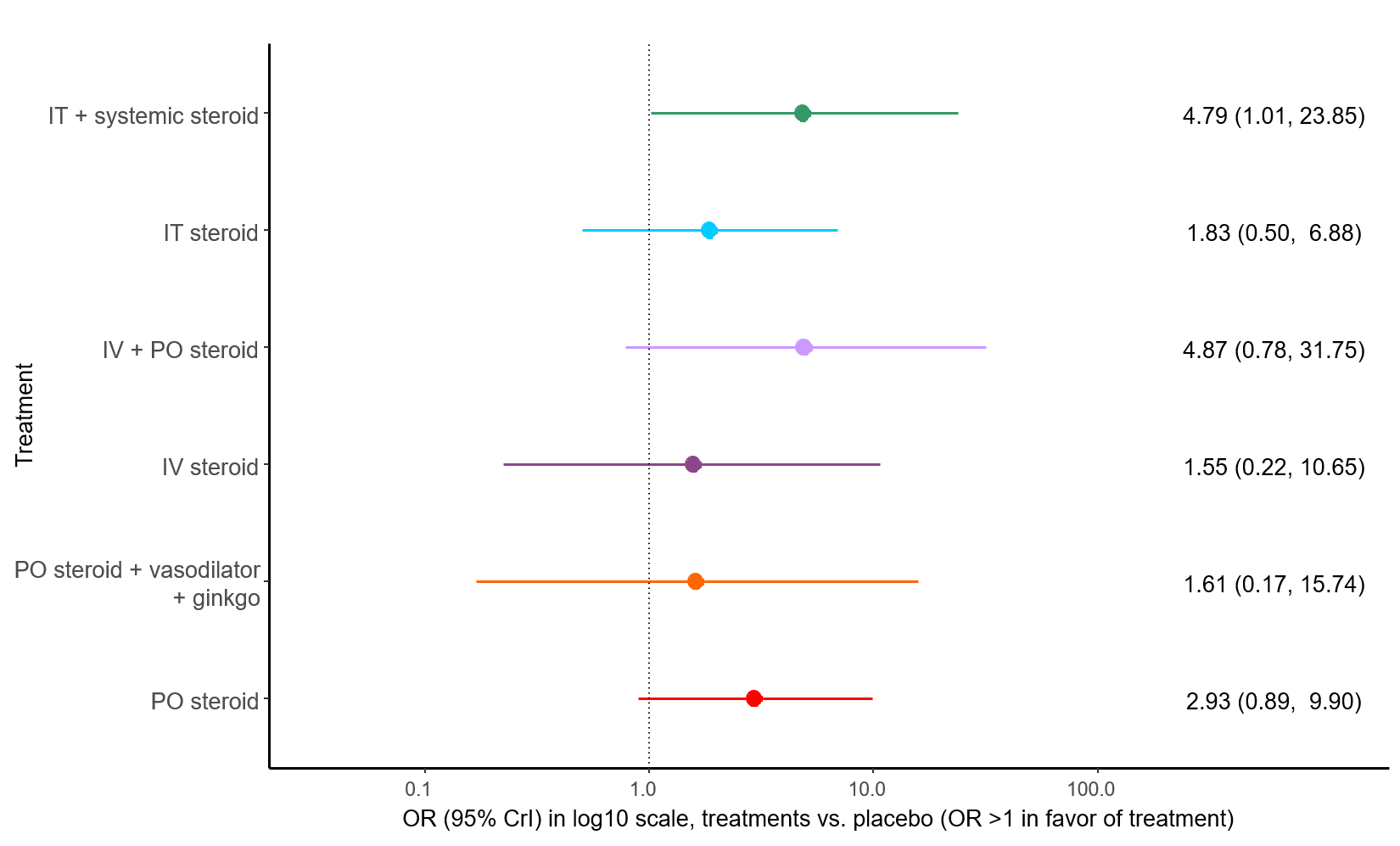

Supplement: S4 Text — (DOCX) [file pone.0221713.s004.docx]
