# Supplementary material for: A systematic review and network meta-analysis of existing pharmacologic therapies in patients with idiopathic sudden sensorineural hearing loss
Source: PLoS One. 2019 Sep 9;14(9):e0221713. doi: 10.1371/journal.pone.0221713 (PMC6733451; doi:10.1371/journal.pone.0221713)

# S5 Text: NMAs Including IT Steroid (As a Salvage Treatment) After Systemic Steroids

For all analyses in the main text, we did not include Park et al. (2011)^39^, which had compared combined use of IT, IV and oral steroids in both arms. IT steroid (as a salvage treatment) after 7 days of systemic steroids was compared with simultaneous IT and systemic steroids, with no significant difference in hearing gain or earlier recovery rate between the two groups. Among the 44 patients in the former group, 15 showed complete or partial recovery within 7 days of systemic treatment and did not receive IT dexamethasone treatment. Park and colleagues recommended the use of IT dexamethasone after 7 days of systemic steroid treatment to reduce the risk associated with unnecessary intratympanic injections.

In this Appendix, we considered IT steroid as (a salvage treatment) after systemic steroids to be a treatment node different from IT plus systemic steroids, and presented the forest plots of its performance compared with other treatments in terms of PTA improvement (**Figure A**), responders’ recovery (**Figure B**), and total recovery (**Figure C**). Please notice that pairwise comparisons other than IT steroid as a salvage treatment after systemic steroids vs IT plus systemic steroids did not have direct evidence from head-to-head trials, and had yielded wide credible intervals. Future trials which investigate systemic steroid(s) followed by IT steroid as a salvage therapy compared with simultaneous IT and systemic steroids or other interventions are necessary.

**Figure A:** Estimated difference of PTA improvement (dB) of IT steroid as a salvage treatment after systemic steroids compared to other treatments from the RE consistency model (with 95% credible intervals). Top: estimates from unadjusted NMA, bottom: estimates at the follow-up time of 60 days from the time-adjusted model.
**
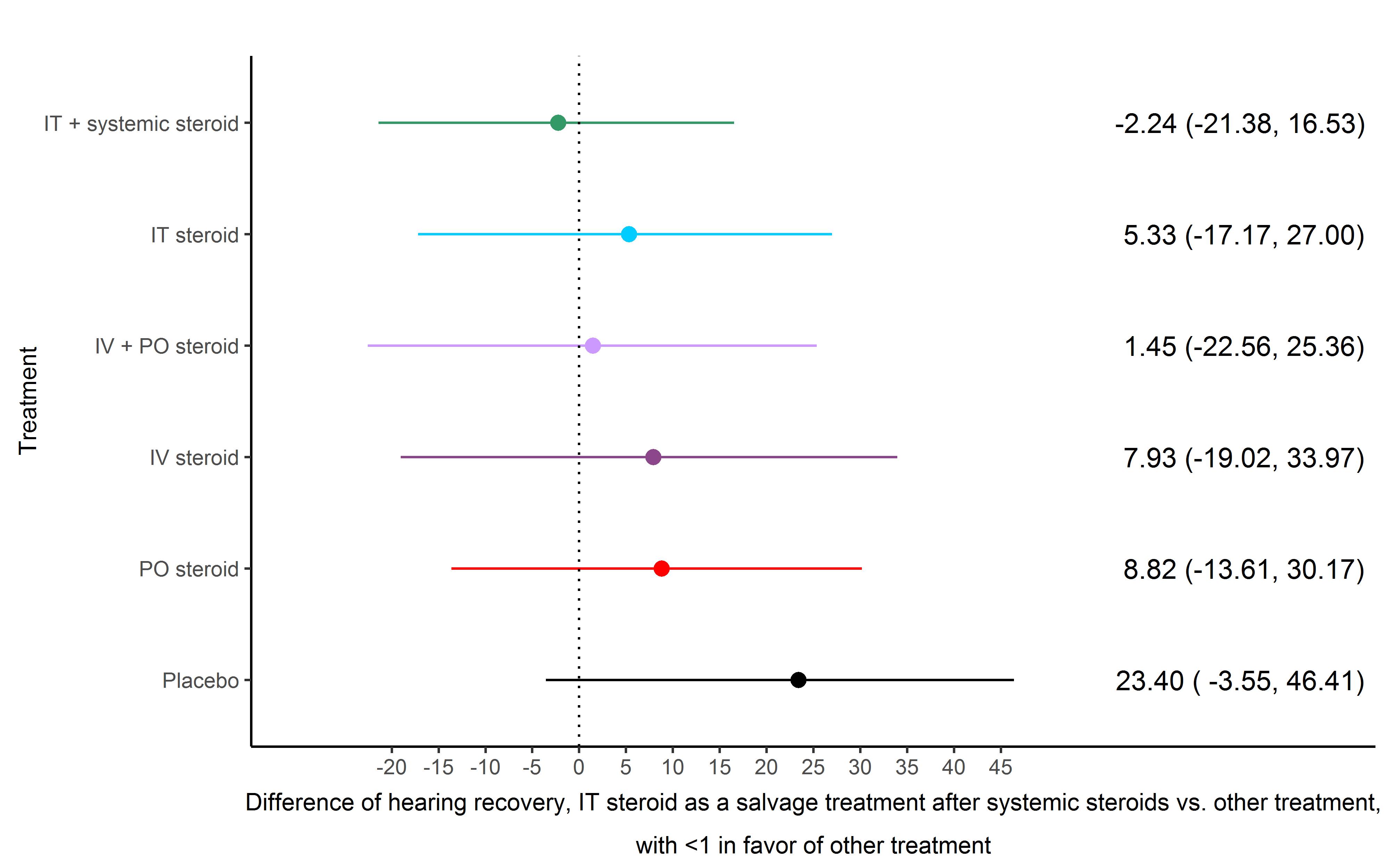


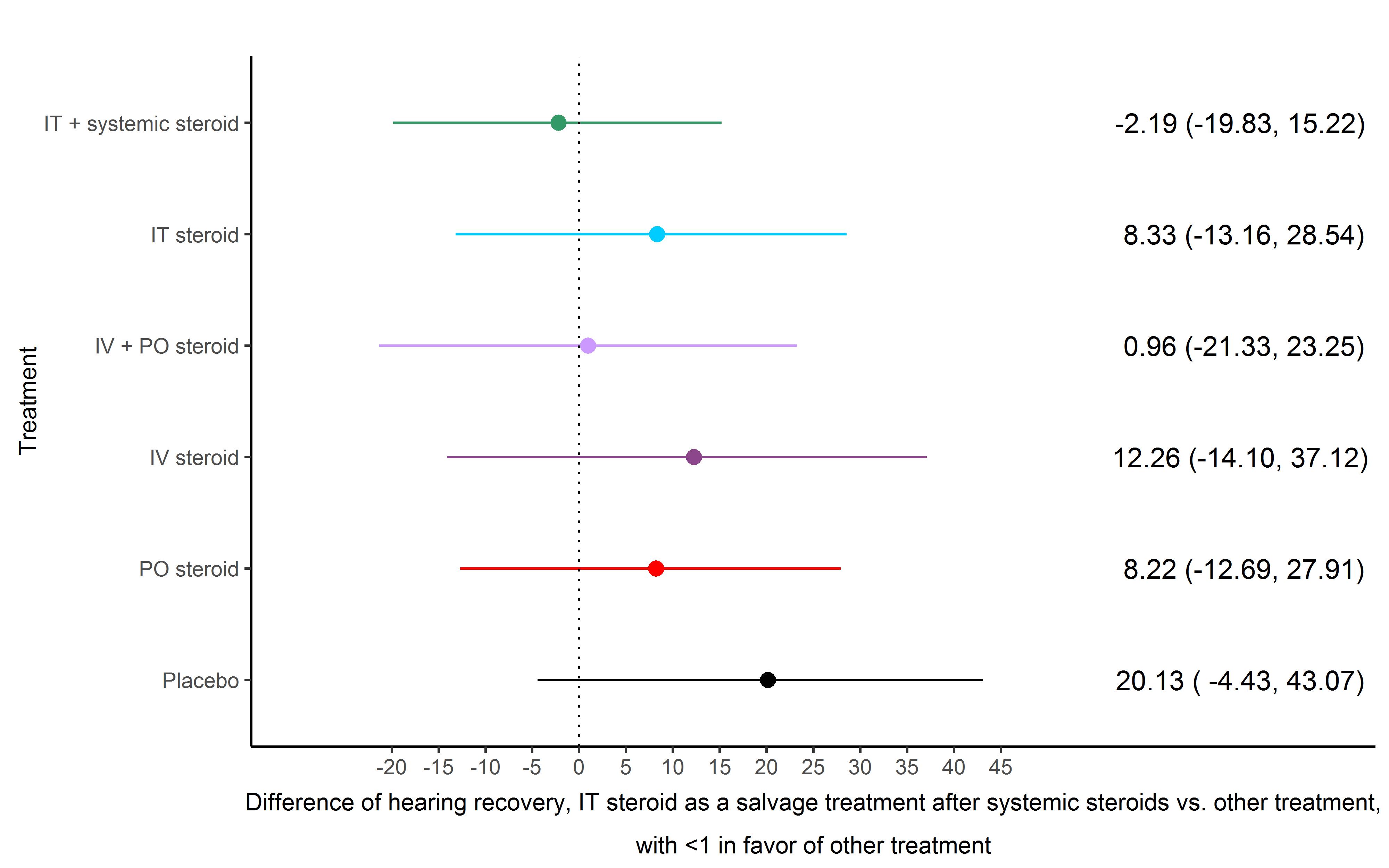
**

**Figure B:** Estimated odds ratio of IT steroid as a salvage treatment after systemic steroids compared to other treatments for responders’ recovery from the RE consistency model (with 95% credible intervals). Top: estimates from unadjusted NMA, bottom: estimates at the follow-up time of 60 days from the time-adjusted model.

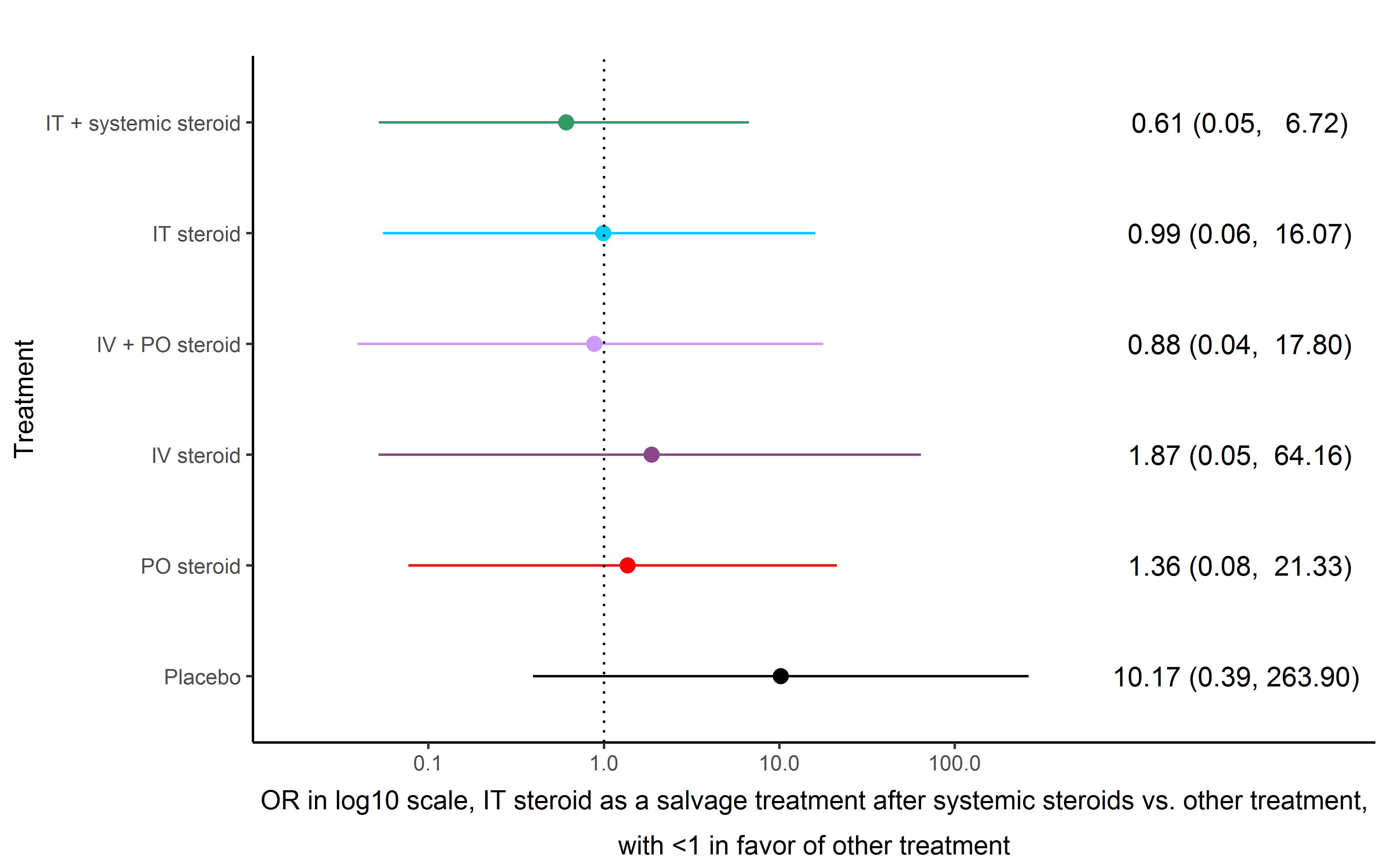


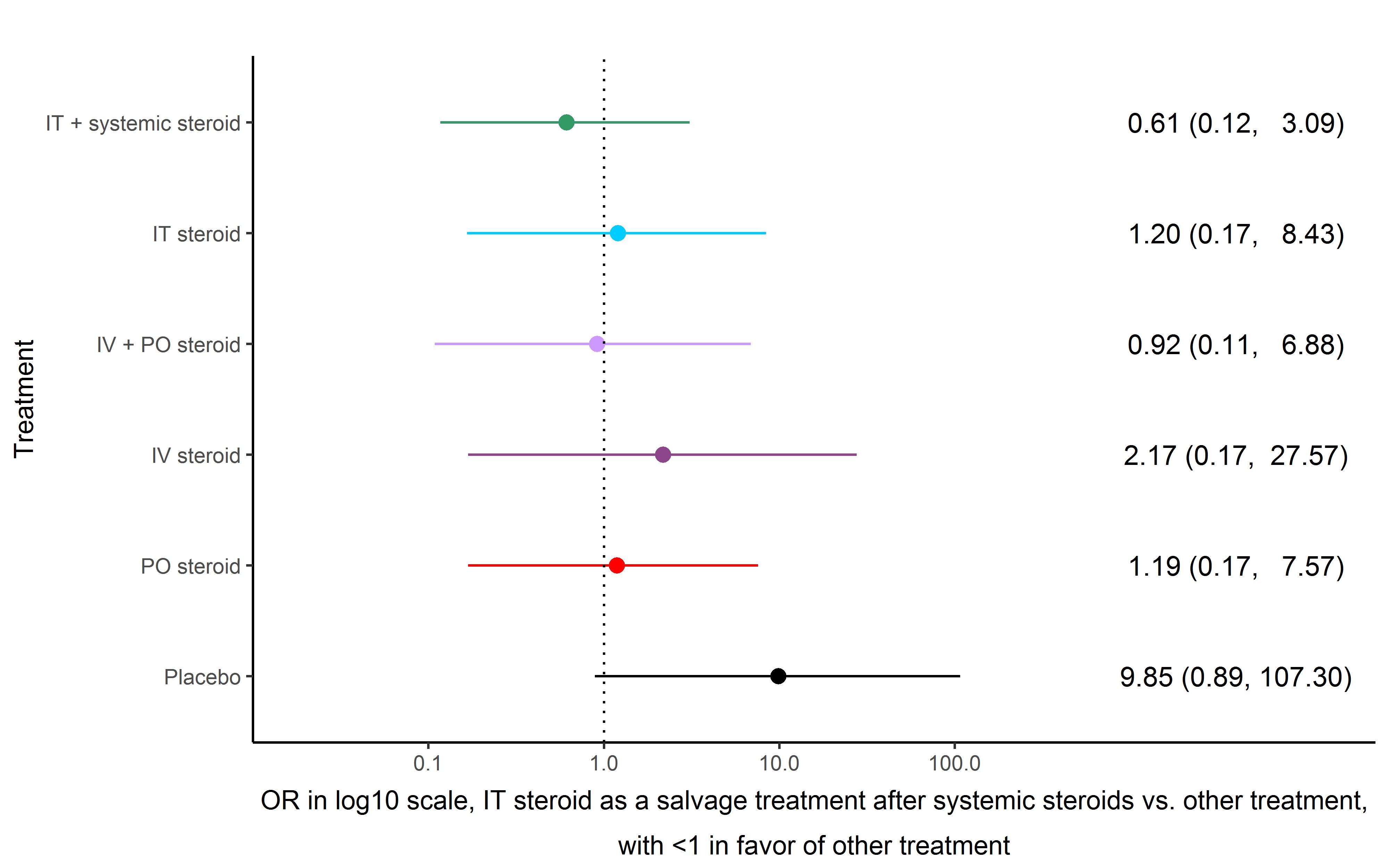


**Figure C:** Estimated odds ratio of IT steroid as a salvage treatment after systemic steroids compared to other treatments for total recovery from the RE consistency model (with 95% credible intervals). Top: estimates from unadjusted NMA, bottom: estimates at the follow-up time of 60 days from the time-adjusted model.

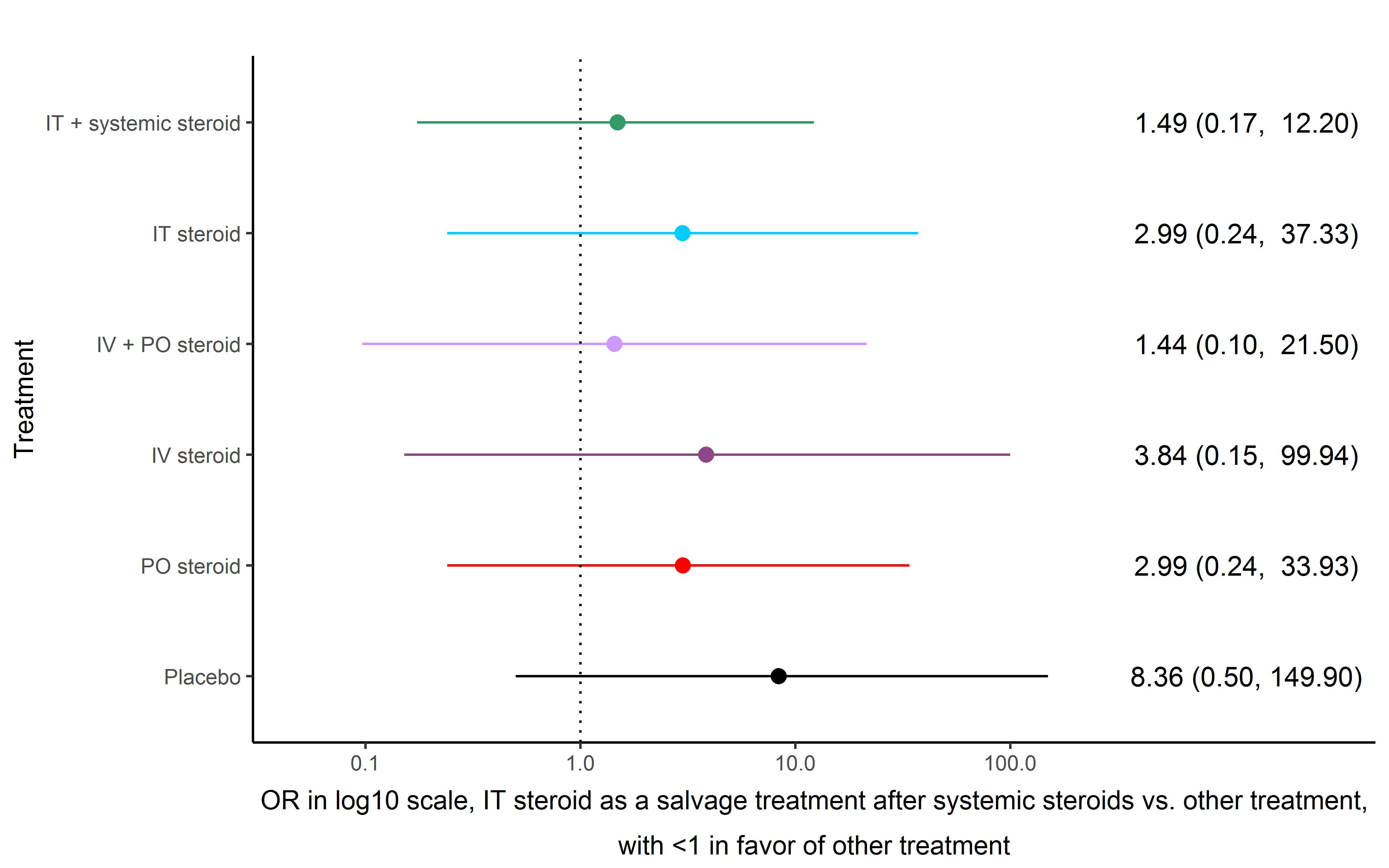


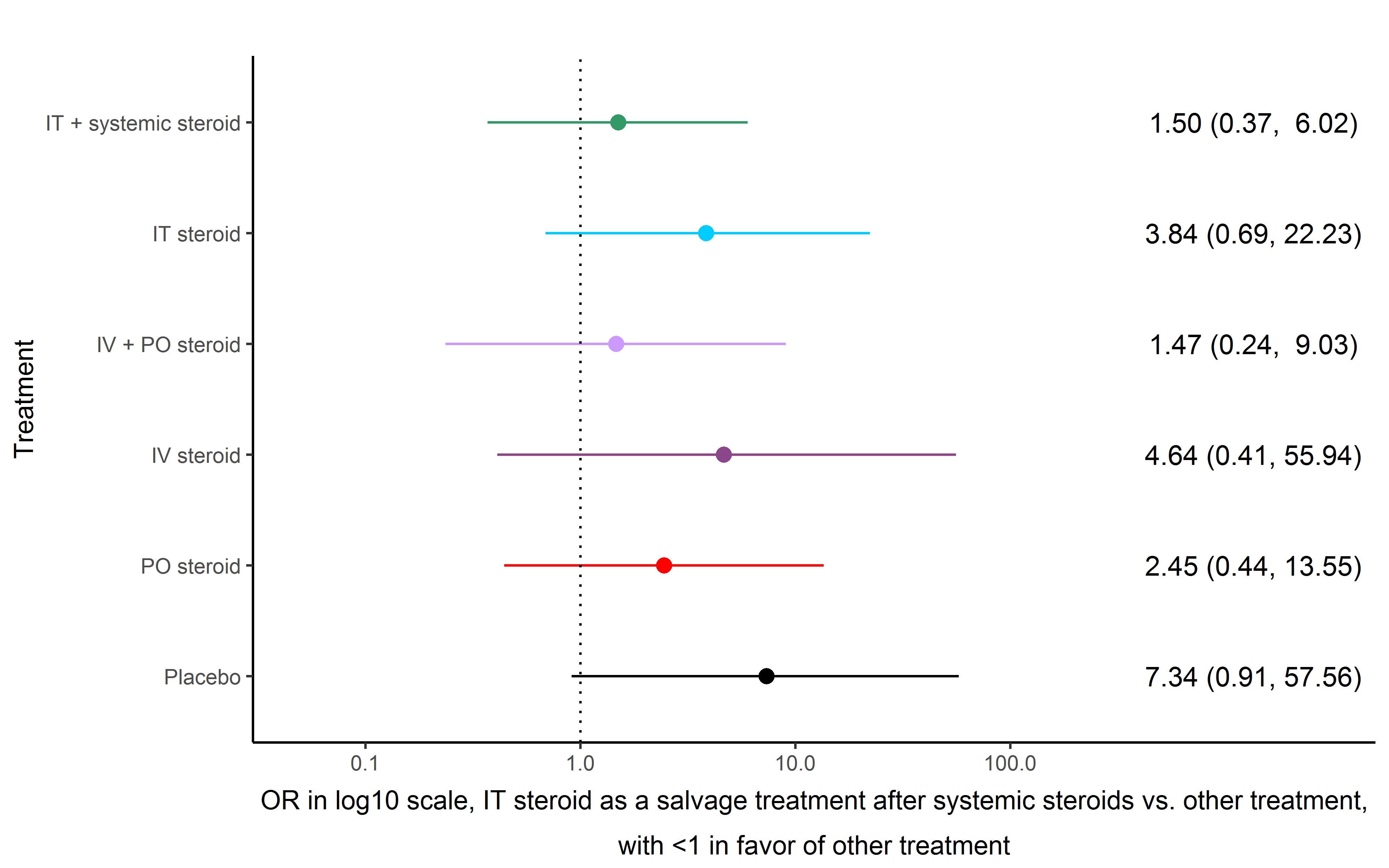

Supplement: S5 Text — (DOCX) [file pone.0221713.s005.docx]
